# Electrochemical-Induced Cascade Reaction of 2-Formyl Benzonitrile with Anilines: Synthesis of *N*-Aryl Isoindolinones

**DOI:** 10.3390/molecules27238199

**Published:** 2022-11-24

**Authors:** Valerio Morlacci, Tonino Caruso, Marco Chiarini, Antonio Arcadi, Massimiliano Aschi, Laura Palombi

**Affiliations:** 1 Dipartimento di Scienze Fisiche e Chimiche, Università degli Studi di L’Aquila, Via Vetoio, 67100 Coppito, Italy; 2 Dipartimento di Chimica e Biologia, Università di Salerno, Via Giovanni Paolo II, 132, 84084 Fisciano, Italy; 3 Dipartimento di Bioscienze e Tecnologie Agroalimentari e Ambientali, Università degli Studi di Teramo, Via R. Balzarini, 64100 Teramo, Italy; 4 Dipartimento di Ingegneria e Scienze dell’Informazione e Matematica, Università degli Studi di L’Aquila, Via Vetoio, 67100 Coppito, Italy

**Keywords:** electrochemical synthesis, anilines, isoindolinone, imine formation, DFT

## Abstract

An electrochemical initiated tandem reaction of anilines with 2-formyl benzonitrile has been developed. Thus, unprecedented 3-*N*-aryl substituted isoindolinones have been conveniently achieved by constant current electrolysis in a divided cell using catalytic amount of electricity and supporting electrolyte and a Pt-cathode as working electrode. The origin of the electrochemical activation as well as the mechanism of the subsequent chemical cascade reactions have been investigated by DFT calculations.

## 1. Introduction

**Figure 1:**
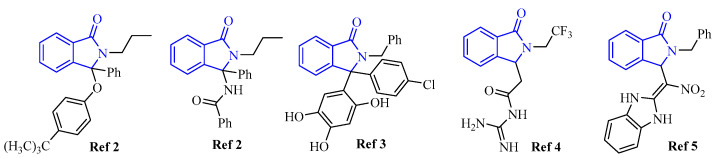
Representative isoindolinones tested for biological activity.

Among nitrogen-containing heterocycles [1], the class of isoindolinones has received considerable interest for decades due to their potential as bioactive ingredients in medicinal chemistry. By way of example, in 2005 an in silico screening of a first generation of isoindolinones highlighted their potential as inhibitors of the MDM2-p53 interaction [2]. Yet, subsequent studies have also shown that introducing other functional groups into the isoindolinone scaffold can significantly improve their pharmacological activity [3,4,5]. Therefore, structural modifications of the isoindolinone motif continue to be the subject of intense investigation for synthetic chemists who face the double challenge of creating new libraries of increasing structural complexity and, at the same time, proposing a sustainable synthesis (Figure 1).

To this regard, we have been exploring for a decade tandem and sequential reactions of 2-formyl benzonitriles succeeding in developing convenient methodologies to access various isoindolinone-containing structures which include the ones with N and S moieties at the exocyclic position [6,7]. Our approaches complement several others that use strategies and synthons designed according to the distinctiveness of the extra functionalities and features of the desired products.

**Scheme 1:**
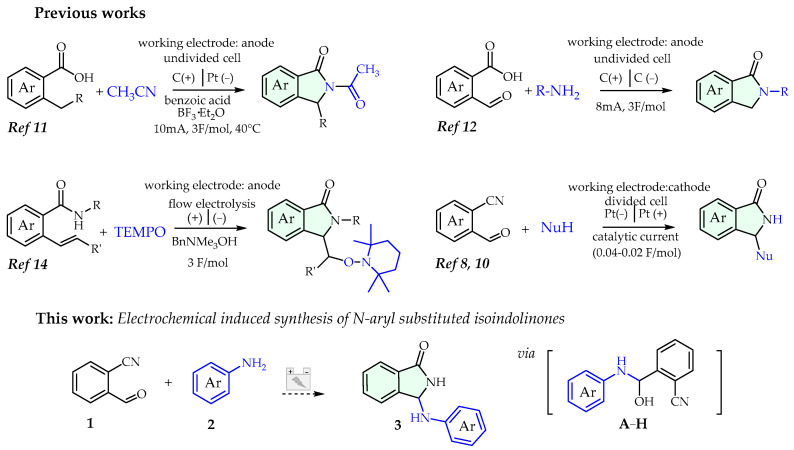
Electrosynthesis of isoindolinones (selected lit. of tandem approaches) and this work.

Besides purely chemical approaches, over the past decades, we [8,9,10] and others [11,12,13,14] also demonstrated the effectiveness of electrocatalysis to promote the synthesis of functionalized isoindolinones: these methods, framed in the picture of electro-organic chemistry renaissance [15,16,17,18], offer several benefits from a synthetic point of view, especially in terms of eco-friendly and waste minimization (Scheme 1).

Based on our previous reports on this topic, we herein report an electrochemical induced tandem reaction of functionalized anilines with 2-formylbenzonitrile to install *N*-aryl substituents in the third position of the isoindolinone nucleus (Scheme 1).

Furthermore, to provide some more quantitative mechanistic insights, we herein explored the potential energy surface of the whole process by means of quantum-chemical calculations in the framework of density functional theory (DFT).

## 2. Results and Discussion

### 2.1. Optimization of Reaction Conditions

According to the Mayr’s scale, despite their low basicity, anilines still exhibit good nucleophilicity parameters toward reference electrophiles [19,20]; however, with respect to the carbonyl addition, aniline hemiaminals are rarely detected in organic solvent due to their marked tendency to release H_2_O yielding imines and, concurrently, because of the low global K_eq_ of this reaction. Consistently, aniline itself proved to serve as nucleophilic catalyst in transimination reaction for oxime and hydrazone synthesis, via aniline Schiff base [21,22]. Indeed, imines derived from anilines are often the focus of various studies of dynamic covalent chemistry [23].

Said the above, to the extent that hamiaminals **A**–**H** are intended as crucial intermediates for the cascade reaction leading to isoindolinones **3** (via cyclization/rearrangement), anilines are quite challenging substrates with respect to alkyl or aliphatic amines in general.

With the aim to attempt an electro-catalyzed process with aromatic amines as nucleophiles, we initiated our investigation by performing the reaction of the model compounds aniline (**2a**), 2-bromoaniline (**2b**) or 2-iodoaniline (**2c**) with the 2-formylbenzonitrile under a variety of electrochemical setup and conditions.

Standard conditions of Table 1 ensured a good 91% yield in the corresponding product **3** using **2a** as nucleophile, while 80% and 57% yield were respectively obtained using the more challenging 2-halogenated anilines **2b** and **2c** which are known to be prone to cathodic dehalogenation [24].

Modifications of the standard reaction conditions such as current quantity/intensity (Table 1, entries 4, 9, 13), concentrations of the reagents (Table 1, entries 4, and 12), solvents (Table 1, entries 5, 6, and 7) etc., as well as variations of the electrochemical setup (divided vs. undivided cells, porosity of the glass separating septum, electrode materials), resulted in diminished yield for all the three products (see also supplementary conditions for further optimization details).

The data reported in Table 1 also show that starting material **1** might undergo extensive decomposition, even applying a current quantity as low as 0.06 F/mol of **1** (Table 1, entry 9). In fact, while under optimized conditions **3** is always observed as the most abundant product with **2a**, **2b,** and **2c**, aldehyde **1** could not be recovered, regardless of whether the applied conditions were effective to yield isolable products. Conversely, a significant recovery of unreacted **2a**–**c** anilines was ascertained in almost all the cases (except Table 1, entry 6). Yet, no better yields have been achieved using anilines **2** as limiting reagents (Table 1, entry 10).

### 2.2. Electrochemically Induced Synthesis of 3-N-Aryl Substituted Isoindolinones

Having optimized the reaction conditions, we evaluated scope and limitation of the electrochemical method by testing the series of compounds reported in Table 2.

As shown, a variety of substituent on the aniline molecule, such as alkyl (Me), alkoxy (OMe, OEt), halogens (Br, Cl, F), and/or functional groups such as alkynyl, cyano, amide, keto, formyl etc., were examined to altogether assess the influence of changes in electron density of the benzene ring, functional group tolerance, and steric hindrance effect.

Noteworthy, with respect to heterogeneous basic catalysis (**3a** and **3u**, Table 2, data in parentheses), the electrochemical method emerges as superior, both in terms of efficiency and reaction times.

Though **2i** and **2t** furnished the corresponding products **3i** and **3t** with barely acceptable yields and no starting materials recovery, we were pleased to find that the electrochemical conditions were compatible with almost all the other anilines, including the ones having ortho- and para-alkynyl (**2t**–**v**) and ortho-benzoyl (**2h**) moieties. Moreover, 2-aminopyridine **2p** also demonstrated a good reactivity under electrochemical conditions, leading to the corresponding hybrid pyridine-isoindolinone **3p** with a 62% yield. It is worth noting that, with respect to the aryl amine, the selectivity is generally high (>85% based on recovered starting material), despite the moderate yields occasionally observed. Moreover, we want to remark the successful attainment of derivatives having sensible functionalities on the aniline moieties such as **3f** (o-CN), **3g** (o-CO_2_NH_2_), and **3i** (o-COMe), useful for further diversification of the molecular structures. Conversely, p-aminobenzaldehyde **2o** failed to yield any product, probably because of the low tolerance of the electrochemical ambient vs. the formyl group. Indeed, both **1** and **2o** partially decomposed under the standard electrochemical conditions. Likewise, the attempt to use 5-bromo-2-formylbenzonitrile (1′) instead of 1 as a reagent with aniline **2a** was unsuccessful. Indeed, extensive decomposition of 1′ occurred under standard electrochemical conditions, while only partial conversion to the corresponding imine 4′a was observed using heterogeneous basic catalysis (conditions reported in Table 2, note c).

### 2.3. Quantum-Chemical Calculations and Plausible Mechanism

**Scheme 2:**
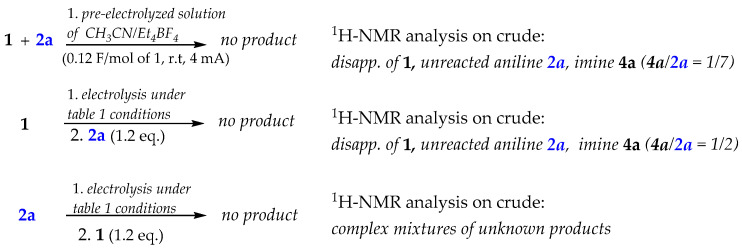
Control experiments of the reaction model **1** + **2a** under different conditions.

To gain an understanding of the electro-induced reaction pathway, we first performed some control experiments on the reaction model **2a** + **1** under various conditions (Scheme 2).

1H-NMR on the crude mixtures clearly indicated that the presence of both the reagents (o-cyanobenzaldehyde and aniline) during the electricity supplying is a strict prerequisite to achieve the desired product **3**.

**Figure 2:**
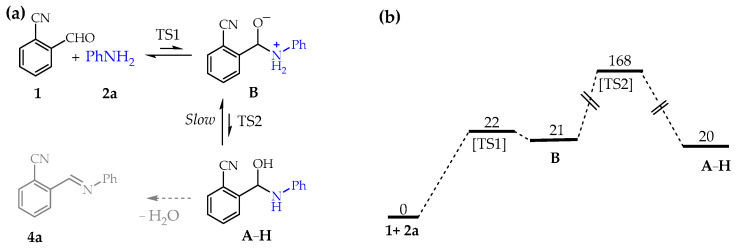
Uncatalyzed hemiaminal formation in CH_3_CN. (**a**) Reaction pathway. (**b**) Standard free-energy values (kJ/mol in CH_3_CN at 298 K).

Thus, we opened our quantum-chemical investigations by analyzing the uncatalyzed nucleophilic addition of the aniline **2a** to the aldehyde **1** in acetonitrile (Figure 2).

Not surprisingly, the uncatalyzed nucleophilic attack of **2a** to **1** to produce the hemiaminal **A**–**H** is predicted as a disfavored process both kinetically and thermodynamically, with the zwitterion **B** present in very low concentration in pre-equilibrium with the reagents.

Consequently, to locate the origin of the electro-activation leading to **3a** both the initial chemical species in the catholyte (i.e., **1**, **2a**, and the solvent CH_3_CN) and the fleeting intermediate **B** occurring during the uncatalyzed route to the imine **4a** have been taken into account as potentially affected by the applied potential.

**Scheme 3:**
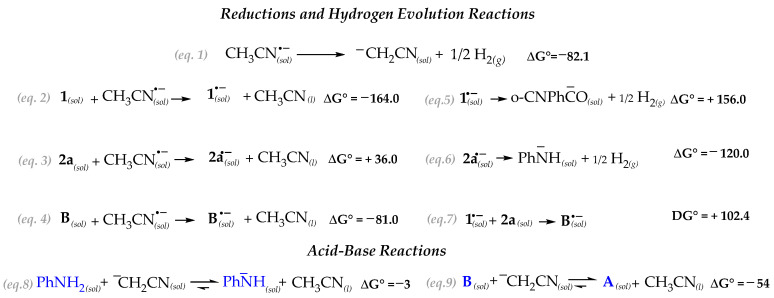
Standard free energy (kJ/mol) at 298 K of the plausible channels initiated by the Pt–electroreduction.

In Scheme 3 we report the energetic of the electro-reductive processes of all the species possibly involved in the reaction, conventionally referring the thermodynamics of the reactions as formally initiated by [CH_3_CN]^−^ since, under constant current conditions, it is the species present in excess.

The ΔG° values clearly suggest that 2-formylbenzonitrile **1** has the highest oxidizing power. However, any process initiated by **1**_(sol)_^−^ (e.g., Equations (5) and (7)), is thermodynamically strongly disfavored. Zwitterionic intermediate **B** is likewise easily reduced (Equation (4)). However, this channel also reveals as totally ineffective due to the strongly thermodynamic driving force leading to **B_(sol)_**^−^ dissociation (reverse of Equation (7)). Therefore, the data suggest that the electrochemical process acting as the reaction trigger is the formation of ¯CH_2_CN_(sol)_ which follows the hydrogen evolution reaction (HER) (Equation (1)) [25]. The electrogenerated strong base ¯CH_2_CN_(sol)_ might undergo to acid-base reaction with either the aniline **2a** (to form the strong nucleophilic aryl amide anion) (Equation (8)) and, concurrently, with the zwitterion **B** (Equation (9)).

**Figure 3:**
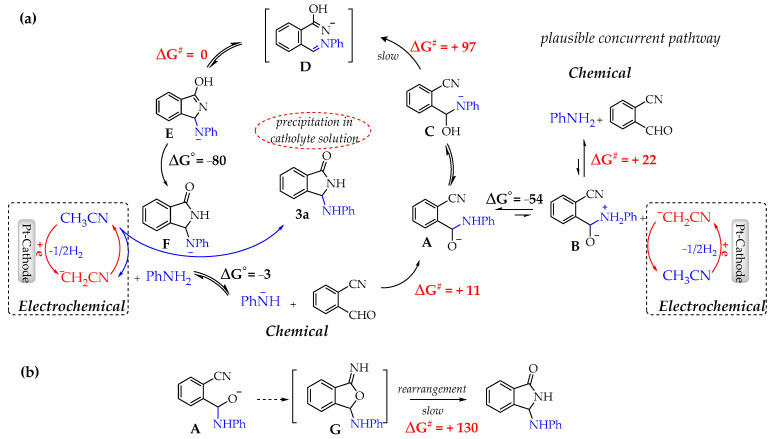
(**a**) Proposed reaction pathways at 298 K in acetonitrile based on DFT calculations. (**b**) Alternative route. ΔG° and ΔG^#^ values are given in kJ/mol.

In Figure 3a is depicted the whole catalytic process which includes the electrochemical initiated cycle and the sequence of cascade reactions leading to the final product **3a**.

As shown, after the electrochemically initiated process and the formation of the crucial anionic intermediate **A**, the sequence of unimolecular H- and HO- transfers leads to **D** which evolves without barrier to **F**, the conjugated base of the final product. After a thermodynamically strongly guided (ΔG° = −50 kJ/mol) acid–base reaction of **F** with CH_3_CN, **3a** is formed and the base ¯CH_2_CN, able to re-initializing the catalytic cycle, restored. It is also equally reasonable that the acid–base reaction of electrogenerated base ¯CH_2_CN and zwitterion B contributes to the formation of the intermediate A.

Conversely, we want finally to remark that DFT calculations ruled out the possible alternative pathway involving **A** closure and rearrangement of the intermediate **G**. As shown in Figure 3b, the rearrangement step would imply higher activation energy.

## 3. Materials and Methods

### 3.1. General Information

Electrochemical reactions were conducted using Hewlett Packard DC Power Supply Mod. E3612A in constant current mode, in a U-divided glass cell separated through a porous G-3 glass plug. Platinum spirals (apparent area 1 cm^2^) were used as anode and cathode (distance between the electrodes 1 cm). Before using, Pt electrodes were treated with a Piranha solution (sulfuric acid/hydrogen peroxide 3:1) for 1 min, washed with double-distilled water and sonicated three times for 5 min with double-distilled water, acetone, and isopropanol. The reactions were monitored by thin layer chromatography (TLC) using Merck Silica Gel 60 F254 plates and were visualized by fluorescence quenching at 254 nm. Column chromatographic purification of products was carried out using silica gel 60 (70–230 mesh, Merck). The NMR spectra were recorded on Bruker Avance 400 spectrometers (400 MHz, ^1^H; 101 MHz, ^13^C). Spectra were referenced to residual CHCl_3_ (7.26 ppm, ^1^H; 77.00 ppm, ^13^C), MeOD (3.31 ppm, ^1^H; 49 ppm, ^13^C) or DMSO (2.50 ppm, ^1^H; 39.5 ppm, ^13^C) when indicated. Yields are given for isolated products showing one spot on a TLC plate and seldom impurities detectable in the NMR spectrum. High-resolution mass spectra (HRMS) were acquired using a Bruker SolariX XR Fourier transform ion cyclotron resonance mass spectrometer (Bruker Daltonik GmbH, Bremen, Germany) equipped with a 7 T refrigerated actively shielded superconducting magnet. The samples were ionized in positive ion mode using an electrospray (ESI) ionization source or the MALDI ion source.

### 3.2. Materials

All chemicals and solvents were obtained from commercial sources and were used without further purification. Pt electrodes (wires, wire, diam. 0.5 mm, 99.99% trace metals basis) were purchased from Sigma-Aldrich.

### 3.3. Procedure for Electrosynthesis of Compounds **3**

A solution of **1** (0.2 mmol), **2** (0.24 mmol), and tetraethylammonium tetrafluoroborate (Et_4_NBF_4_) (0.08 mmol) in MeCN (0.4 mL) is added in the cathodic compartment of a U-divided cell equipped with platinum spirals (apparent area 1 cm^2^) as cathode (WE, working electrode) and anode (CE, counter electrode). Catholyte was constituted by a solution of Et_4_NBF_4_ (0.1 mmol) in MeCN (0.5 mL). Electrolysis was conducted under galvanostatic conditions (4 mA, 0.12 electrons/molecule of **1**) at r.t. At the end of the electrolysis, TLC analysis showed disappearance of **1** and the reaction was in any case prolonged at r.t. under magnetic stirring for 6 h. The mixture was then concentrated in vacuum and directly purified by silica gel chromatography (Hexane: Ethyl Acetate from 4:1 to 3:2) to afford the desired products **3a**–**3v.**

*3-(phenylamino) isoindolin-1-one (***3a***)* [26]: Prepared following general procedure using **1** (0.2 mmol, 26 mg) and aniline **2a** (0.24 mmol, 22 mg). The crude was purified directly by flash chromatography to give a white solid **3a** (41 mg, 91%). ^1^H-NMR (400 MHz, CDCl_3_) δ = 7.87 (d, *J* = 7.5 Hz, 1H, Ar), 7.62 (d, *J* = 4.1 Hz, 2H, Ar), 7.60–7.52 (m, 1H, Ar), 7.28 (d, *J* = 7.9 Hz, 2H, Ar), 6.89 (t, *J* = 7.4 Hz, 1H, Ar), 6.79 (d, *J* = 8.0 Hz, 2H, Ar), 6.60 (s, 1H, CONH), 6.19 (d, *J* = 10.7 Hz, 1H, CH), 4.11 (d, *J* = 10.7 Hz, 1H, NH). ^13^C-NMR (101 MHz, DMSO) δ = 168.8; 146.8; 145.7; 132.6; 131.8; 129.0; 128.8; 123.7; 122.5; 117.3; 113.5; 64.7. HR-MS (MALDI) m/z calcd for C_14_H_13_N_2_O [M + H^+^] 225.1022, found 225.1009, m/z calcd for C_14_H_12_N_2_ONa [M + Na^+^] 247.0841, found 247.0827.

*3-((2-bromophenyl) amino) isoindolin-1-one (***3b***):* Prepared following general procedure using **1** (0.2 mmol, 26 mg) and 2-bromoaniline **2b** (0.24 mmol, 41 mg). The crude was purified directly by flash chromatography to give a yellow solid **3b** (48 mg, 80%). ^1^H-NMR (400 MHz, CDCl_3_) δ = 7.88 (d, *J* = 7.3 Hz, 1H, Ar); 7.67–7.53 (m, 3H, Ar); 7.49 (d, *J* = 8.0 Hz, 1H, Ar); 7.30–7.16 (m, 1H, Ar); 6.94 (s, 1H, CONH); 6.89 (d, *J* = 8.1 Hz, 1H, Ar), 6.73 (t, *J* = 7.7 Hz, 1H, Ar); 6.17 (d, *J* = 9.7 Hz, 1H, CH); 4.85 (d, *J* = 9.7 Hz, 1H, NH). ^13^C-NMR (101 MHz, CDCl_3_) δ = 169.6; 144.2; 142.6; 133.3; 132.8; 132.0; 129.9; 128.8; 124.0; 123.4; 120.5; 112.7; 111.2; 65.4. HR-MS (MALDI) m/z calcd for C_14_H_12_BrN_2_O [M + H^+^] 303.0127, found 303.0110.

*3-((2-iodophenyl) amino) isoindolin-1-one (***3c***):* Prepared following general procedure using **1** (0.2 mmol, 26 mg) and 2-iodoaniline **2c** (0.24 mmol, 52 mg). The crude was purified directly by flash chromatography to give a yellow solid **3c** (40 mg, 57%). ^1^H-NMR (400 MHz, CDCl_3_) δ = 7.89 (dd, *J* = 7.4, 1.2 Hz, 1H, Ar); 7.74 (dd, *J* = 7.9, 1.5 Hz, 1H, Ar); 7.68–7.55 (m, 3H, Ar); 7.29 (d, *J* = 7.7 Hz, 1H, Ar); 6.83 (d, *J* = 8.1 Hz, 1H, Ar); 6.74 (s, 1H, CONH); 6.61 (t, *J* = 7.6 Hz, 1H, Ar); 6.17 (d, *J* = 9.6 Hz, 1H, CH); 4.69 (d, *J* = 9.6 Hz, 1H, NH). ^13^C-NMR (101 MHz, CDCl_3_) δ = 169.5; 145.0; 144.2; 139.9; 132.8; 131.9; 130.0; 129.8; 124.0; 123.4; 121.3; 112.1; 87.0; 65.8. HR-MS (MALDI) m/z calcd for C_14_H_12_IN_2_O [M + H^+^] 350.9988, found 350.9966.

*3-((2-chlorophenyl) amino) isoindolin-1-one (***3d***):* Prepared following general procedure using **1** (0.2 mmol, 26 mg) and 2-chloroaniline **2d** (0.24 mmol, 30 mg). Th crude was purified directly by flash chromatography to give a yellow solid **3c** (31 mg, 61%). ^1^H-NMR (400 MHz, CDCl_3_) δ = 7.86 (dt, *J* = 7.5, 1.1 Hz, 1H, Ar); 7.66–7.51 (m, 3H, Ar); 7.49 (s, 1H, CONH); 7.33–7.22 (m, 1H, Ar); 7.23–7.13 (m, 1H, Ar); 6.92 (dd, *J* = 8.3, 1.4 Hz, 1H, Ar); 6.77 (td, *J* = 7.7, 1.4 Hz, 1H, Ar); 6.16 (d, *J* = 9.7 Hz, 1H, CH); 4.85 (d, *J* = 9.7 Hz, 1H, NH). ^13^C-NMR (101 MHz, CDCl_3_) δ = 169.6; 144.3; 141.6; 132.8; 132.0; 130.0; 129.9; 128.1; 124.0; 123.4; 120.7; 120.0; 112.6; 65.2. HR-MS (MALDI) m/z calcd for C_14_H_12_ClN_2_O [M + H^+^] 259.0632, found 259.0617.

*3-((2-methoxyphenyl) amino) isoindolin-1-one (***3e***):* Prepared following general procedure using **1** (0.2 mmol, 26 mg) and 2-methoxyaniline **2e** (0.24 mmol, 29 mg). The crude was purified directly by flash chromatography to give a yellow solid **3e** (39 mg, 78%). ^1^H-NMR (400 MHz, CDCl_3_) δ = 7.87 (d, *J* = 7.4 Hz, 1H, Ar); 7.68–7.49 (m, 3H, Ar); 6.98–6.79 (m, 4H, Ar); 6.72 (s, 1H, CONH); 6.19 (d, *J* = 8.0 Hz, 1H, CH); 4.74 (d, *J* = 8.0 Hz, 1H, NH); 3.80 (s, 3H, OCH_3_). ^13^C-NMR (101 MHz, CDCl_3_) δ = 169.5; 147.7; 144.8; 135.2; 132.5; 132.0; 129.7; 123.9; 123.6; 121.4; 119.4; 111.4; 110.5; 65.4; 55.5. HR-MS (MALDI) m/z calcd for C_15_H_15_N_2_O_2_ [M + H^+^] 255.1128, found 255.1112.

*2-((3-oxoisoindolin-1-yl) amino) benzonitrile (***3f***):* Prepared following general procedure using **1** (0.2 mmol, 26 mg) and 2-cyanoaniline **2f** (0.24 mmol, 28 mg). The crude was purified directly by flash chromatography to give a yellow solid **3f** (29 mg, 59%). ^1^H-NMR (400 MHz, CDCl_3_) δ = 7.88 (d, *J* = 7.3 Hz, 1H, Ar); 7.69–7.55 (m, 3H, Ar); 7.47 (m, 2H, Ar); 7.11 (s, 1H, CONH); 6.94–6.83 (m, 2H, Ar); 6.21 (d, *J* = 9.2 Hz, 1H, CH); 5.07 (d, *J* = 9.2 Hz, 1H, NH). ^13^C-NMR (101 MHz, CDCl_3_) δ = 169.7; 147.8; 143.5; 134.6; 133.4; 133.0; 131.8; 130.2; 124.2; 123.4; 119.3; 117.1; 112.0; 98.4; 64.8. HR-MS (MALDI) m/z calcd for C_15_H_12_N_3_O [M + H^+^] 250.0974, found 250.0962.

*2-((3-oxoisoindolin-1-yl) amino) benzamide (***3g***):* Prepared following general procedure using **1** (0.2 mmol, 26 mg) and 2-aminobenzamide **2g** (0.24 mmol, 32 mg). The crude was purified directly by flash chromatography to give a white solid **3g** (42 mg, 79%). ^1^H-NMR (400 MHz, MeOD) δ = 7.79 (d, *J* = 7.5 Hz, 1H, Ar); 7.71–7.61 (m, 2H, Ar); 7.57 (t, *J* = 7.5 Hz, 1H, Ar); 7.45 (d, *J* = 7.6 Hz, 1H, Ar); 7.19 (t, *J* = 7.7 Hz, 1H, Ar); 6.76 (d, *J* = 8.3 Hz, 1H, Ar); 6.72 (s, 1H, CH); 6.57 (t, *J* = 7.6 Hz, 1H, Ar). ^13^C NMR (101 MHz, DMSO) δ 169.4; 168.9; 150.1; 145.4; 132.6; 132.2; 131.9; 128.9; 128.5; 123.5; 122.5; 116.4; 114.4; 113.4; 60.4. HR-MS (MALDI) m/z calcd for C_15_H_14_N_3_O_2_ [M + H^+^] 268.1080, found 268.1145

*3-((2-benzoylphenyl) amino) isoindolin-1-one (***3h***):* Prepared following general procedure using **1** (0.2 mmol, 26 mg) and 2-aminobenzophenone **2h** (0.24 mmol, 47 mg). The crude was purified directly by flash chromatography to give a yellow solid **3h** (49 mg, 75%). ^1^H-NMR (400 MHz, CDCl_3_) δ = 8.81 (d, *J* = 8.4 Hz, 1H, NH); 7.88 (d, *J* = 7.4 Hz, 1H, Ar); 7.68–7.38 (m, 10H, Ar); 7.07 (s, 1H, CONH); 6.99 (d, *J* = 8.4 Hz, 1H, Ar); 6.75 (t, *J* = 7.6 Hz, 1H, Ar); 6.26 (d, *J* = 8.4 Hz, 1H, CH). ^13^C-NMR (101 MHz, CDCl_3_) δ = 199.4; 169.7; 149.2; 144.3; 139.7; 135.7; 135.1; 132.8; 131.9; 131.5; 129.9; 129.3; 128.2; 124.0; 123.4; 119.5; 116.6; 112.0; 64.3. HR-MS (MALDI) m/z calcd for C_21_H_17_N_2_O_2_ [M + H^+^] 329.1284, found 329.1264.

*3-((2-acetylphenyl) amino) isoindolin-1-one (***3i***):* Prepared following general procedure using **1** (0.2 mmol, 26 mg) and 2-aminoacetophenone **2i** (0.24 mmol, 32 mg). The crude was purified directly by flash chromatography to give a yellow solid **3i** (24 mg, 45%). ^1^H-NMR (400 MHz, CDCl_3_) δ = 9.35 (d, *J* = 8.4 Hz, 1H, NH); 7.88 (d, *J* = 7.3 Hz, 1H, Ar); 7.84 (dd, *J* = 8.1, 1.5 Hz, 1H, Ar); 7.66–7.54 (m, 3H, Ar); 7.47–7.40 (m, 1H, Ar); 6.89 (d, *J* = 8.4 Hz, 1H, Ar); 6.84–6.77 (m, 1H, Ar); 6.72 (s, 1H, CONH); 6.22 (d, *J* = 8.4 Hz, 1H, CH); 2.59 (s, 3H, CH_3_). ^13^C-NMR (101 MHz, CDCl_3_) δ = 201.3; 169.6; 148.7; 144.4; 135.3; 133.1; 132.8; 129.8, 124.0; 123.3; 119.4; 116.9; 111.9; 64.1; 28.1. HR-MS (MALDI) m/z calcd for C_16_H_15_N_2_O_2_ [M + H^+^] 267.1128, found 267.1111.

*3-((4-chlorophenyl) amino) isoindolin-1-one (***3j***):* Prepared following general procedure using **1** (0.2 mmol, 26 mg) and 4-chloroaniline **2j** (0.24 mmol, 30 mg). The crude was purified directly by flash chromatography to give a white solid **3i** (40 mg, 79%). ^1^H-NMR (400 MHz, MeOD) δ = 7.79 (d, *J* = 7.5 Hz, 1H, Ar); 7.70–7.60 (m, 2H, Ar); 7.57 (t, *J* = 7.3 Hz, 1H, Ar); 7.13 (d, *J* = 8.4 Hz, 2H, Ar); 6.77 (d, *J* = 8.4 Hz, 2H, Ar); 6.19 (s, 1H, CH). ^13^C-NMR (101 MHz, MeOD) δ = 172.5; 146.9; 146.8; 133.7; 133.4; 130.5; 130.0; 124.9; 124.2; 124.2; 116.5; 67.3. HR-MS (MALDI) m/z calcd for C_14_H_12_ClN_2_O [M + H^+^] 259.0632, found 259.0618.

*3-((4-fluorophenyl) amino) isoindolin-1-one (***3k***):* Prepared following general procedure using **1** (0.2 mmol, 26 mg) and 4-fluoroaniline **2k** (0.24 mmol, 26 mg). The crude was purified directly by flash chromatography to give a yellow solid **3k** (34 mg, 71%). ^1^H-NMR (400 MHz, MeOD) δ = 7.78 (d, *J* = 7.5 Hz, 1H, Ar); 7.65 (d, *J* = 6.4 Hz, 2H, Ar); 7.56 (t, *J* = 7.0 Hz, 1H, Ar); 6.90 (t, *J* = 8.6 Hz, 2H, Ar); 6.84–6.73 (m, 2H, Ar); 6.16 (s, 1H, CH). ^13^C-NMR (101 MHz, MeOD) δ = 178.4; 165.9; 163.6; 155.1; 152.9; 142.1; 141.4; 138.6; 133.3; 132.1; 124.9; 124.7; 124.3; 74.9. HR-MS (MALDI) m/z calcd for C_14_H_12_FN_2_O [M + H^+^] 243.0928, found 243.0913.

*3-(p-tolylamino) isoindolin-1-one (***3l***):* Prepared following general procedure **A** using **1** (0.2 mmol, 26 mg) and p-toluidine **2l** (0.24 mmol, 26 mg). The crude was purified directly by flash chromatography to give a white solid **3l** (34 mg, 72%). ^1^H-NMR (400 MHz, CDCl_3_) δ = 7.85 (d, *J* = 7.4 Hz, 1H, Ar); 7.65–7.51 (m, 3H, Ar); 7.07 (d, *J* = 7.8 Hz, 2H, Ar); 6.70 (d, *J* = 7.8 Hz, 2H, Ar); 6.64 (s, 1H, CONH); 6.14 (d, *J* = 8.6 Hz, 1H, CH); 3.96 (d, *J* = 8.6 Hz, 1H, NH); 2.28 (s, 3H, CH_3_). ^13^C-NMR (101 MHz, CDCl_3_) δ = 169.5; 144.7; 143.0; 132.5; 132.0; 130.3; 129.8; 129.6; 123.9; 123.5; 114.7; 66.3; 20.5. HR-MS (MALDI) m/z calcd for C_15_H_15_N_2_O [M + H^+^] 239.1178, found 239.1166.

*3-((4-methoxyphenyl) amino) isoindolin-1-one (***3m***):* Prepared following general procedure using **1** (0.2 mmol, 26 mg) and 4-methoxyaniline **2m** (0.24 mmol, 29 mg). The crude was purified directly by flash chromatography to give a brown solid **3m** (39 mg, 78%). ^1^H-NMR (400 MHz, CDCl_3_) δ = 7.85 (d, *J* = 7.4 Hz, 1H, Ar); 7.65–7.49 (m, 3H, Ar); 6.84 (d, *J* = 8.7 Hz, 2H, Ar); 6.75 (d, *J* = 8.7 Hz, 2H, Ar); 6.67 (s, 1H, CONH); 6.06 (s, 1H, CH); 3.89–3.77 (m, 4H, OCH_3_ + NH). ^13^C-NMR (101 MHz, CDCl_3_) δ = 169.5; 154.1; 144.7; 139.0; 132.5; 131.9; 129.7; 123.9; 123.6; 116.6; 115.3; 67.2; 55.7. HR-MS (MALDI) m/z calcd for C_15_H_15_N_2_O_2_ [M + H^+^] 255.1128, found 255.1119.

*3-((4-ethoxyphenyl) amino) isoindolin-1-one (***3n***):* Prepared following general procedure using **1** (0.2 mmol, 26 mg) and 4-ethoxyaniline **2n** (0.24 mmol, 33 mg). The crude was purified directly by flash chromatography to give a yellow solid **3n** (37 mg, 70%). ^1^H-NMR (400 MHz, CDCl_3_) δ = 7.85 (d, *J* = 7.4 Hz, 1H, Ar); 7.64–7.48 (m, 3H, Ar); 6.86–6.81 (m, 2H, Ar); 6.77–6.71 (m, 2H, Ar); 6.65 (s, 1H, CONH); 6.07 (s, 1H, CH); 3.99 (q, *J* = 7.0 Hz, 2H, CH_2_); 3.81 (s, 1H, NH); 1.40 (t, *J* = 7.0 Hz, 3H, CH_3_). ^13^C-NMR (101 MHz, CDCl_3_) δ = 169.5; 153.4; 144.7; 138.9; 132.5; 131.9; 129.7; 123.9; 123.6; 116.6; 116.1; 67.2; 64.0. HR-MS (MALDI) m/z calcd for C_16_H_17_N_2_O_2_ [M + H^+^] 269.1284, found 269.1271.

*3-(pyridin-2-ylamino) isoindolin-1-one (***3p***):* Prepared following general procedure using **1** (0.2 mmol, 26 mg) and 2-aminopyridine **2p** (0.24 mmol, 22 mg). The crude was purified directly by flash chromatography to give a yellow solid **3p** (28 mg, 62%). ^1^H-NMR (400 MHz, CDCl_3_) δ = 8.23–8.12 (m, 1H, Ar); 7.83 (d, *J* = 7.5 Hz, 1H, Ar); 7.60 (d, *J* = 4.1 Hz, 2H, Ar); 7.57–7.42 (m, 2H, Ar); 7.06 (s, 1H, CONH); 6.79–6.70 (m, 1H, Ar); 6.57 (d, *J* = 8.8 Hz, 1H, CH); 6.51 (d, *J* = 8.3 Hz, 1H, Ar); 4.93 (d, *J* = 8.8 Hz, 1H, NH). ^13^C-NMR (101 MHz, CDCl_3_) δ = 169.2; 157.1; 148.1; 144.7; 137.8; 132.5; 132.4; 129.7; 124.4; 123.9; 123.2; 115.1; 109.8; 63.4. HR-MS (MALDI) m/z calcd for C_13_H_12_N_3_O [M + H^+^] 226.0974, found 226.0971.

*3-((2-bromo-4-methylphenyl) amino) isoindolin-1-one (***3q***):* Prepared following general procedure using **1** (0.2 mmol, 26 mg) and 2-bromo-4-methylaniline **2q** (0.24 mmol, 44 mg). The crude was purified directly by flash chromatography to give a yellow solid **3q** (42 mg, 67%). ^1^H-NMR (400 MHz, CDCl_3_) δ = 7.87 (d, *J* = 7.4 Hz, 1H, Ar); 7.61 (m, 2H, Ar); 7.57 (d, *J* = 7.4 Hz, 1H, Ar); 7.31 (s, 1H, Ar); 7.03 (d, *J* = 8.2 Hz, 1H, Ar); 6.94 (s, 1H, CONH); 6.79 (d, *J* = 8.2 Hz, 1H, Ar); 6.12 (d, *J* = 9.8 Hz, 1H, CH); 4.68 (d, *J* = 9.8 Hz, 1H, NH); 2.25 (s, 3H, CH_3_). ^13^C-NMR (101 MHz, CDCl_3_) δ = 169.6; 144.4; 140.2; 133.5; 132.7; 131.9; 130.4; 129.9; 129.3; 123.9; 123.4; 113.0; 111.3; 65.8; 20.1. HR-MS (MALDI) m/z calcd for C_15_H_14_N_2_OBr [M + H^+^] 317.0284, found 317.0265.

*3-((2,4-dimethylphenyl) amino) isoindolin-1-one (***3r***):* Prepared following general procedure using **1** (0.2 mmol, 26 mg) and 2,4-dimethylaniline **2r** (0.24 mmol, 29 mg). The crude was purified directly by flash chromatography to give a yellow solid **3r** (32 mg, 64%). ^1^H-NMR (400 MHz, CDCl_3_) δ = 7.87 (d, *J* = 7.4, 1H, Ar), 7.68–7.58 (m, 2H, Ar), 7.56 (m, 1H, Ar), 7.05–6.90 (m, 2H, Ar), 6.82 (m, 2H, Ar + CONH), 6.16 (s, 1H, CH), 3.86 (s, 1H, NH), 2.27 (s, 3H, CH_3_), 2.11 (s, 3H, CH_3_). ^13^C-NMR (101 MHz, CDCl_3_) δ = 169.6; 145.1; 141.2; 132.7; 132.0; 129.9; 129.3; 127.8; 124.3; 124.0; 123.6; 112.1; 112.0; 66.2; 20.5; 17.6. HR-MS (MALDI) m/z calcd for C_16_H_17_N_2_O [M + H^+^] 253.1335, found 253.1325.

*3-((2,5-dimethoxyphenyl) amino) isoindolin-1-one (***3s***):* Prepared following general procedure using **1** (0.2 mmol, 26 mg) and 2,5-dimethoxyaniline **2s** (0.24 mmol, 36 mg). The crude was purified directly by flash chromatography to give a white solid **3s** (34 mg, 61%). ^1^H-NMR (400 MHz, CDCl_3_) δ = 7.86 (d, *J* = 7.4 Hz, 1H, Ar); 7.67–7.48 (m, 3H, Ar); 6.80 (s, 1H, CONH); 6.73 (d, *J* = 8.8 Hz, 1H, Ar); 6.46 (d, *J* = 2.8 Hz, 1H, Ar); 6.31 (dd, *J* = 8.8, 2.8 Hz, 1H, Ar); 6.14 (d, *J* = 10.2 Hz, 1H, CH); 4.77 (d, *J* = 10.2 Hz, 1H, NH); 3.75 (d, *J* = 1.3 Hz, 6H, OCH_3_). ^13^C-NMR (101 MHz, CDCl_3_) δ = 169.5; 154.7; 144.7; 142.1; 136.2; 132.5; 132.1; 129.7; 123.9; 123.5; 111.1; 101.9; 99.6; 65.2; 56.0; 55.7. HR-MS (MALDI) m/z calcd for C_16_H_17_N_2_O_3_ [M + H^+^] 285.1233, found 285.1216.

*3-((2-ethynylphenyl) amino) isoindolin-1-one (***3t***):* Prepared following general procedure using **1** (0.2 mmol, 26 mg) and 2-((trimethylsilyl)ethynyl)aniline **2t** (0.24 mmol, 45 mg). The crude was purified directly by flash chromatography to give a yellow solid **3t** (26 mg, 53%). ^1^H-NMR (400 MHz, CDCl_3_) δ = 7.89 (d, *J* = 7.3 Hz, 1H, Ar); 7.68–7.60 (m, 2H, Ar); 7.60–7.54 (m, 1H, Ar); 7.41 (dd, *J* = 7.7, 1.6 Hz, 1H, Ar); 7.28 (d, *J* = 1.6 Hz, 1H, Ar); 6.88–6.77 (m, 2H, Ar); 6.72 (s, 1H, CONH); 6.22 (d, *J* = 9.6 Hz, 1H, CH); 5.11 (d, *J* = 9.6 Hz, 1H, NH); 3.33 (s, 1H, CH). ^13^C-NMR (101 MHz, CDCl_3_) δ = 169.5, 146.9; 144.4; 133.4; 132.8; 131.9; 130.5; 129.9; 124.0; 123.4; 118.9; 110.8; 108.5; 83.7; 79.8; 65.1. HR-MS (MALDI) m/z calcd for C_16_H_13_N_2_O [M + H^+^] 249.1022, found 249.1008.

*3-((2-(phenylethynyl) phenyl) amino) isoindolin-1-one (***3u***):* Prepared following general procedure using **1** (0.2 mmol, 26 mg) and 2-(phenylethynyl) aniline **2u** (0.24 mmol, 46 mg). The crude was purified directly by flash chromatography to give a yellow solid **3u** (44 mg, 69%). ^1^H-NMR (400 MHz, CDCl_3_) δ = 7.89 (d, *J* = 7.4 Hz, 1H, Ar); 7.68–7.63 (m, 2H, Ar); 7.62–7.54 (m, 1H, Ar); 7.45 (d, *J* = 7.6 Hz, 1H, Ar); 7.40–7.35 (m, 2H, Ar); 7.32–7.26 (m, 4H, Ar); 6.88–6.80 (m, 3H, Ar + CONH); 6.23 (d, *J* = 9.5 Hz, 1H, CH); 5.13 (d, *J* = 9.5 Hz, 1H, NH). ^13^C-NMR (101 MHz, CDCl_3_) δ = 169.6; 146.1; 144.6; 132.9; 132.8; 132.0; 131.5; 130.1; 129.9; 128.5; 128.4; 128.4; 124.0; 123.3; 122.8; 119.1; 111.1; 109.8; 95.8; 85.1; 65.4. HR-MS (MALDI) m/z calcd for C_22_H_17_N_2_O [M + H^+^] 325.1335, found 325.1318.

*3-((4-(phenylethynyl) phenyl) amino) isoindolin-1-one (***3v***):* Prepared following general procedure using **1** (0.2 mmol, 26 mg) and 4-(phenylethynyl) aniline **2v** (0.24 mmol, 46 mg). The crude was purified directly by flash chromatography to give a yellow solid **3v** (45 mg, 70%). ^1^H-NMR (400 MHz, DMSO) δ = 9.06 (s, 1H, CONH); 7.70 (d, *J* = 7.4 Hz, 1H, Ar); 7.68–7.60 (m, 1H, Ar); 7.61–7.52 (m, 2H, Ar); 7.48 (d, *J* = 7.4 Hz, 2H, Ar); 7.38 (q, *J* = 8.2, 7.3 Hz, 3H, Ar); 7.31 (d, *J* = 8.2 Hz, 2H, Ar); 6.85 (d, *J* = 9.3 Hz, 1H, CH); 6.81 (d, *J* = 8.3 Hz, 2H, Ar); 6.24 (d, *J* = 9.3 Hz, 1H, NH). ^13^C NMR (101 MHz, DMSO) δ = 169.3; 147.9; 145.8; 133.0; 132.9; 132.5; 131.4; 129.7; 129.1; 128.4; 124.3; 123.7; 123.1; 113.9; 110.7; 91.2; 87.6; 64.7. HR-MS (MALDI) m/z calcd for C_22_H_17_N_2_O [M + H^+^] 325.1335, found 325.1314.

### 3.4. Computational Details

All the calculations were performed with the Gaussian 09 [27] program in the framework of the density functional theory (DFT) using the functional wB97XD functional using a version of Grimme’s D2 dispersion model [28] in conjunction with different basis sets: geometry optimizations were carried out with the 6–31G*. Energies were then refined through single point calculations adding a diffusion function with the 6–31 + G* basis set. All the structures were optimized in the gas phase and characterized through the calculation of the mass-weighted Hessian matrix, as minima (all positive eigenvalues of the Hessian matrix) or transition structures (1 negative eigenvalue of the Hessian matrix). The gas-phase Gibbs molar free energy (G_X,gas_) was then calculated, using the previous geometries and harmonic frequencies, for each species in the gas phase at 25 °C at the concentration of 1M using the standard statistical-mechanical relations. Finally, the solvation, i.e., excess, molar free energy (G_X,solv_) was calculated within the mean-field approximation in acetonitrile using the polarizable conductor calculation model [29]. Within this approximations the molar free energy (G_X_) for each species in solution corresponds to the usual equation

G°_X_ = G°_X,gas_ + G_X,solv_ + RT ln [X]

where [X] = 1.0 M for all the species in solution and [X] = D_X_:MW_X_ (where D is the density of the species X) for the solvent, i.e., acetonitrile. For H_2_ the standard state corresponding to 1.0 bar of pressure was used. All the cartesian coordinates of the optimized geometries are collected in the Supplementary Information.

## 4. Conclusions

In summary, performing constant current electrolysis with catalytic amount of electricity, we accessed unprecedented molecular architectures that encompass isoindolinone nucleus and functionalized anilines, two substructures playing relevant roles in the production of pharmaceuticals.

Mild conditions, catalytic loading of supporting electrolyte [30], short electrolysis/reaction time, as well as acceptable functional group tolerance are the major strong points of this synthetic approach.

Finally, the mechanistic insight offered by DFT computations, allowed us to provide a consistent picture of the effectiveness of the electrochemical activation to the generation of the highly nucleophilic aryl amide anion species that follows the HER of the solvent on Pt cathode.

## Figures and Tables

**Table 1:** Optimization of the reaction conditions ^(a)^.

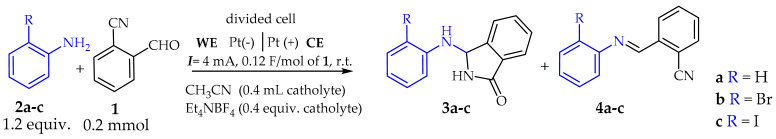					
Entry	*Variations from* *Standard Conditions*	1	2	3	4
		%Conv ^(b)^	%Conv ^(c)^	%Yield ^(d)^	%Yield ^(d)^
1	none	>98	**2a** 80	**3a** 91	**4a** -
2	Undivided cell	>98	**2a** 25	**3a ** *traces*	**4a** 25
3	No electricity ^(e)^	57 ^(f)^	**2a** 48	**3a** -	**4a** 57 ^(f)^
4	*I* = 8 mA, CH_3_CN: 0.6 mL	>98	**2a** 61	**3a** 68	**4a** -
5	CH_3_CH_2_CN instead of CH_3_CN	>98	**2a** 51	**3a** 22	**4a** 28
6	DMF instead of CH_3_CN	>98	**2a** 74	**3a** 37	**4a** -
7	MeOH instead of CH_3_CN	>98	**2a** <2	**3a** -	**4a ** *traces*
8	none	>98	**2b** 70	**3b** 80	**4b** -
9	Q = 0.06 F/mol	>98	**2b** 61	**3b** 47	**4b** -
10	*Ratio* **1**:**2b** = 1.2:1	>98	**2b** 67	**3b** 61	**4b** -
11	none	>98	**2c** 62	**3c** 57	**4c** -
12	CH_3_CN: 0.8 mL	>98	**2c** 44	**3c** 31	**4c** -
13	Q = 0.35 F/mol	>98	**2c** >98	**3c** 38	**4c** -
14	Electrolysis at 0 °C	>98	**2c** 72	**3c** 38	**4c** -

^(a)^ After the electrolysis (standard conditions highlighted in the scheme above), the anolyte was removed and the reaction prolonged under stirring for 6 h at r.t. ^(b)^ Conversions have been determined by ^1^HNMR analysis of the crude mixture. ^(c)^ Conversions have been determined based on recovered **2a**–**c** (isolated). ^(d)^ Isolated Yield. ^(e)^ Solvent: CD_3_CN. ^(f)^ Conversion of **1** and yield of **4a** were established by ^1^HNMR analysis after 6 h.

**Table 2:** Synthesis of 3-*N*-aryl substituted isoindolinones ^(a,b)^.

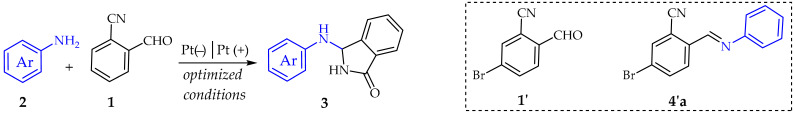
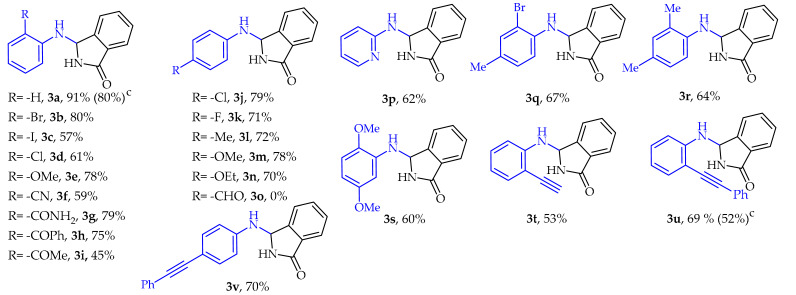

^(a)^ Standard conditions (see Table 1). ^(b)^ Isolated Yield. ^(c)^ Isolated yield by chemical reaction, 1 eq. of K_2_CO_3_ as base, CH_3_CN, 72 h, r.t.

## Data Availability

Not applicable.

## Supplementary Materials

The following supporting information can be downloaded at: https://www.mdpi.com/article/10.3390/molecules27238199/s1, Figure S1: The setup of electrochemical reaction; Table S1: Optimization studies; Figures S2–S10: **3a** structure determination (2D-NMR Spectra). 1H, 13C NMR spectra of the products; DFT Data.
