# Supplementary material for: Electrochemical-Induced Cascade Reaction of 2-Formyl Benzonitrile with Anilines: Synthesis of N-Aryl Isoindolinones
Source: Molecules. 2022 Nov 24;27(23):8199. doi: 10.3390/molecules27238199 (PMC9738245; doi:10.3390/molecules27238199)
Supplement: Supplementary file 1 [file molecules-27-08199-s001.zip › molecules-2043697-supplementary.pdf]

Supporting information

Electrochemical-induced cascade reaction of 2-formyl benzonitrile with anilines: synthesis of N-aryl substituted isoindolinones

Valerio Morlacci[a)], Tonino Caruso[b)], Marco Chiarini[c)], Antonio Arcadi[d)], Massimiliano Aschi[a)] and Laura Palombi[a)]*

[a)] Dipartimento di Scienze Fisiche e Chimiche; Università degli Studi di L'Aquila; Via Vetoio, 67100 Coppito (AQ), Italy

[b)] Dipartimento di Chimica e Biologia, Università di Salerno, via Giovanni Paolo II, 132, 84084 Fisciano (Sa), Italy

[c)] Dipartimento di Bioscienze e Tecnologie Agroalimentari e Ambientali, Università degli Studi di Teramo, via R. Balzarini, 1- 64100 Teramo, Italy

[d)] Dipartimento di Ingegneria e Scienze dell'Informazione e Matematica; Università degli Studi di L'Aquila; Via Vetoio, 67100 Coppito (AQ), Italy

Table of Contents

The setup of electrochemical reaction S-2

Optimization studies S-3

^{1}H-^{13}C NMR Spectra S-4

3a structure determination (2D-NMR Spectra) S-25

DFT Data S-32

The setup of electrochemical reaction

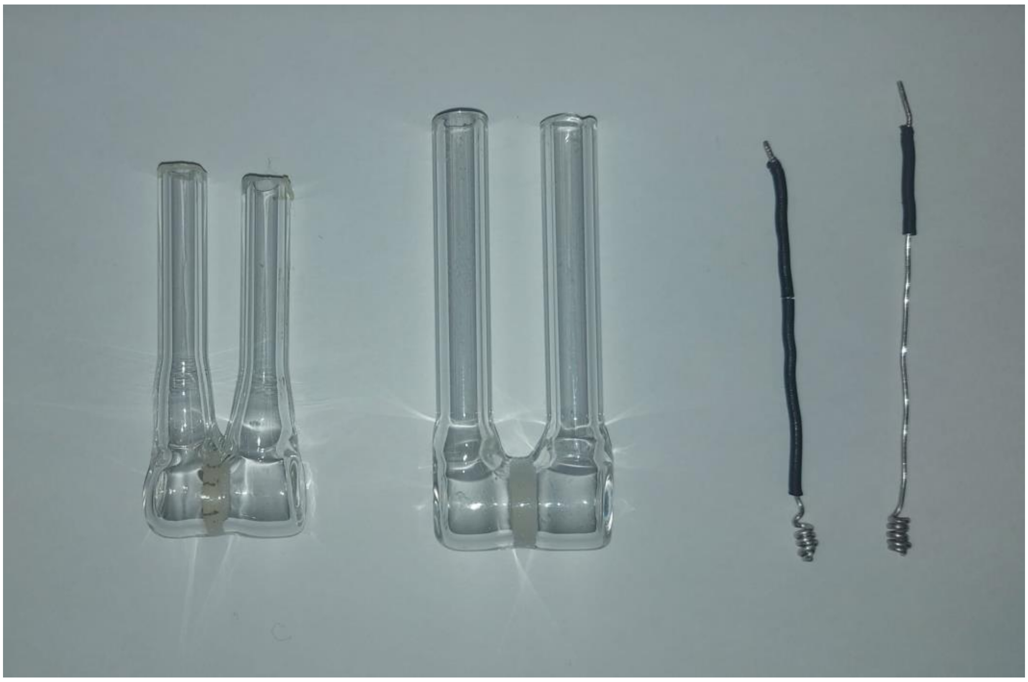

Figure S-1

Optimization studies

Table S1. Optimization of the Reaction Conditions

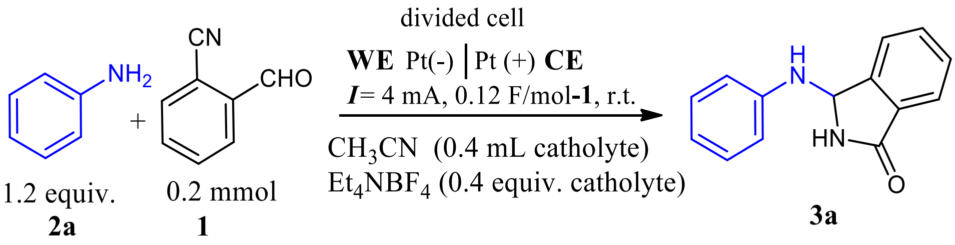

Entry	Deviation from standard conditions	Yield %[a]
1	**None**	91
2	Pd as cathode	88
3	Au as cathode	86
4	Glassy Carbon as cathode	traces
5	Al as cathode	45
6	Et_4NPF_6 as supporting electrolyte	82
7	*n*-Bu_4NBF_4 as supporting electrolyte	88
8	[EMIM][ESO_4] as supporting electrolyte	32
9	Et_4NBF_4 1 eq	90
10	Et_4NBF_4 0,2 eq	67
11	Porous G-5 glass plug instead of G-3	86
12	Pre-electrolysis of **2a** (6%) and second electrolysis (6%) after addition of **1**	20

[a]Yields of isolated products. **WE**: Working Electrode. **CE**: Counter Electrode.

NMR SPECTRA

*3-(phenylamino) isoindolin-1-one (**3a**)*

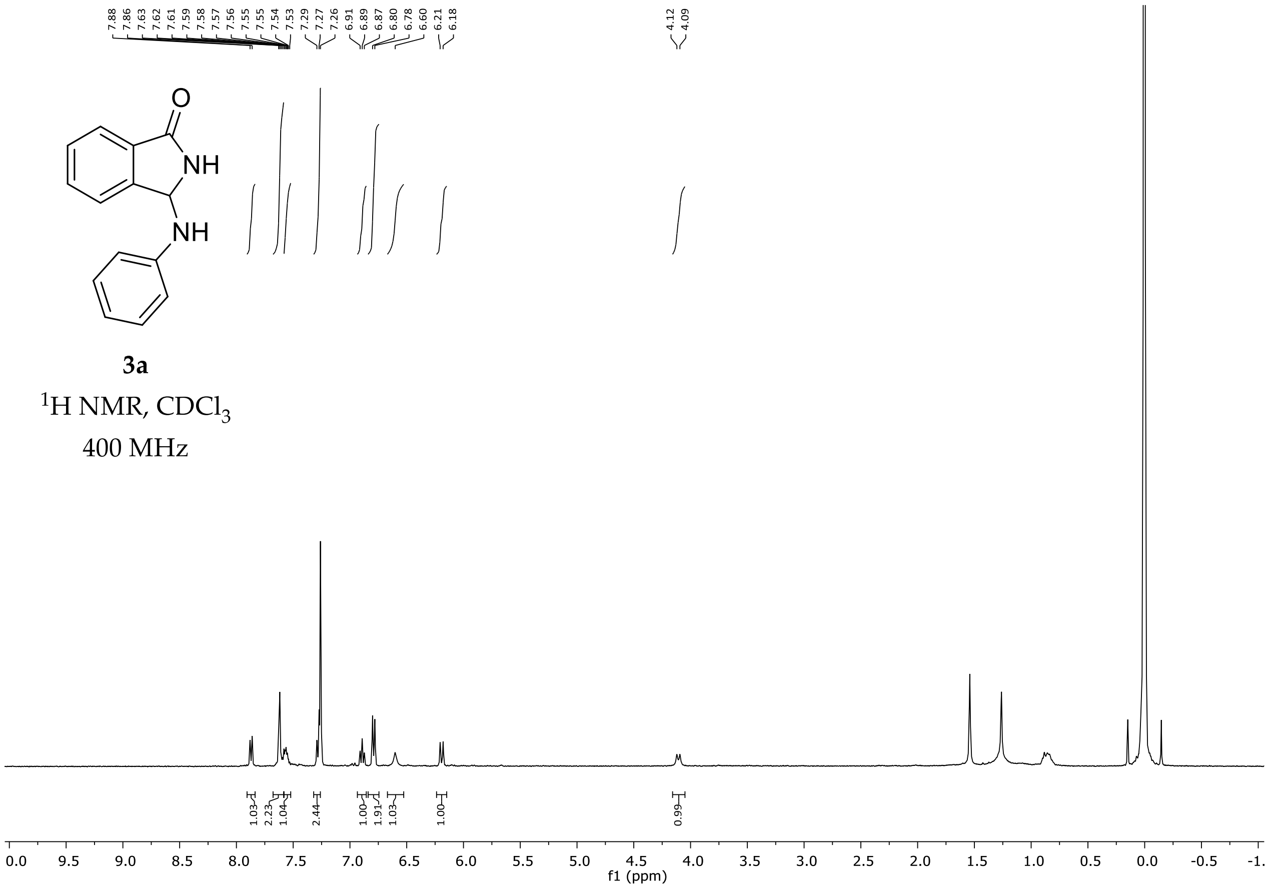

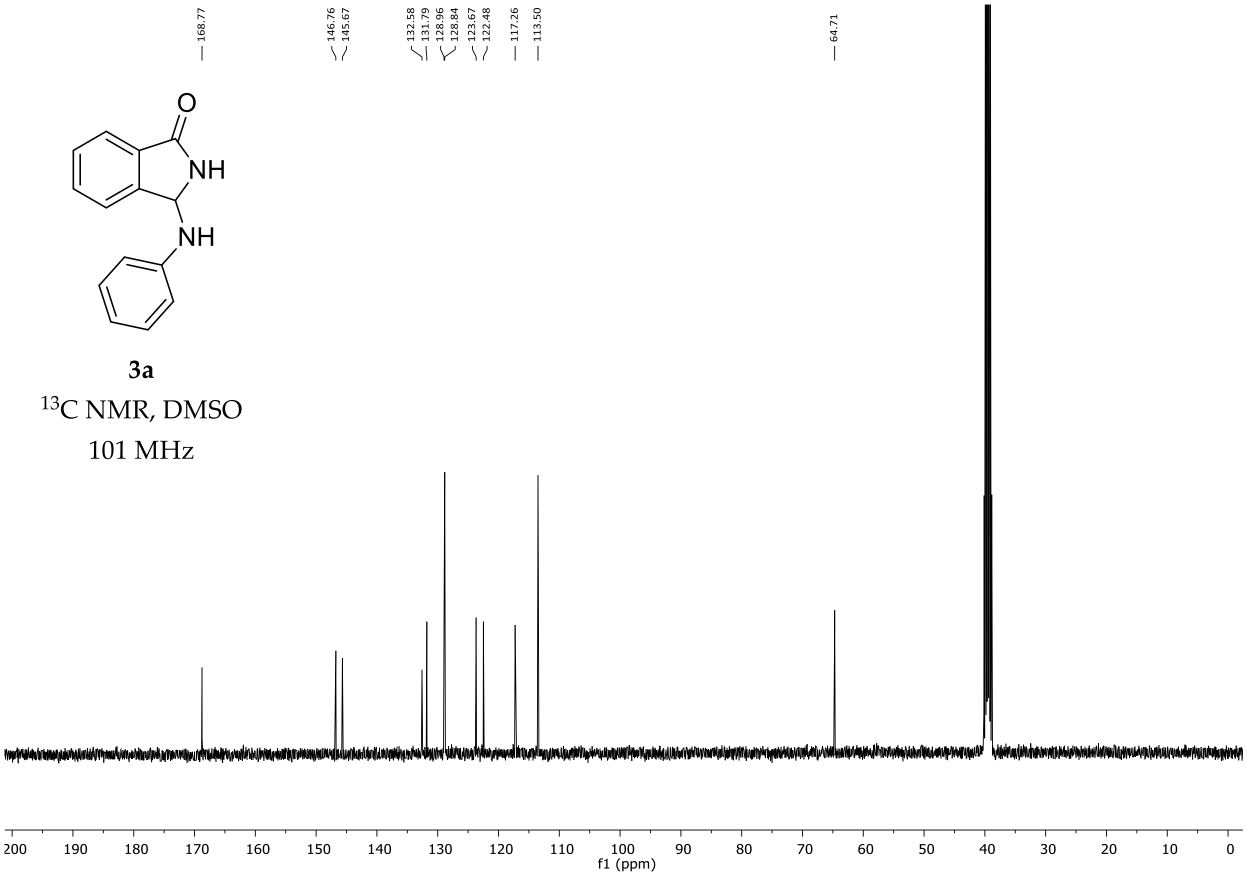

*3-((2-bromophenyl) amino) isoindolin-1-one (**3b**)*

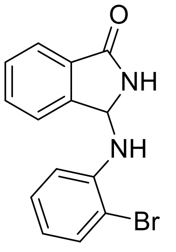

3b

^{1}H NMR, $CDCl_3$

400 MHz

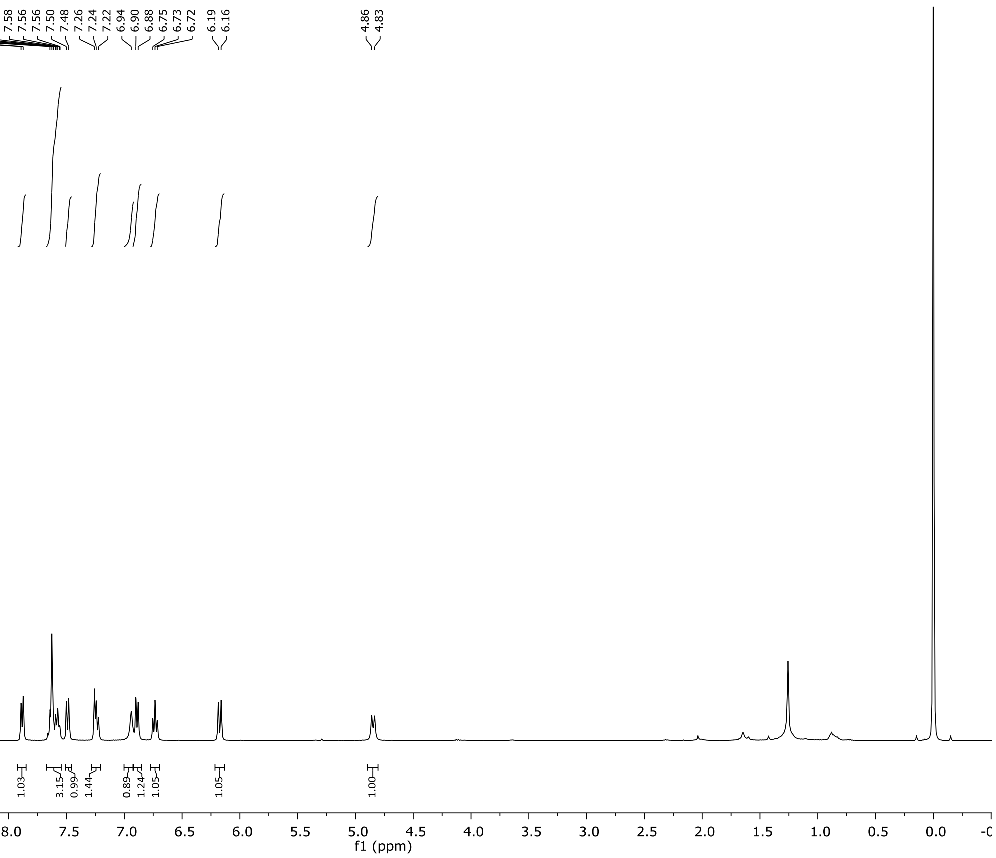

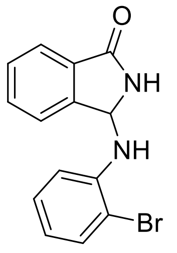

3b

^{13}C NMR, $CDCl_3$

101 MHz

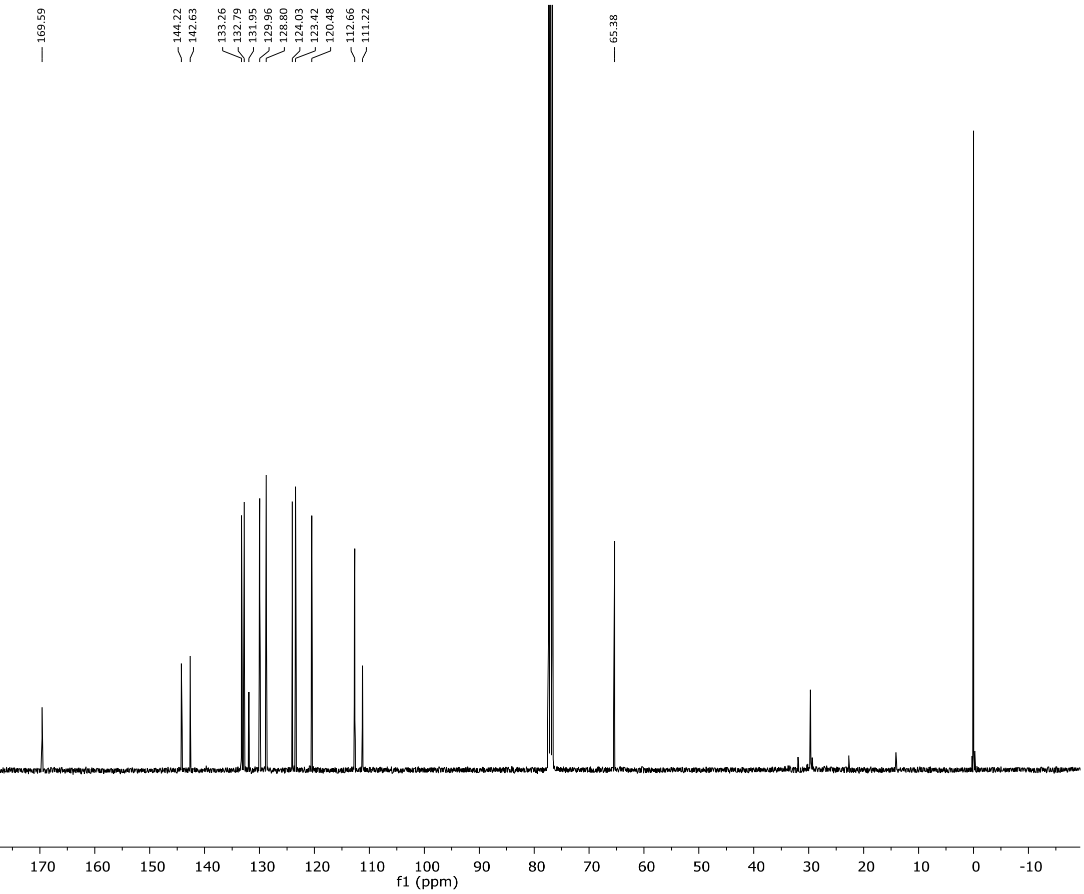

*3-((2-iodophenyl) amino) isoindolin-1-one (**3c**)*

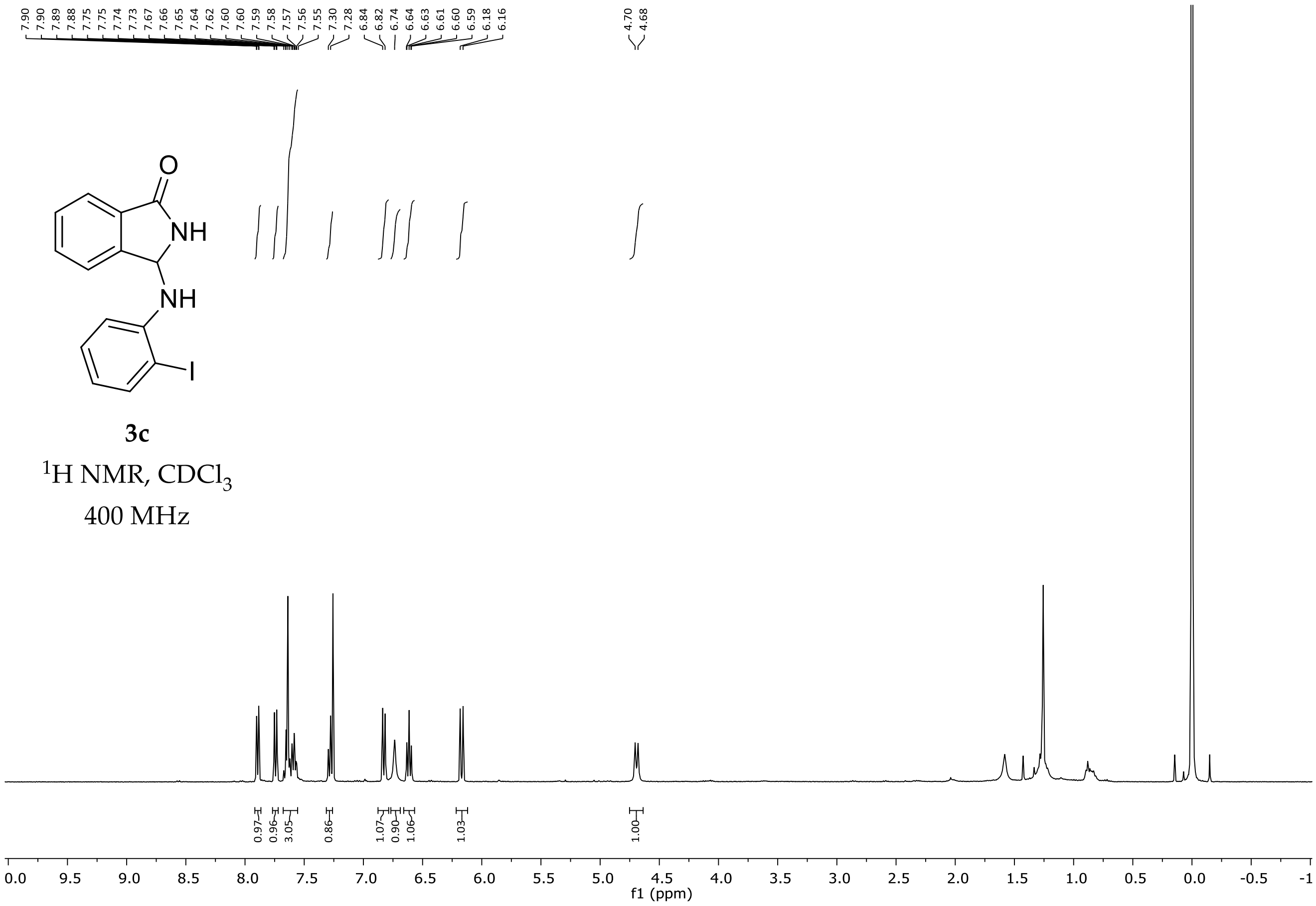

3c

^{1}H NMR, $CDCl_3$

400 MHz

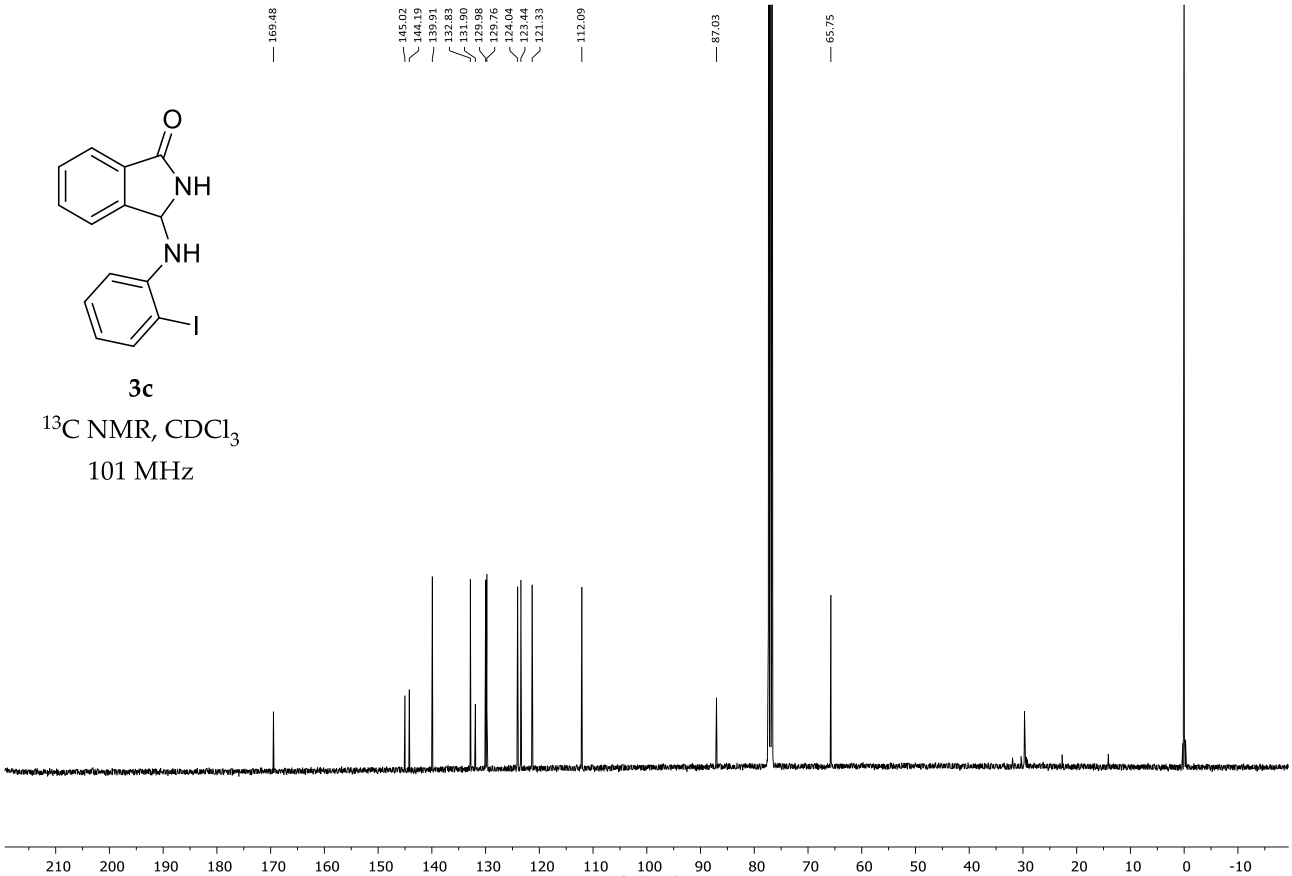

3c

^{13}C NMR, $CDCl_3$

101 MHz

*3-((2-chlorophenyl) amino) isoindolin-1-one (**3d**)*

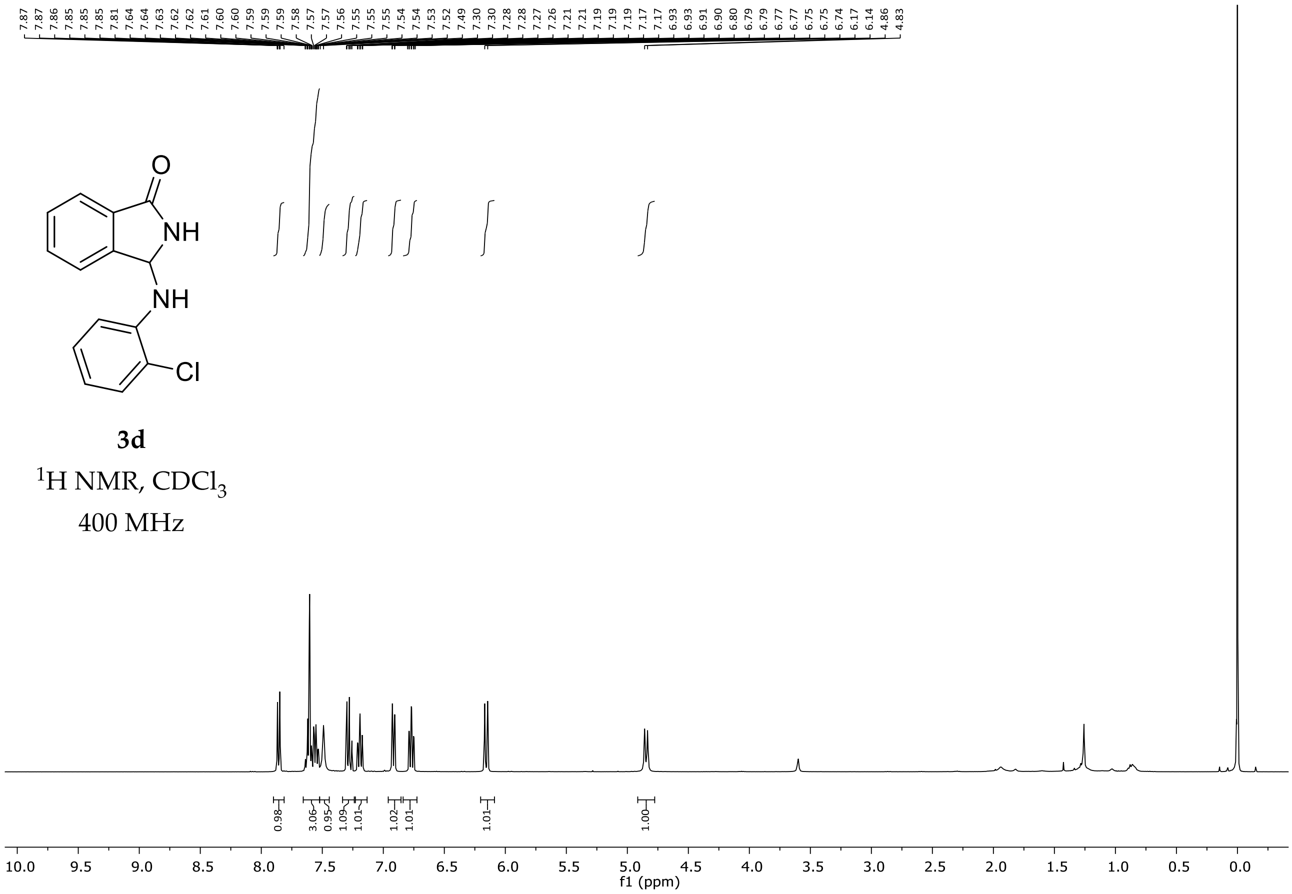

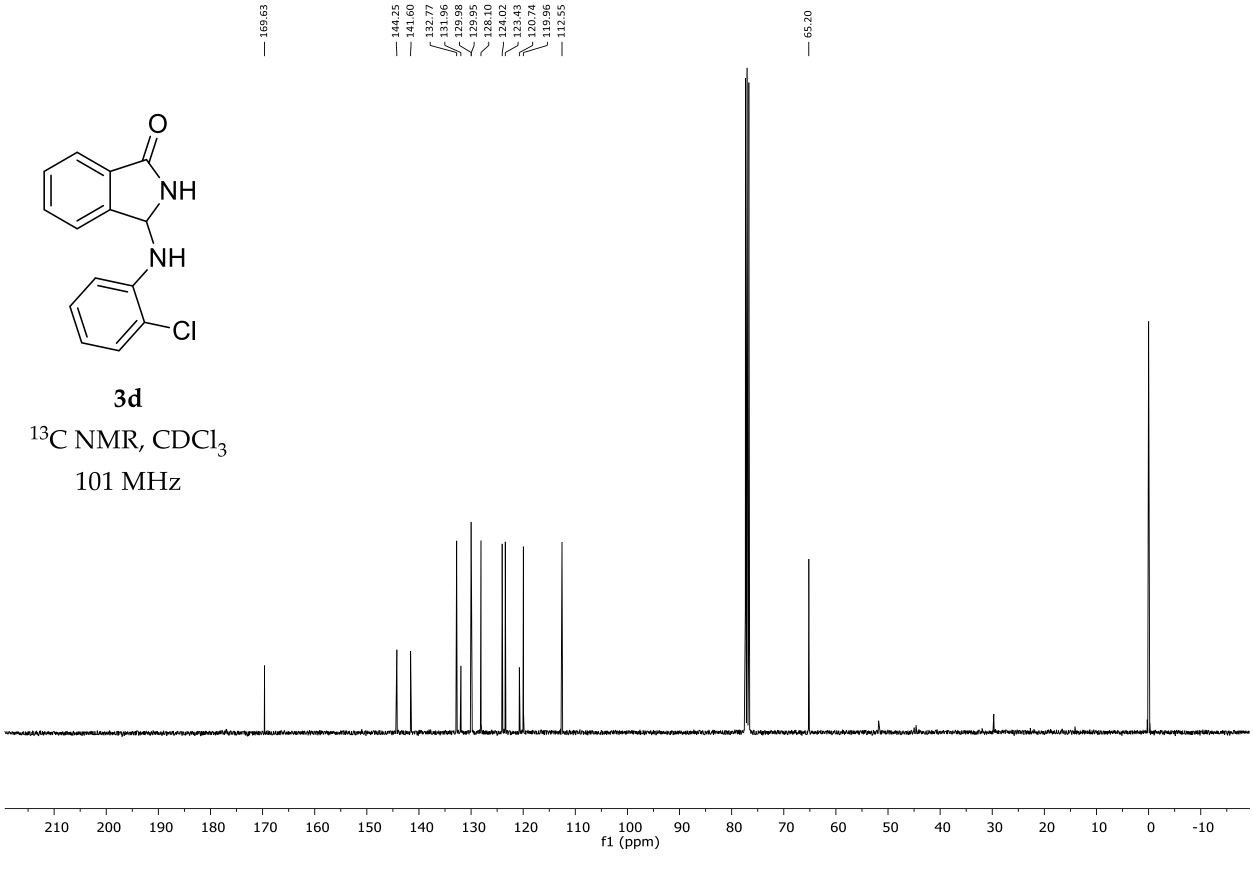

*3-((2-methoxyphenyl) amino) isoindolin-1-one (**3e**)*

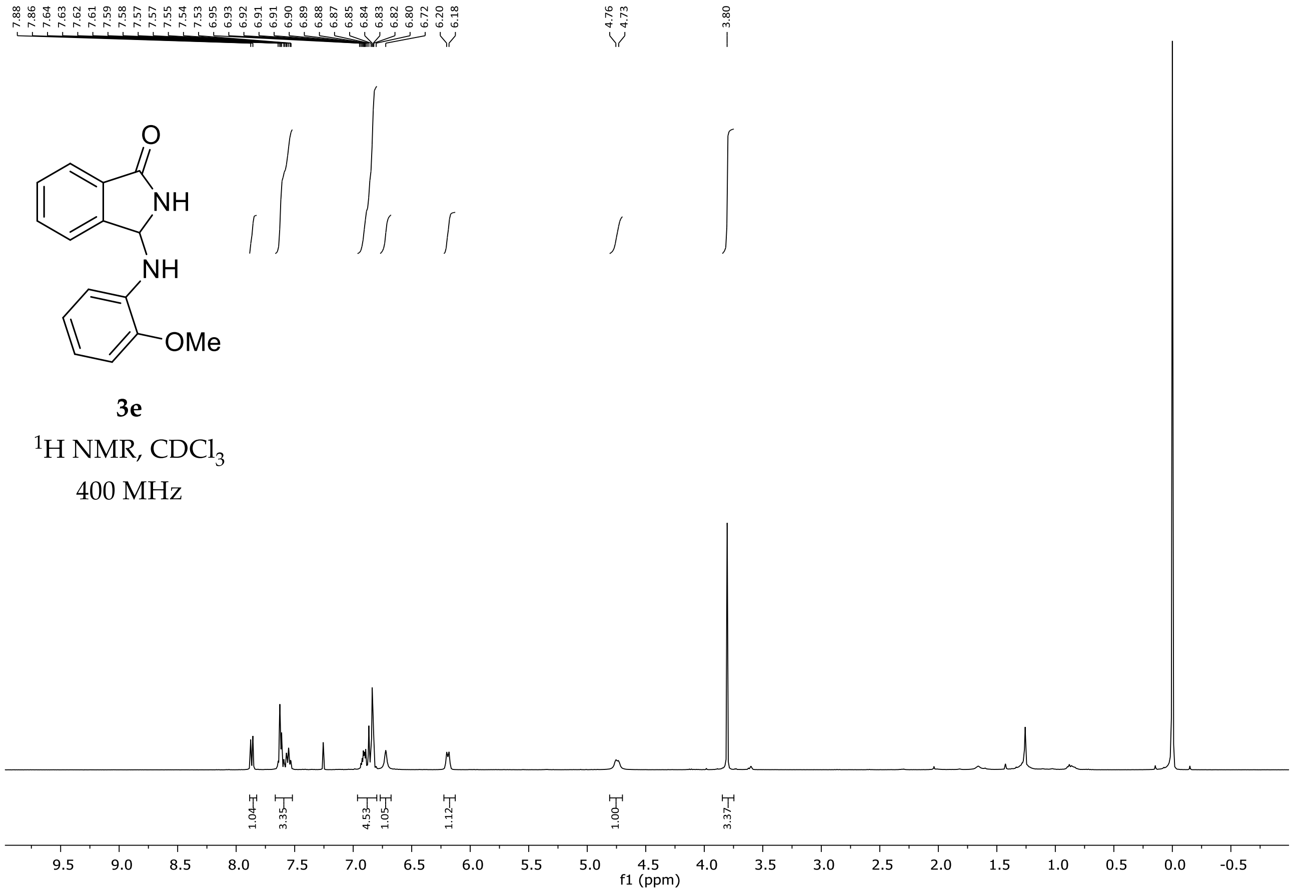

3e
^{1}H NMR, $CDCl_3$
400 MHz

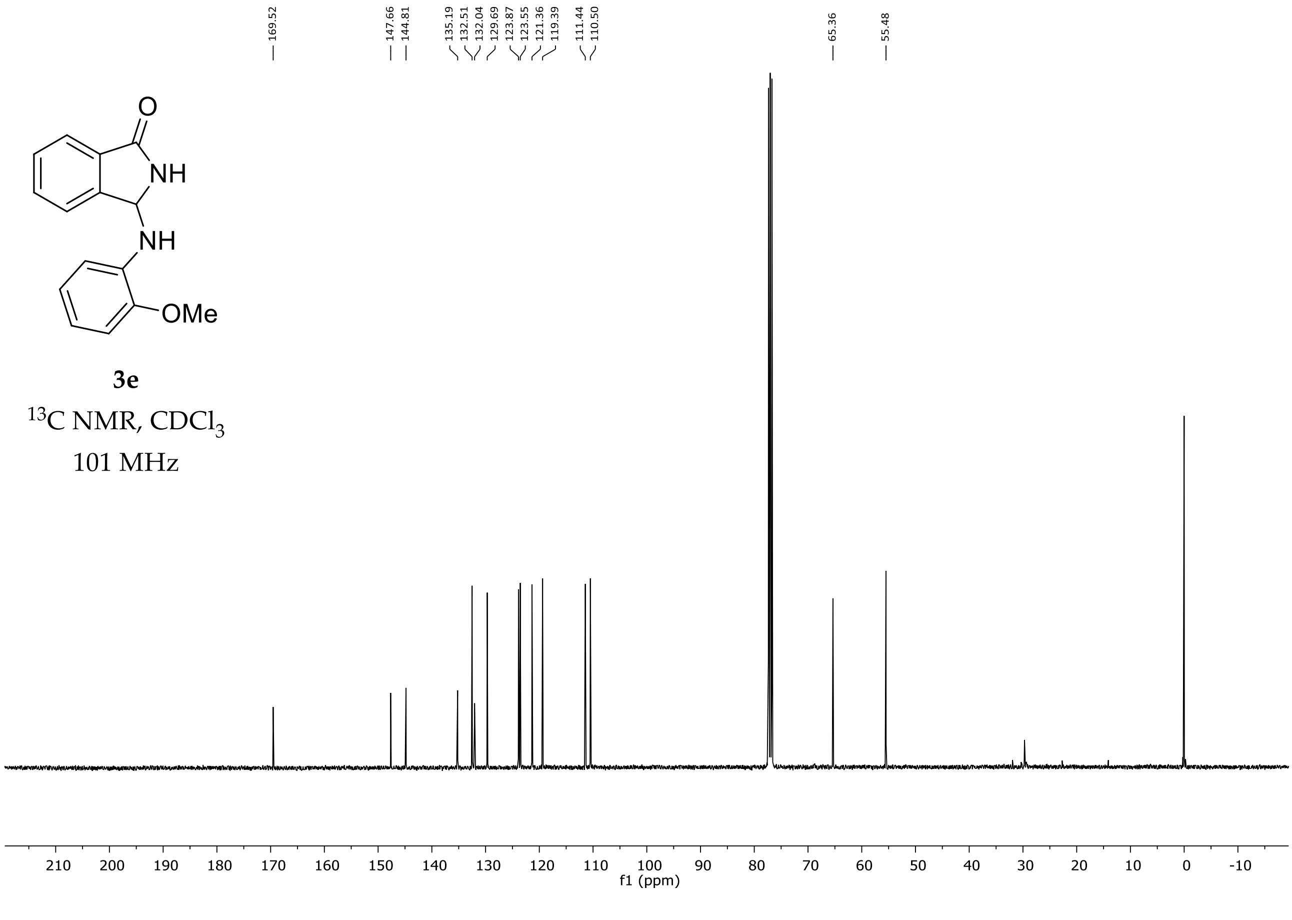

3e
^{13}C NMR, $CDCl_3$
101 MHz

*2-((3-oxoisoindolin-1-yl) amino) benzonitrile (**3f**)*

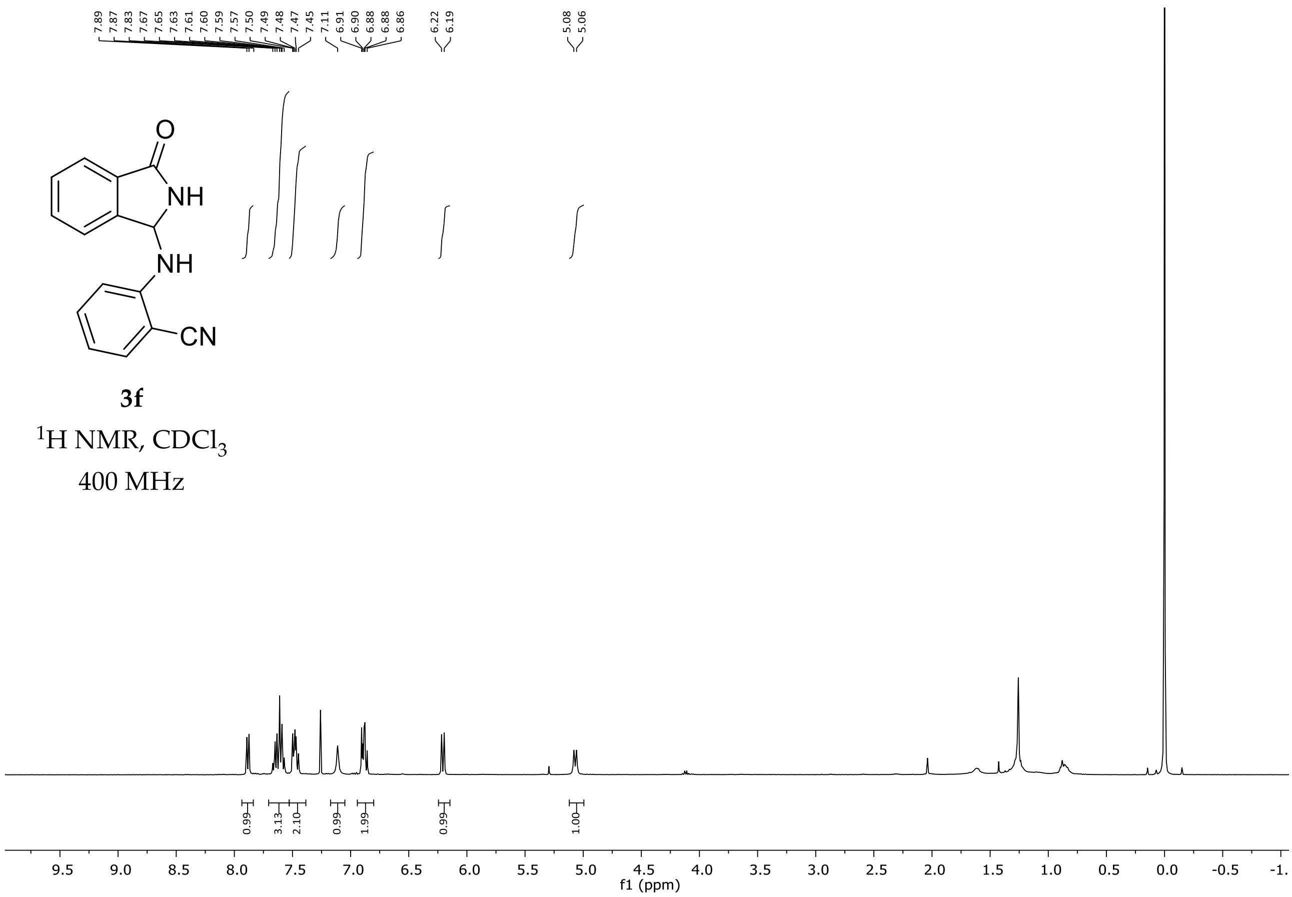

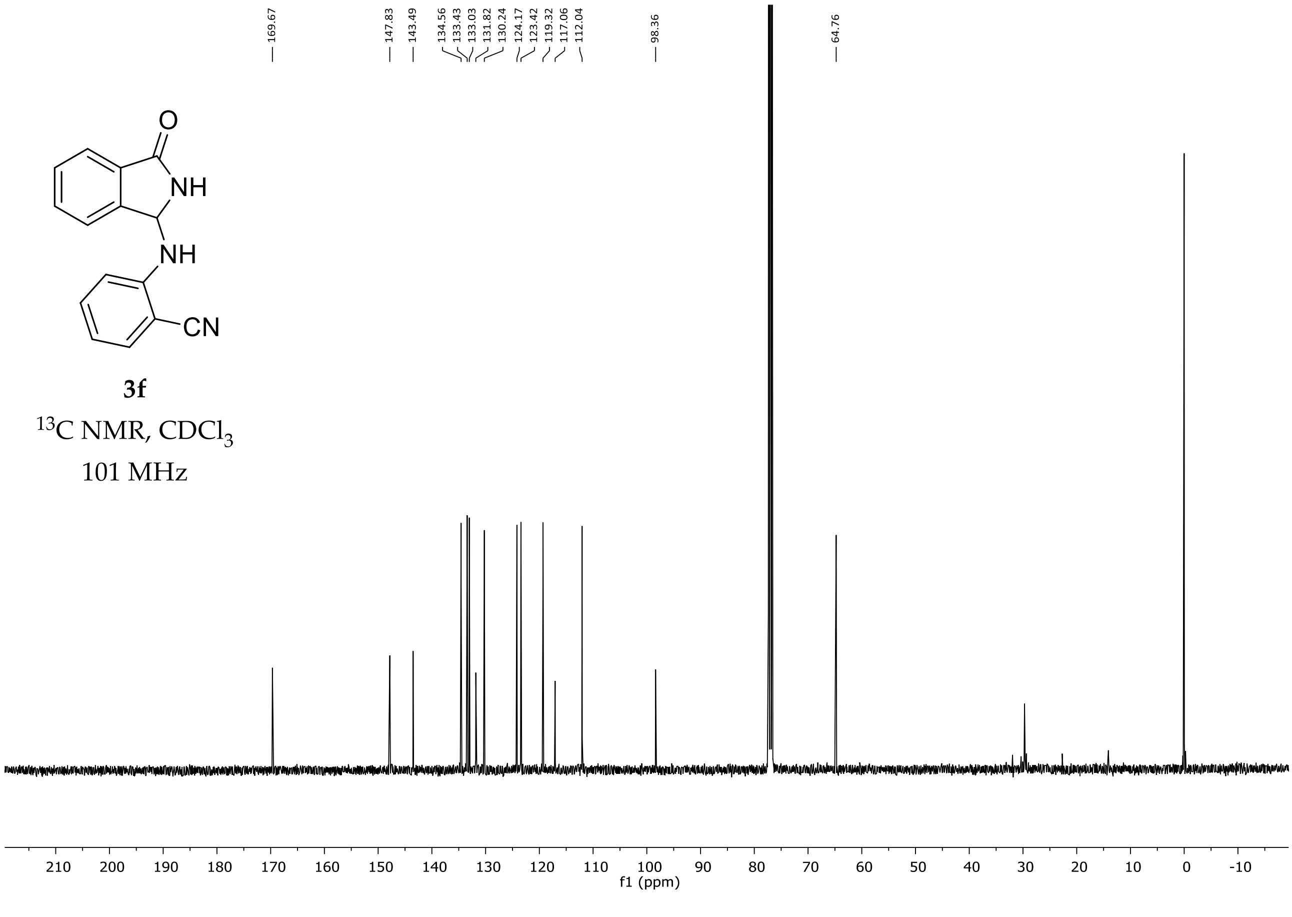

*2-((3-oxoisoindolin-1-yl) amino) benzamide (**3g**)*

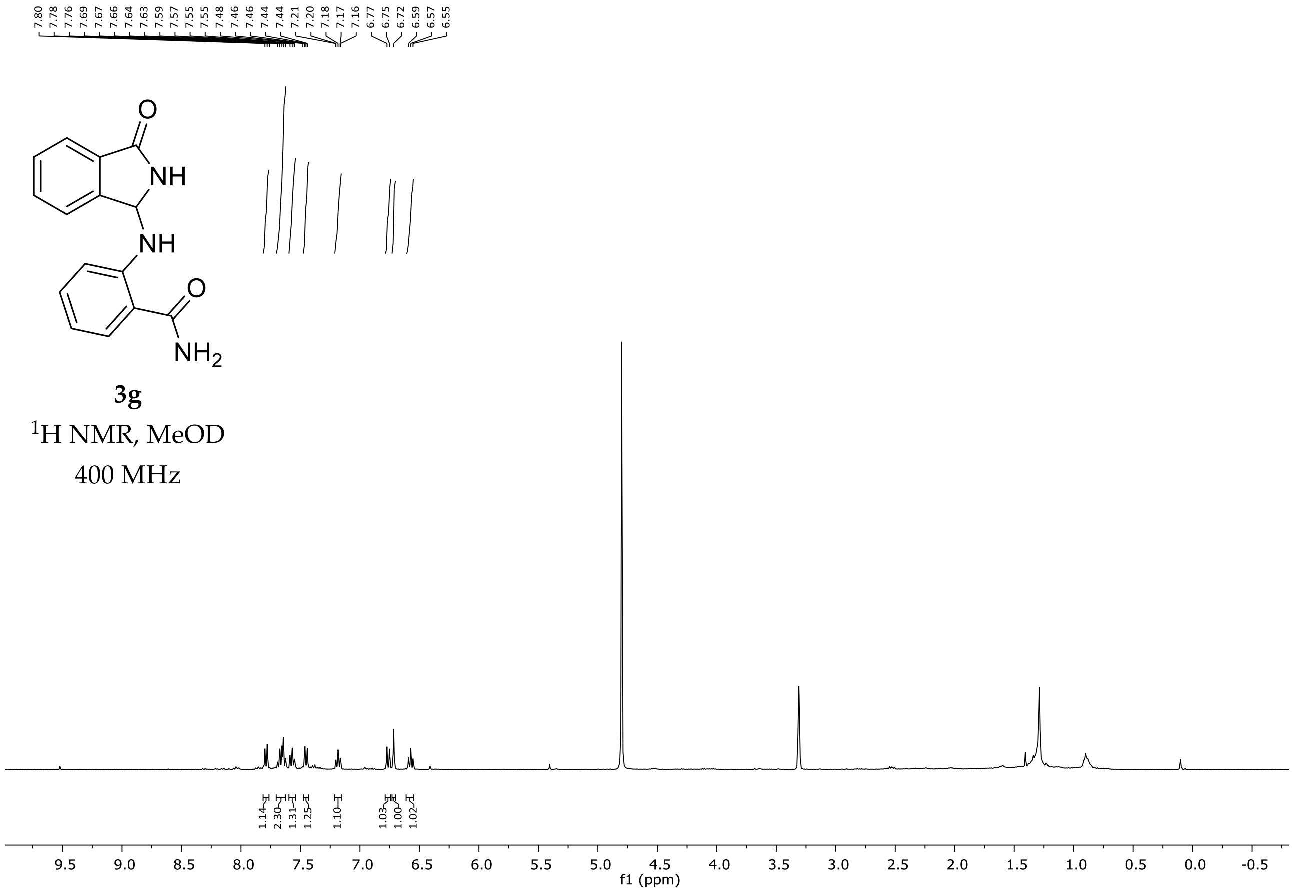

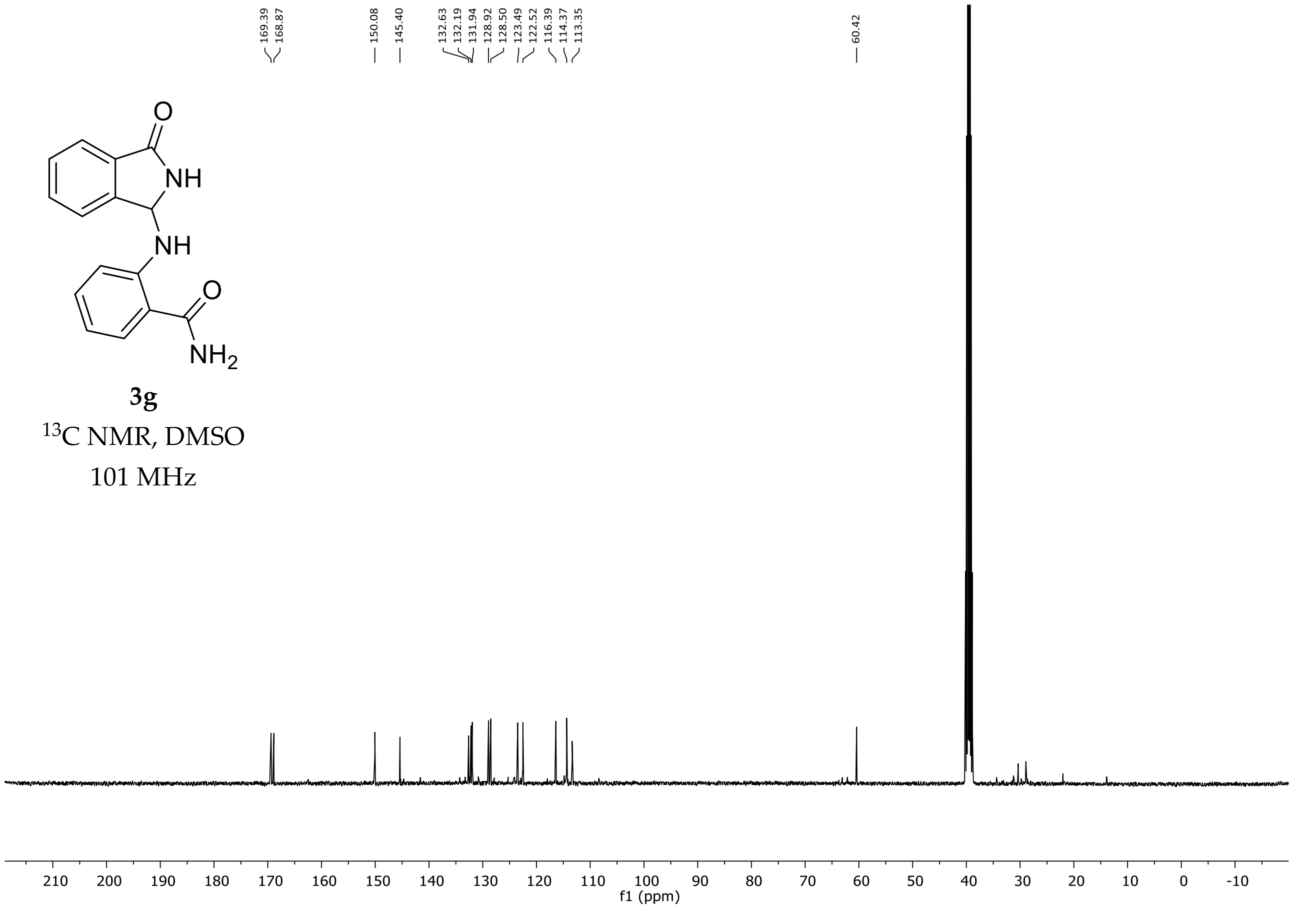

*3-((2-benzoylphenyl) amino) isoindolin-1-one (**3h**)*

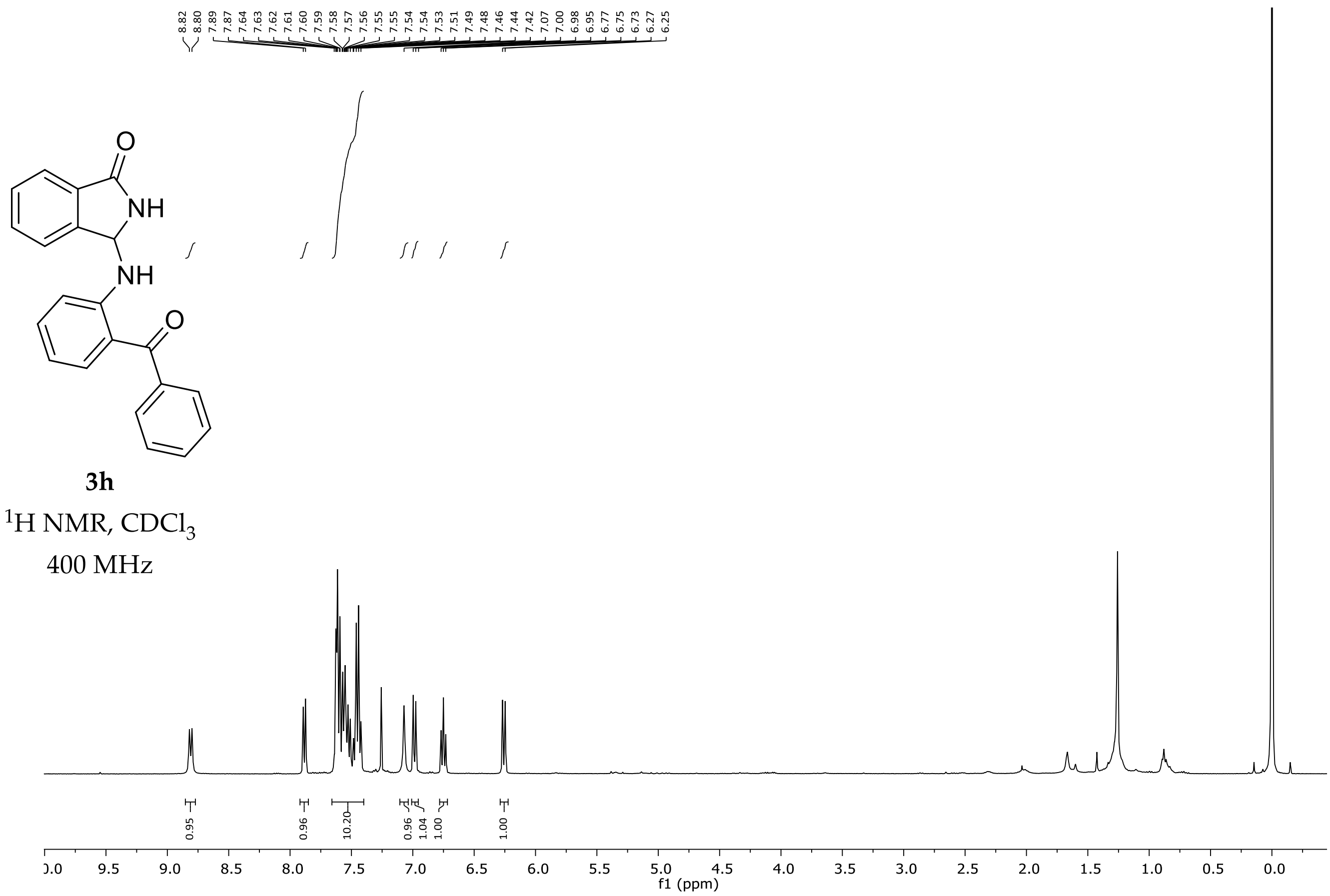

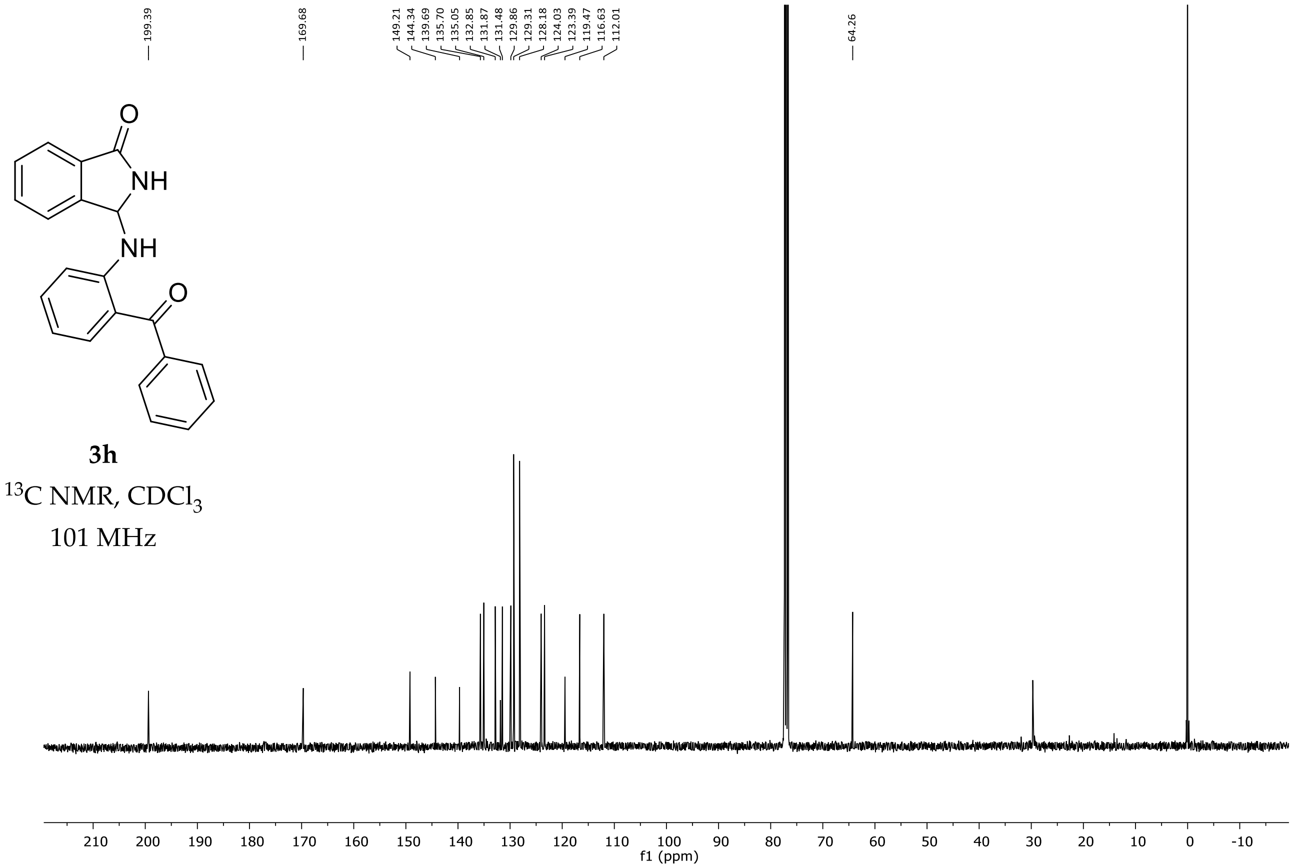

*3-((2-acetylphenyl) amino) isoindolin-1-one (**3i**)*

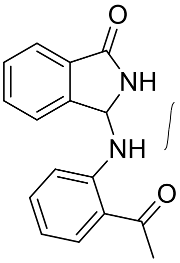

3i

^{1}H NMR, $CDCl_3$

400 MHz

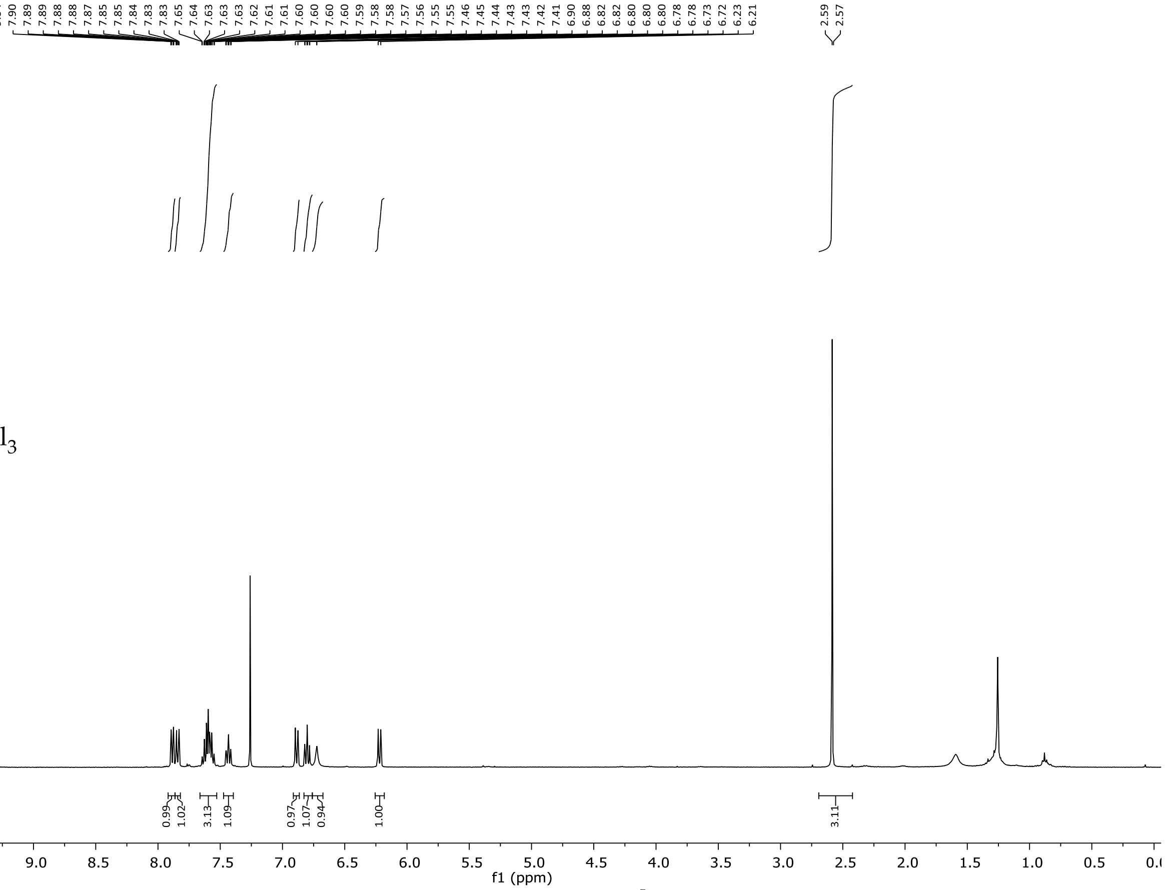

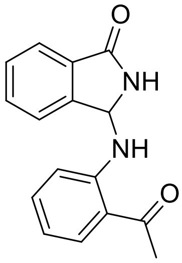

3i

^{13}C NMR, $CDCl_3$

101 MHz

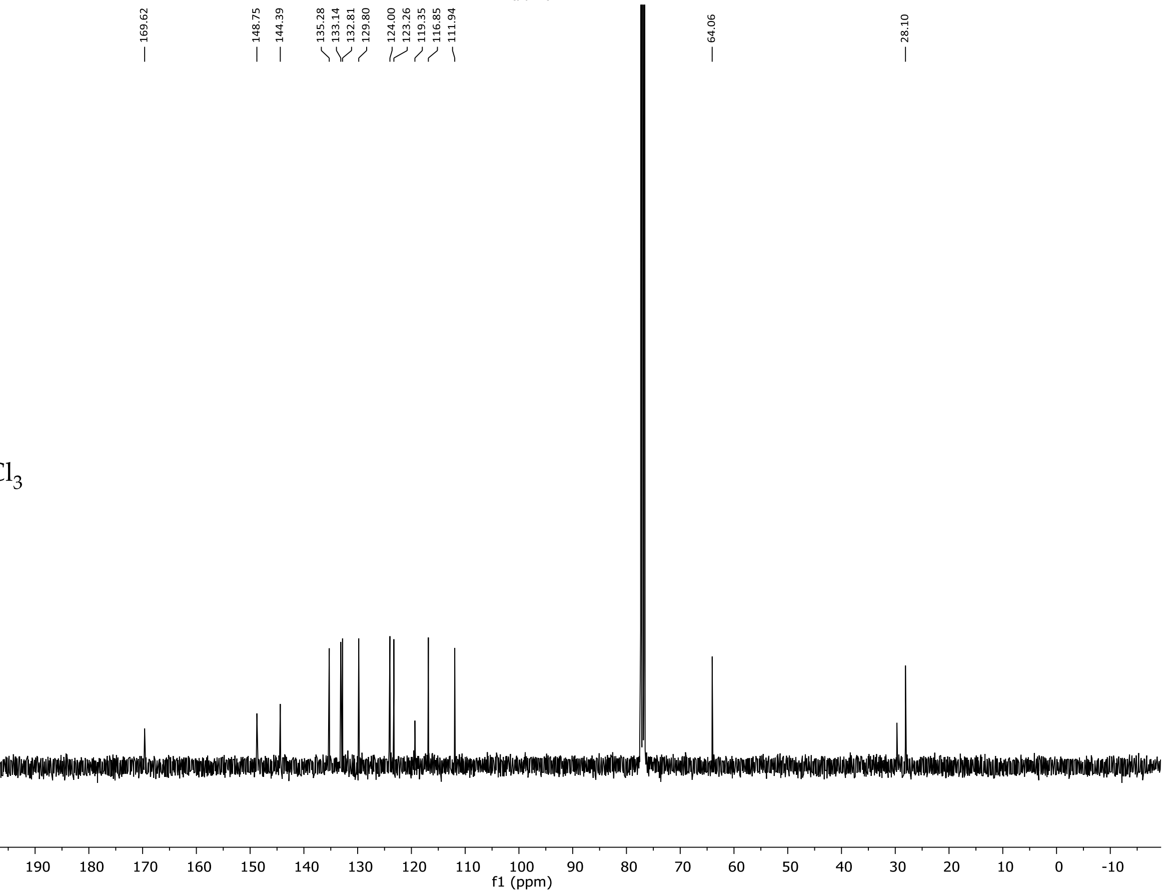

*3-((4-chlorophenyl) amino) isoindolin-1-one (**3j**)*

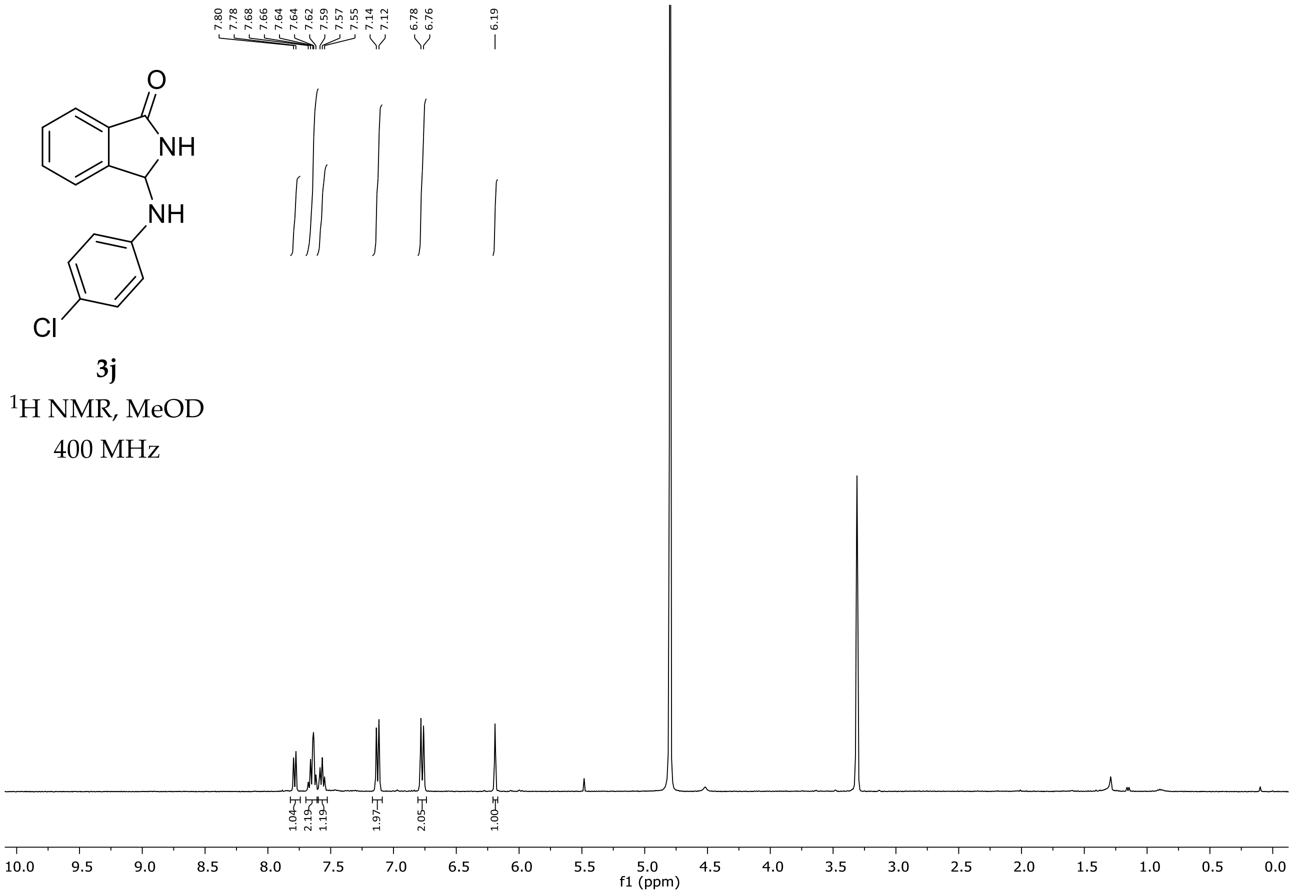

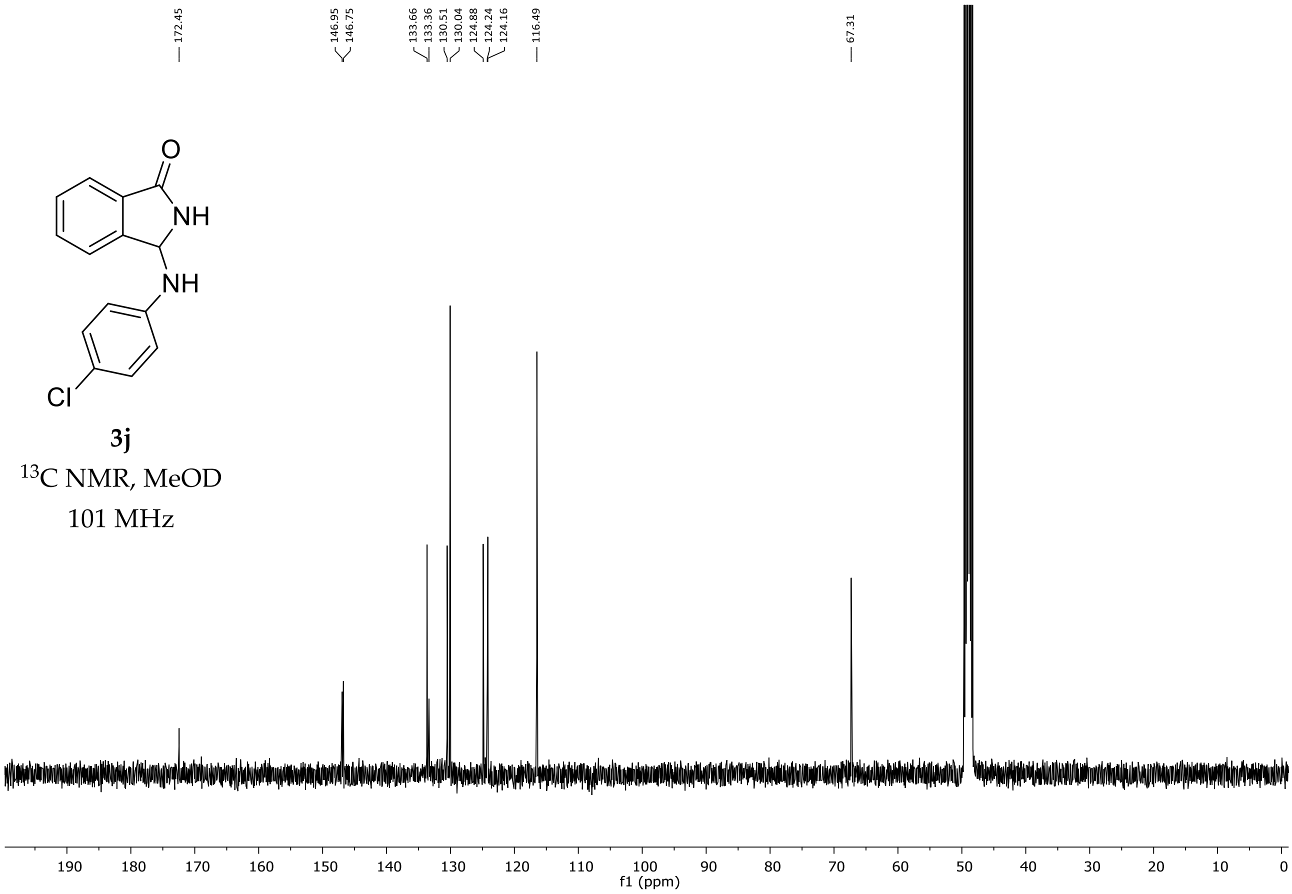

*3-((4-fluorophenyl) amino) isoindolin-1-one (**3k**)*

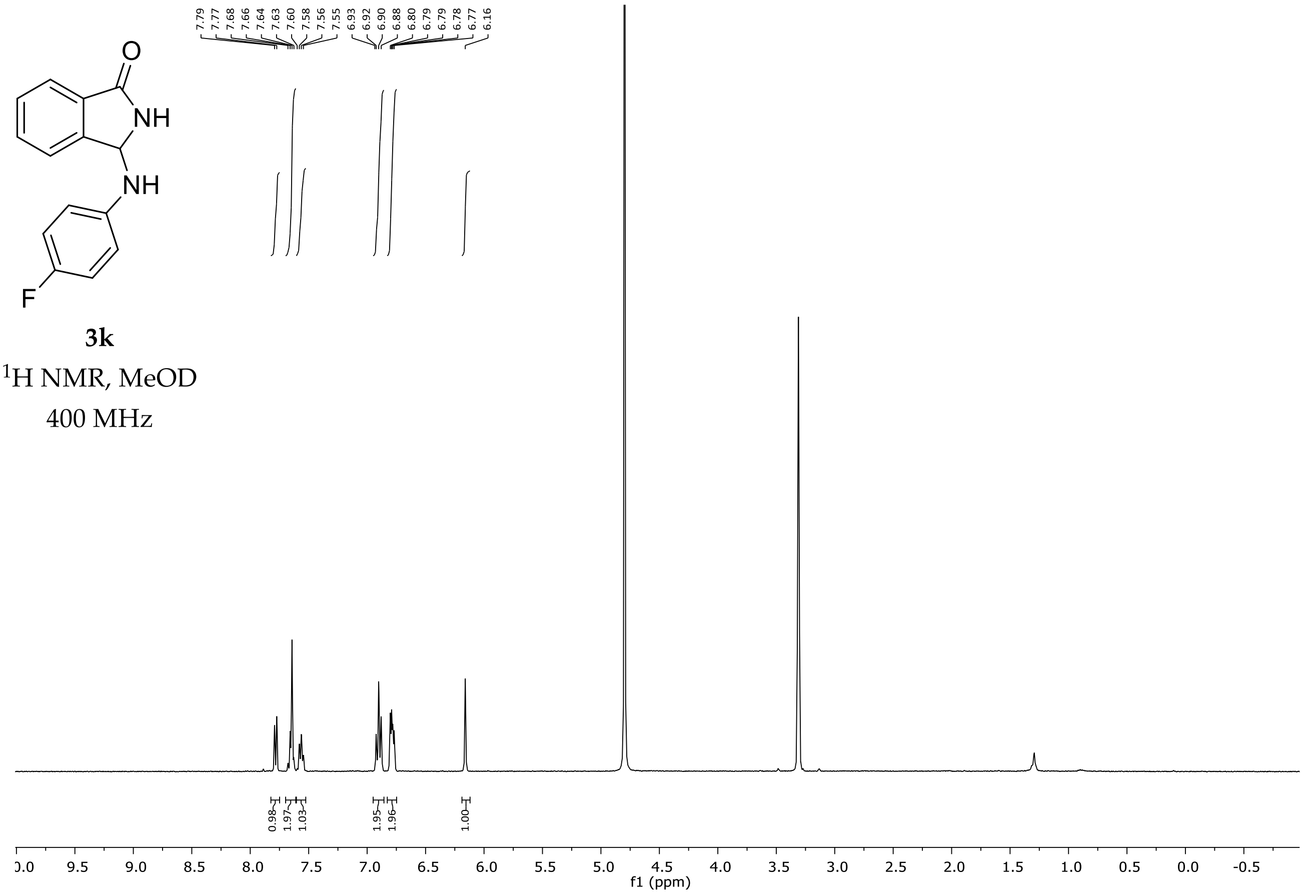

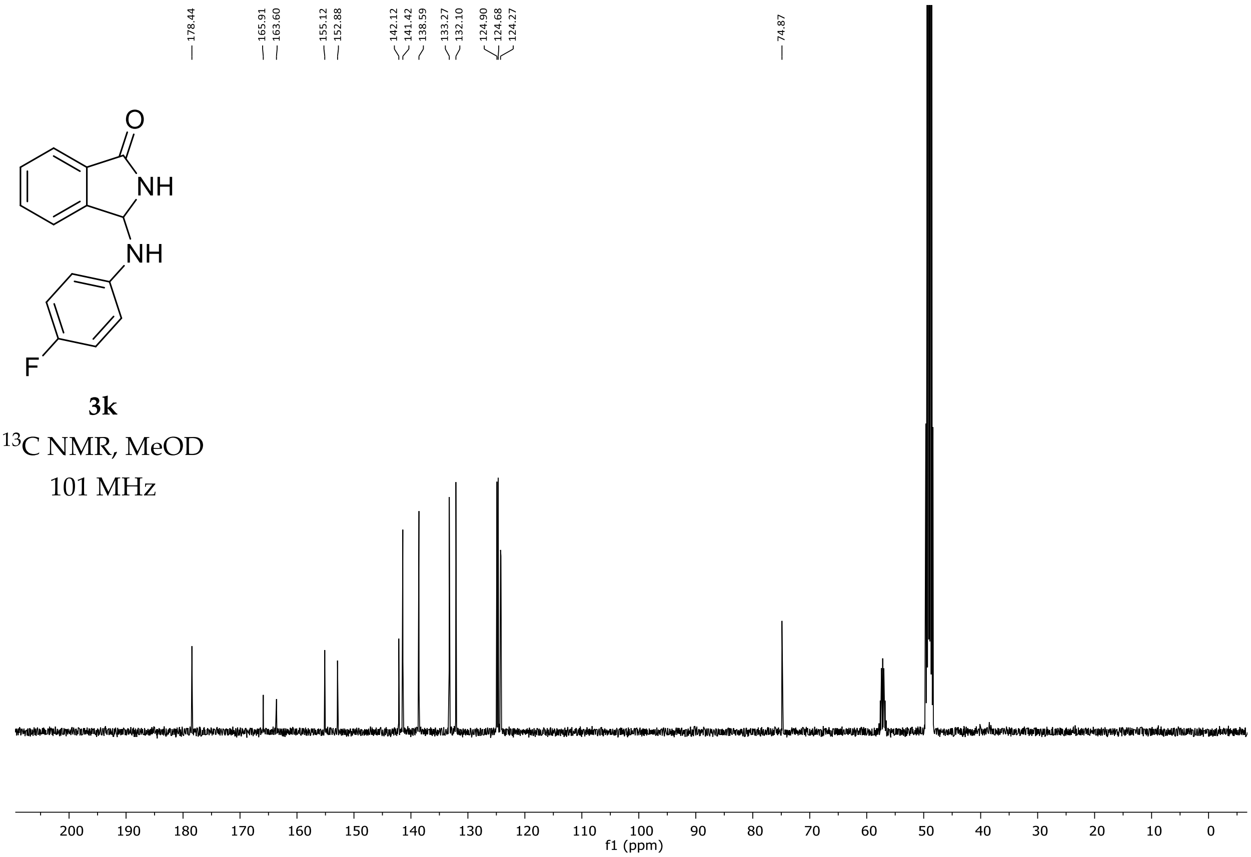

*3-(p-tolylamino) isoindolin-1-one (**3l**)*

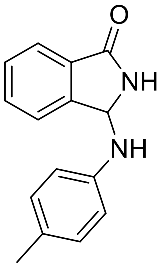

3l

^{1}H NMR, $CDCl_3$

400 MHz

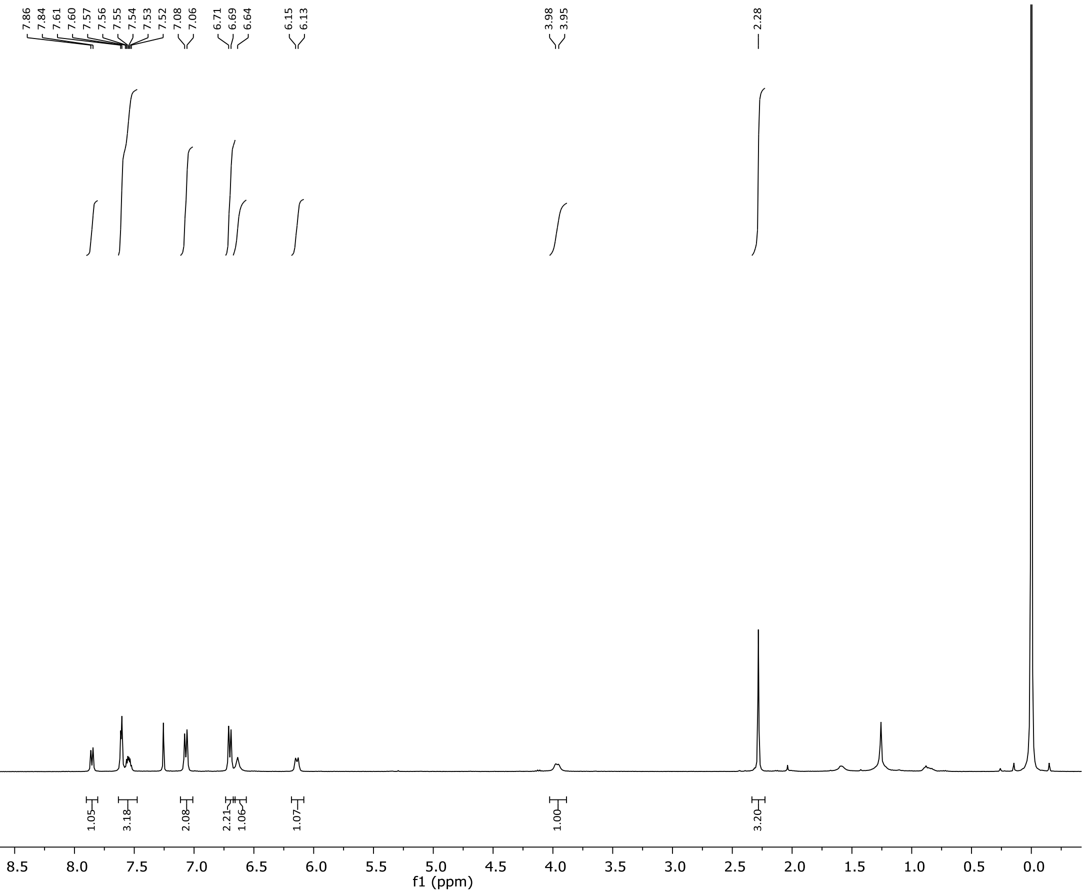

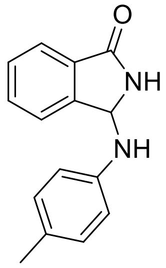

3l

^{13}C NMR, $CDCl_3$

101 MHz

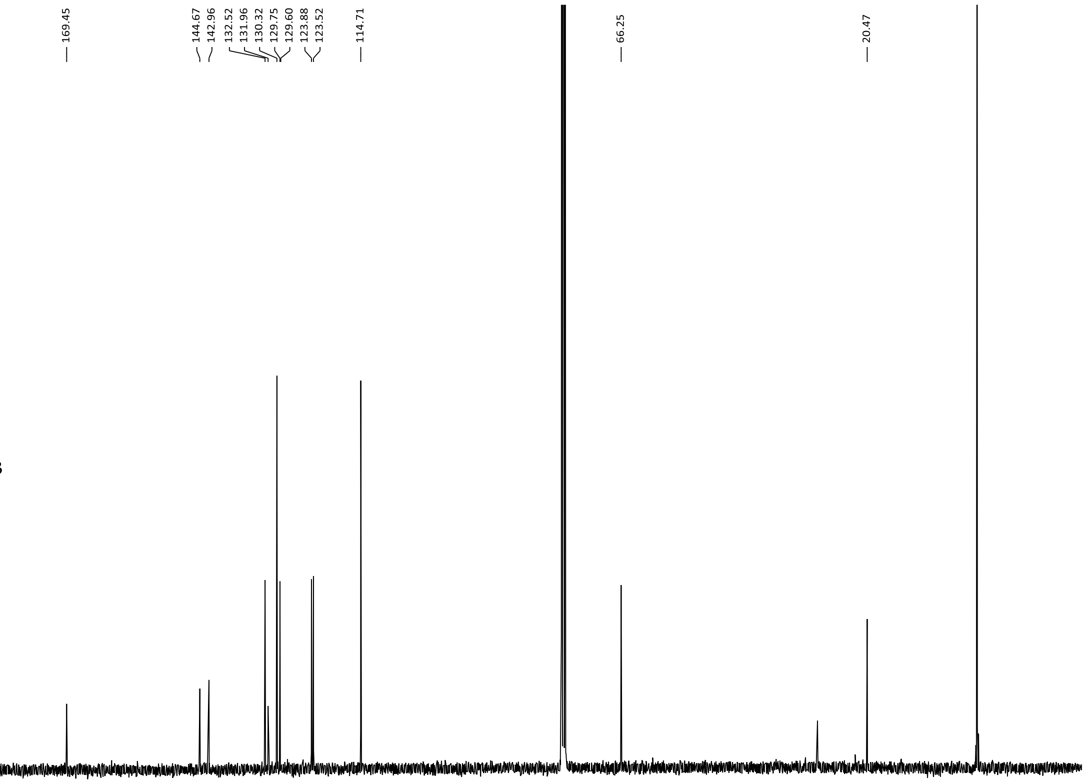

*3-((4-methoxyphenyl) amino) isoindolin-1-one (**3m**)*

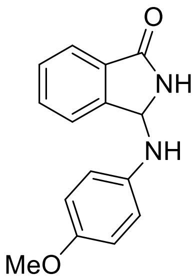

3m

^{1}H NMR, $CDCl_3$

400 MHz

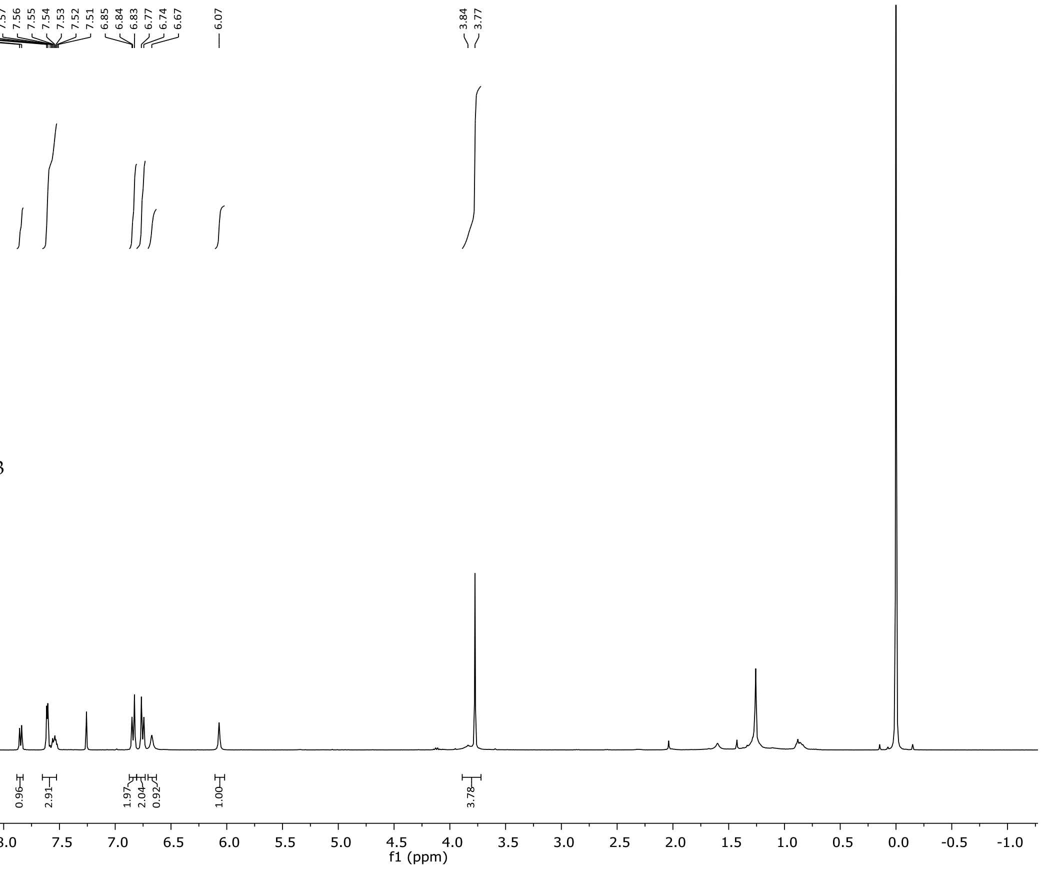

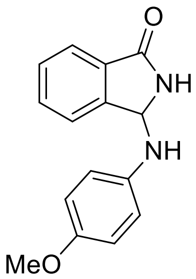

3m

^{13}C NMR, $CDCl_3$

101 MHz

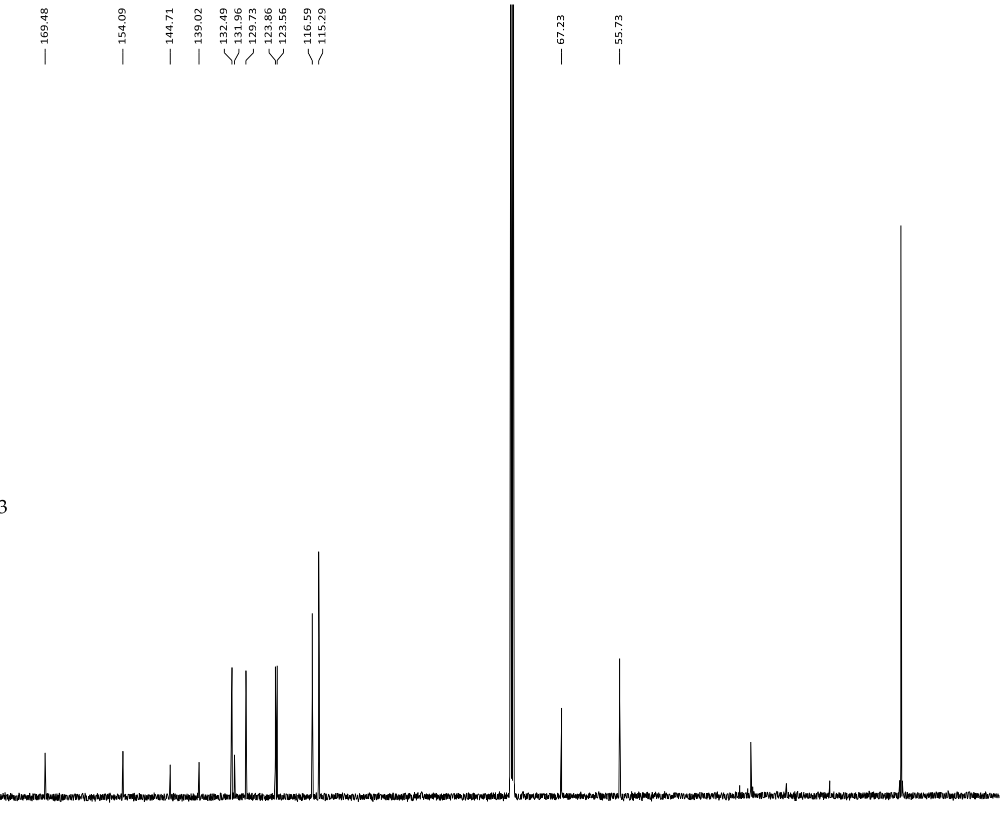

*3-((4-ethoxyphenyl) amino) isoindolin-1-one (**3n**)*

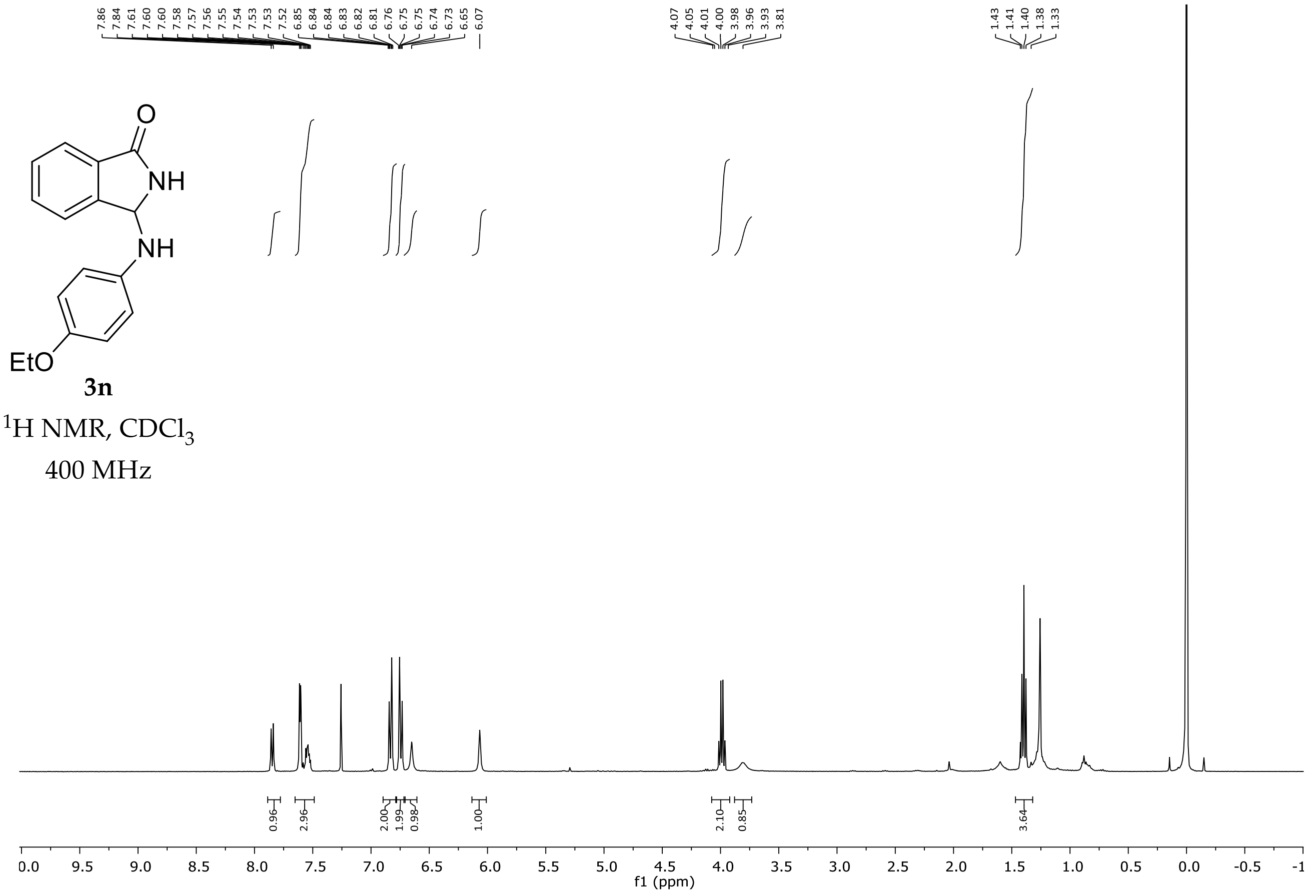

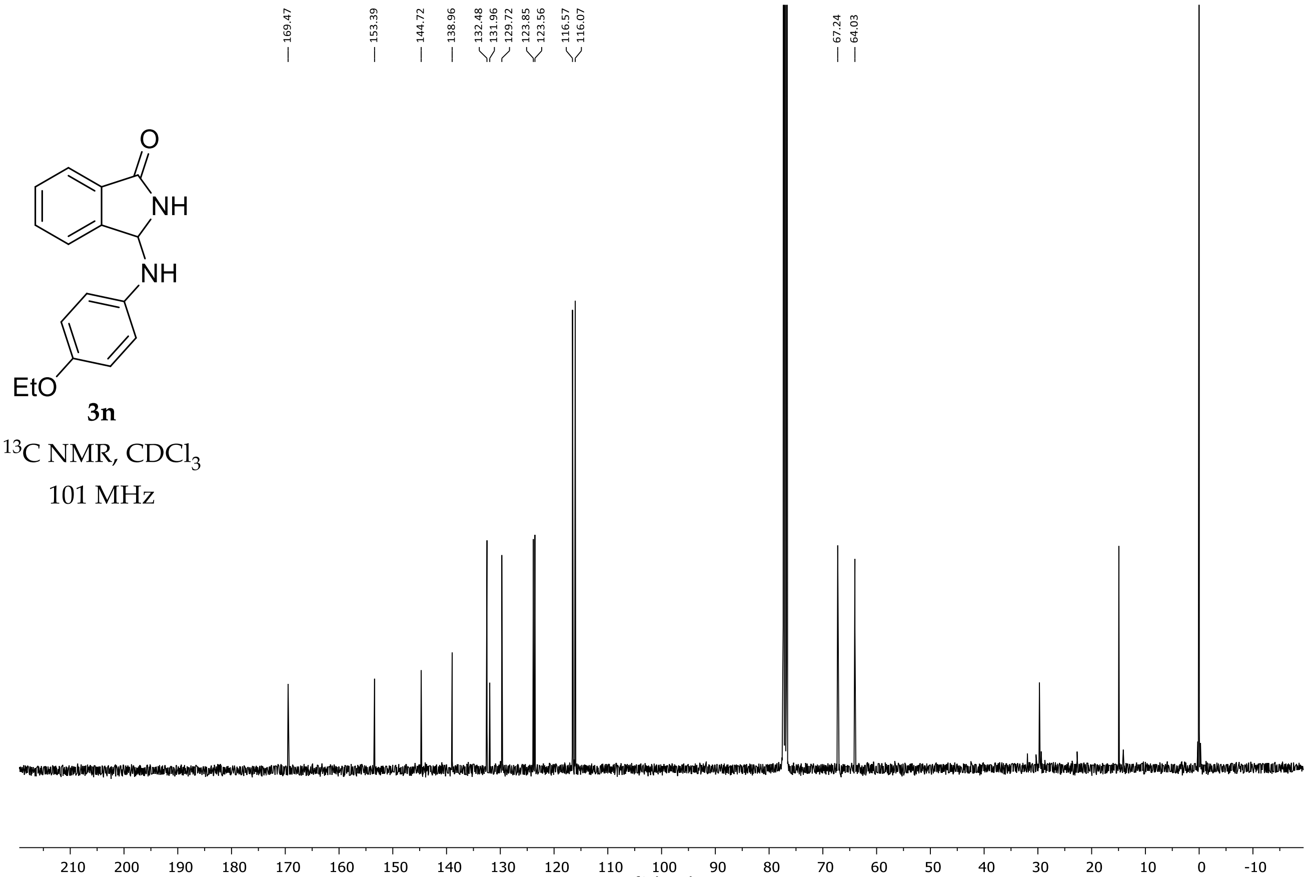

*3-(pyridin-2-ylamino) isoindolin-1-one (**3p**)*

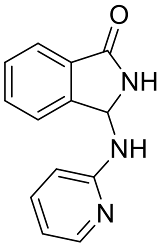

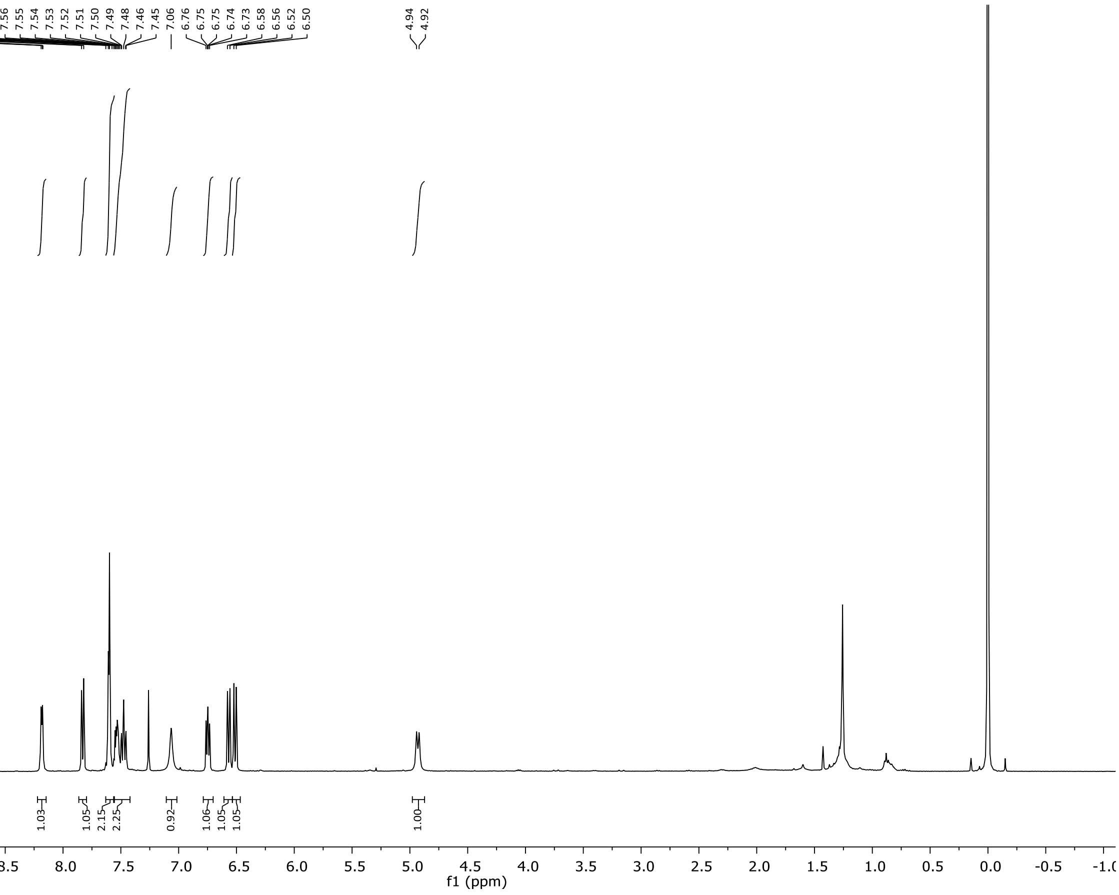

3p

^{1}H NMR, $CDCl_3$

400 MHz

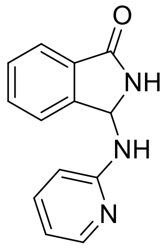

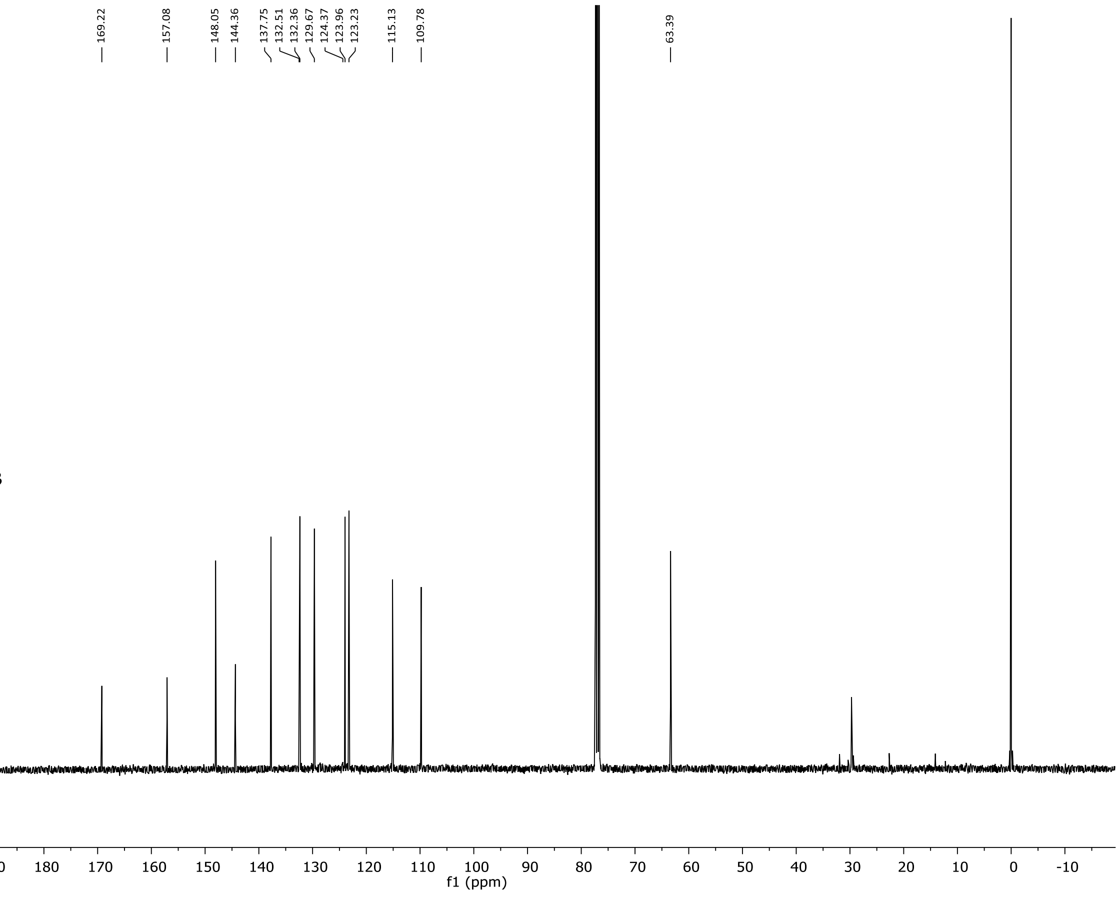

3p

^{13}C NMR, $CDCl_3$

101 MHz

*3-((2-bromo-4-methylphenyl) amino) isoindolin-1-one (**3q**)*

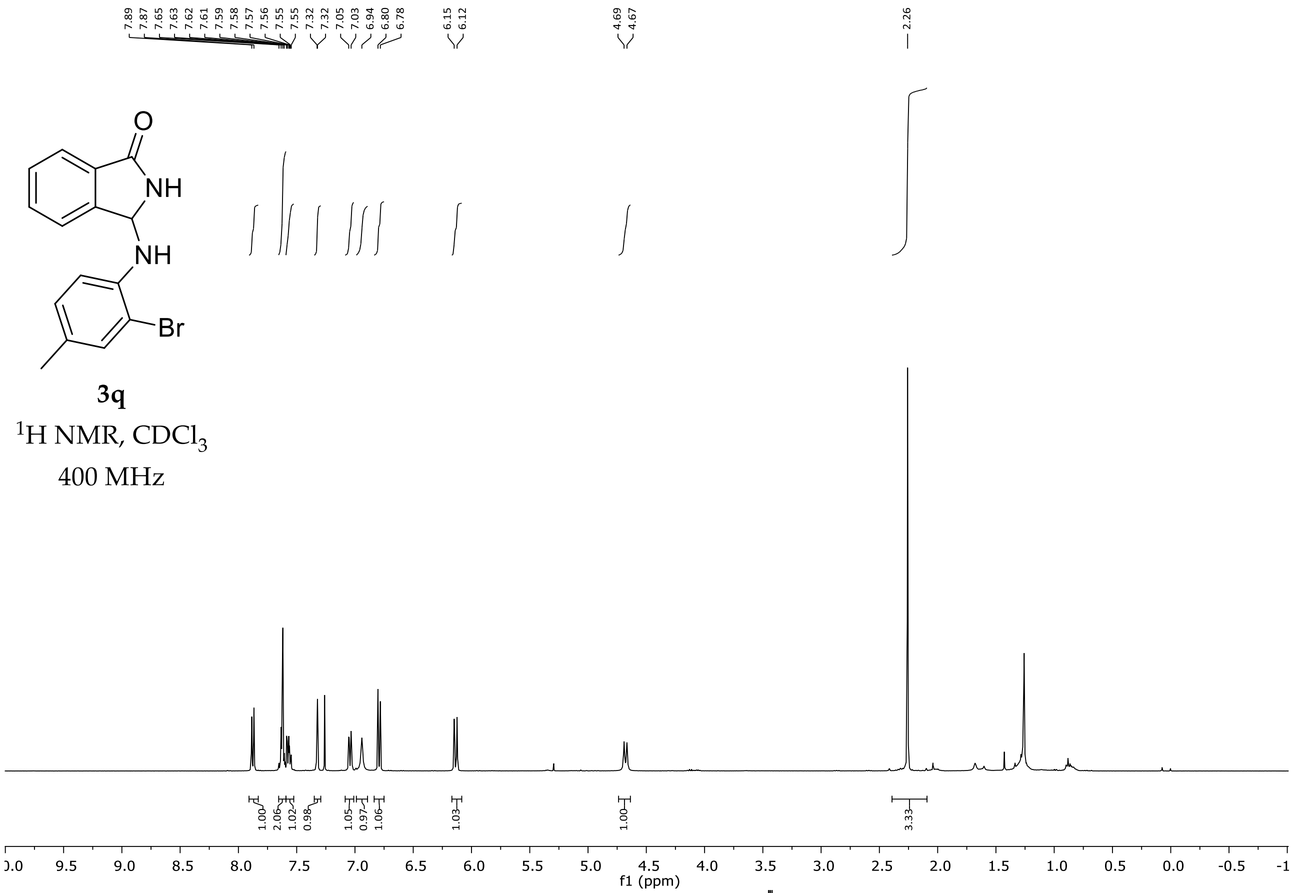

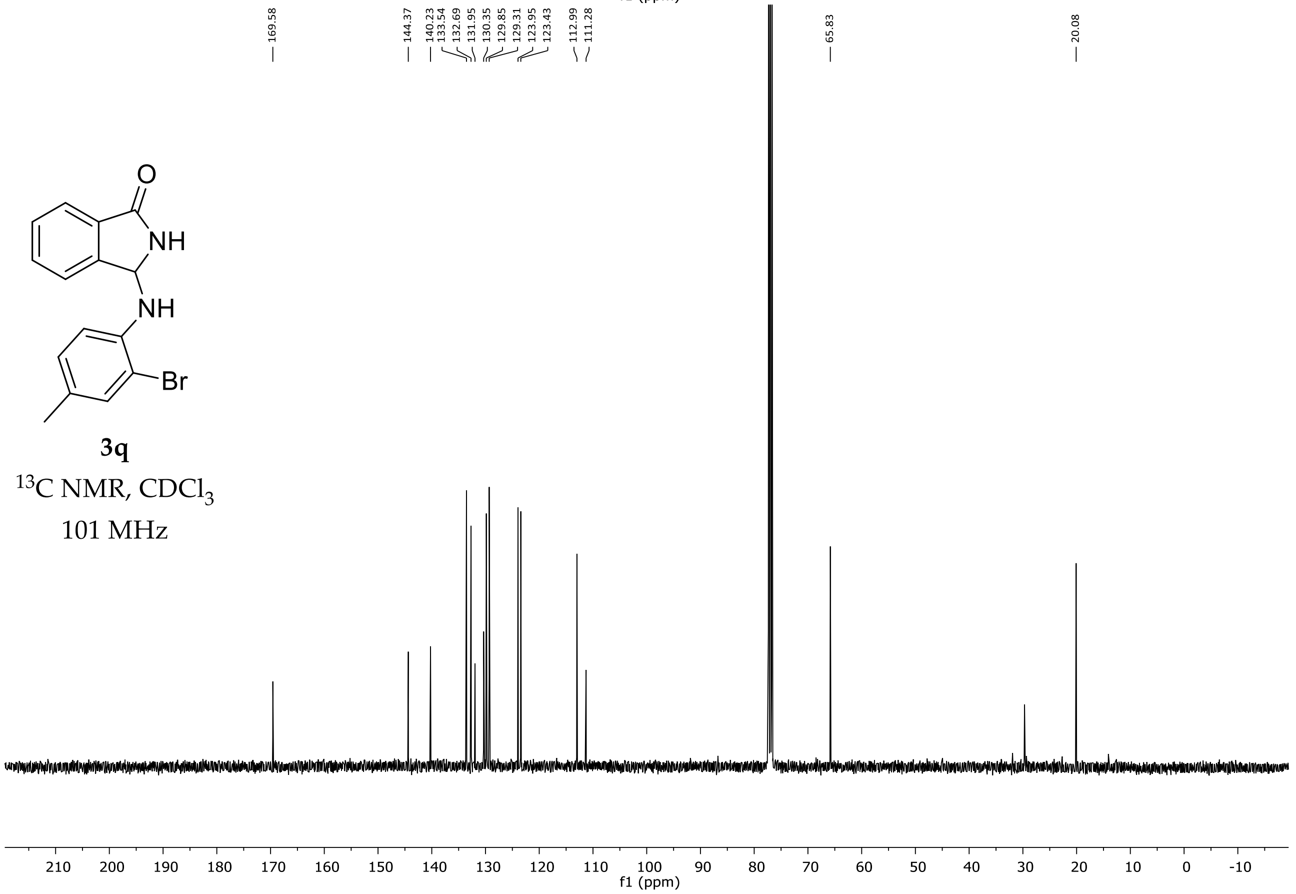

*3-((2,4-dimethylphenyl) amino) isoindolin-1-one (**3r**)*

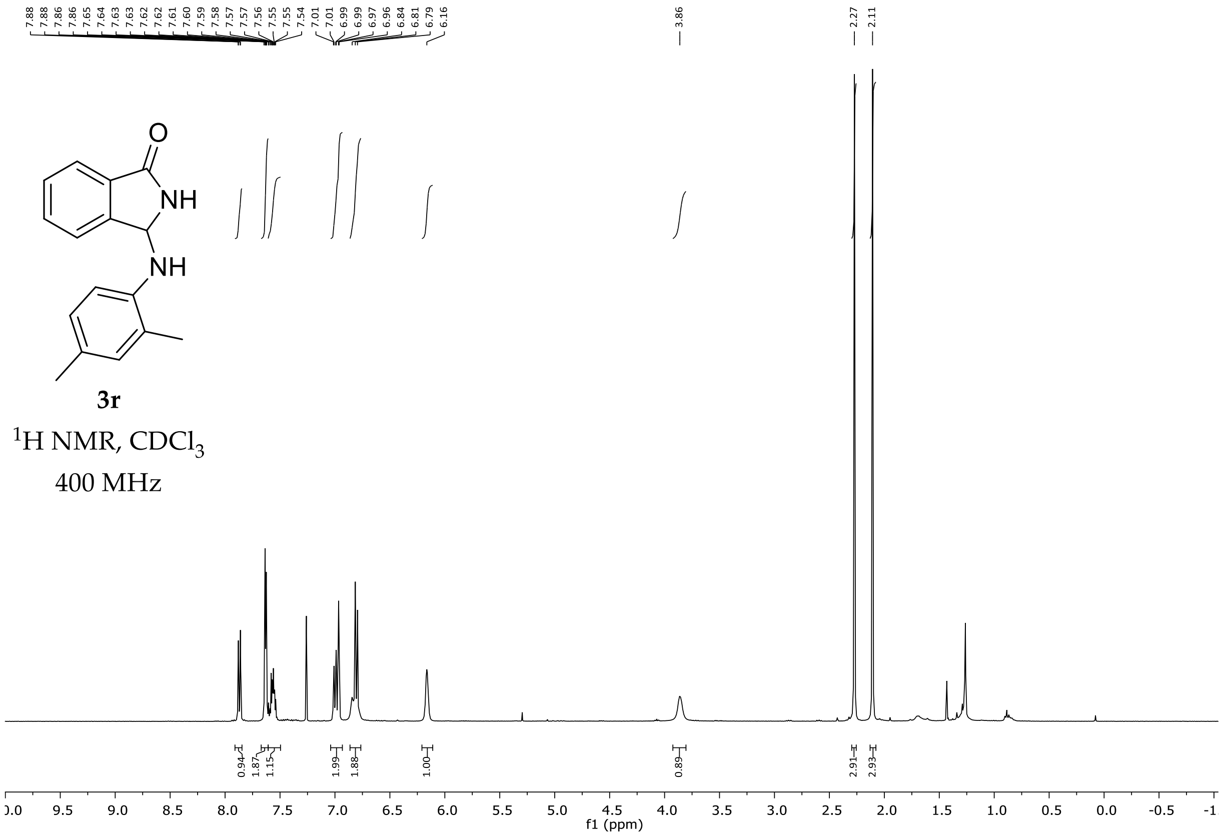

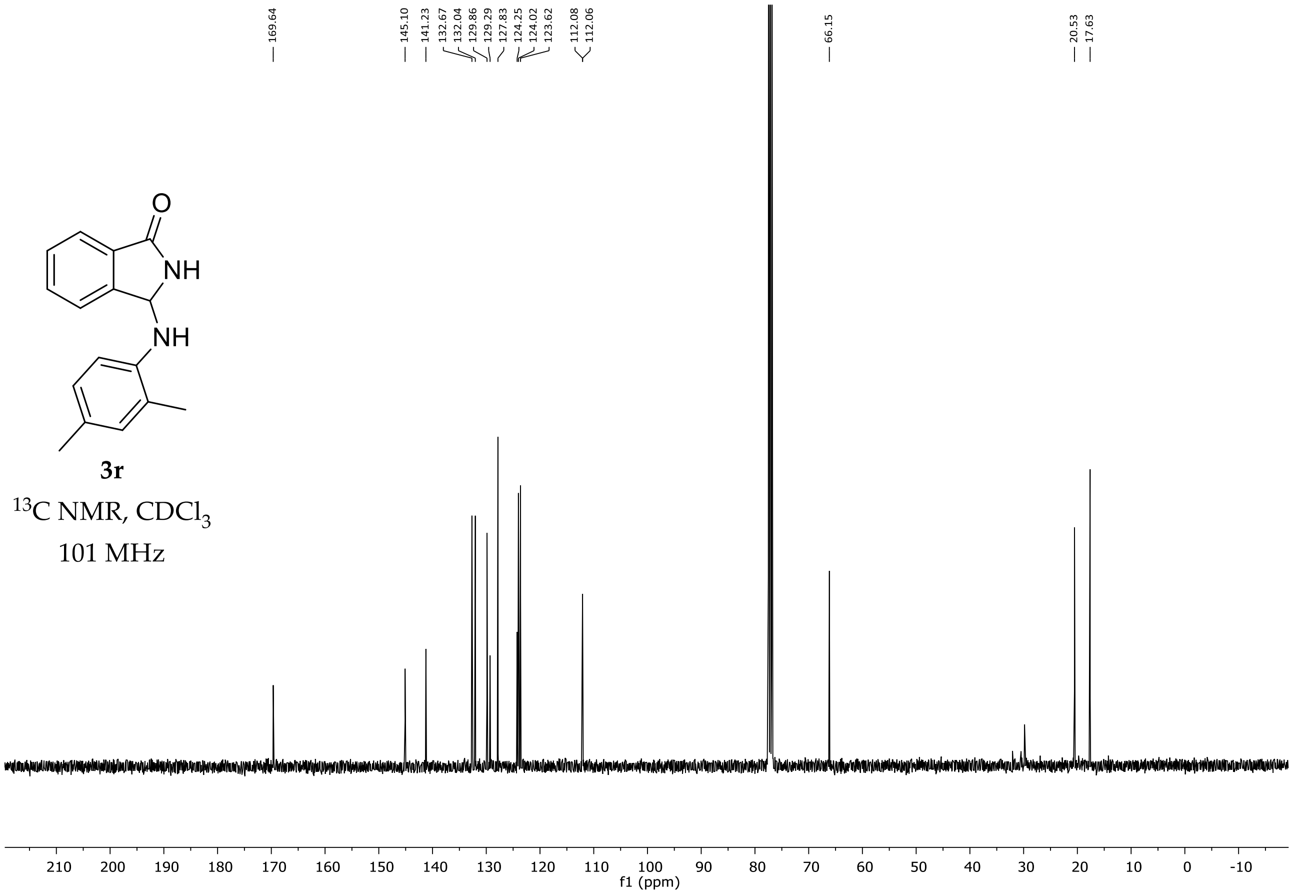

*3-((2,5-dimethoxyphenyl) amino) isoindolin-1-one (**3s**)*

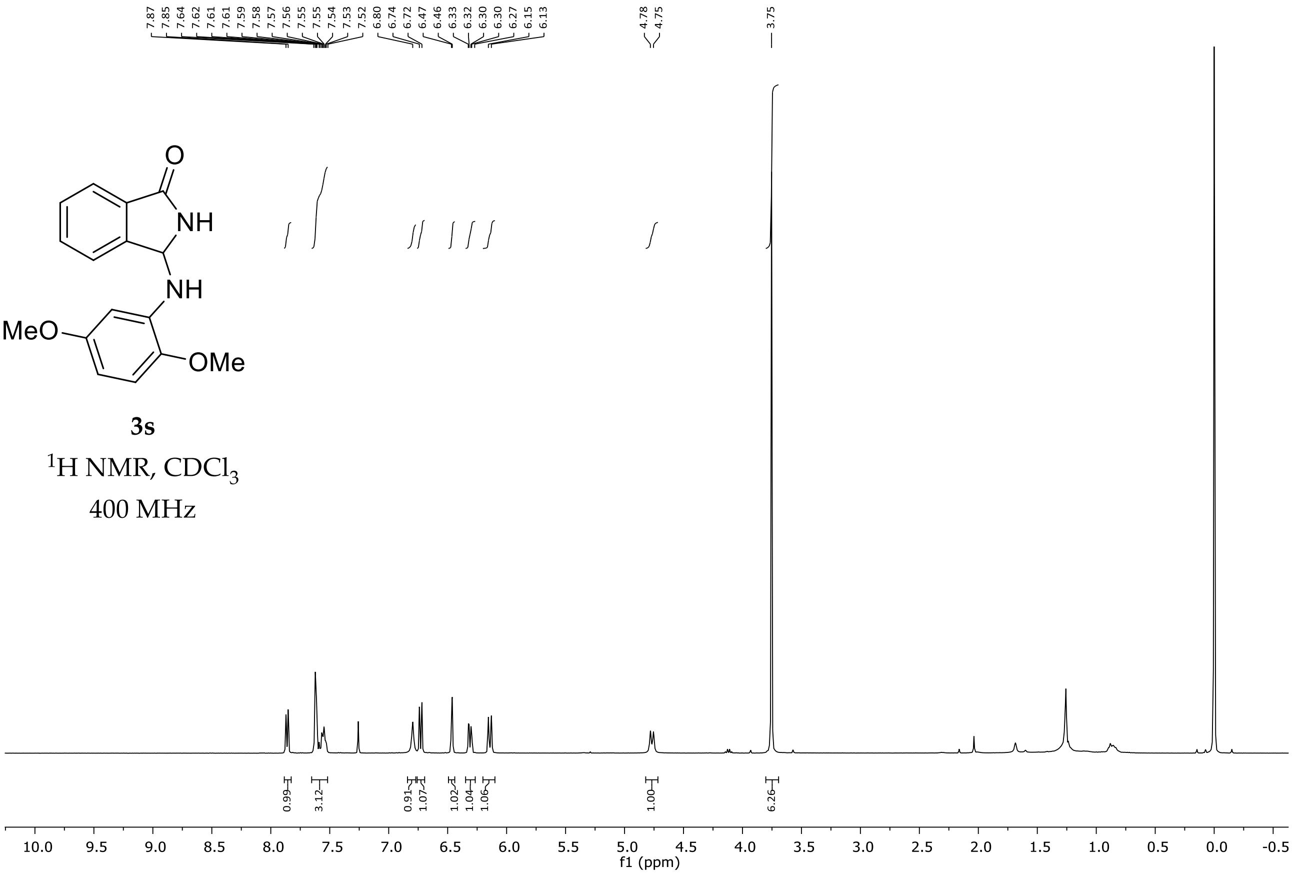

3s
^{1}H NMR, $CDCl_3$
400 MHz

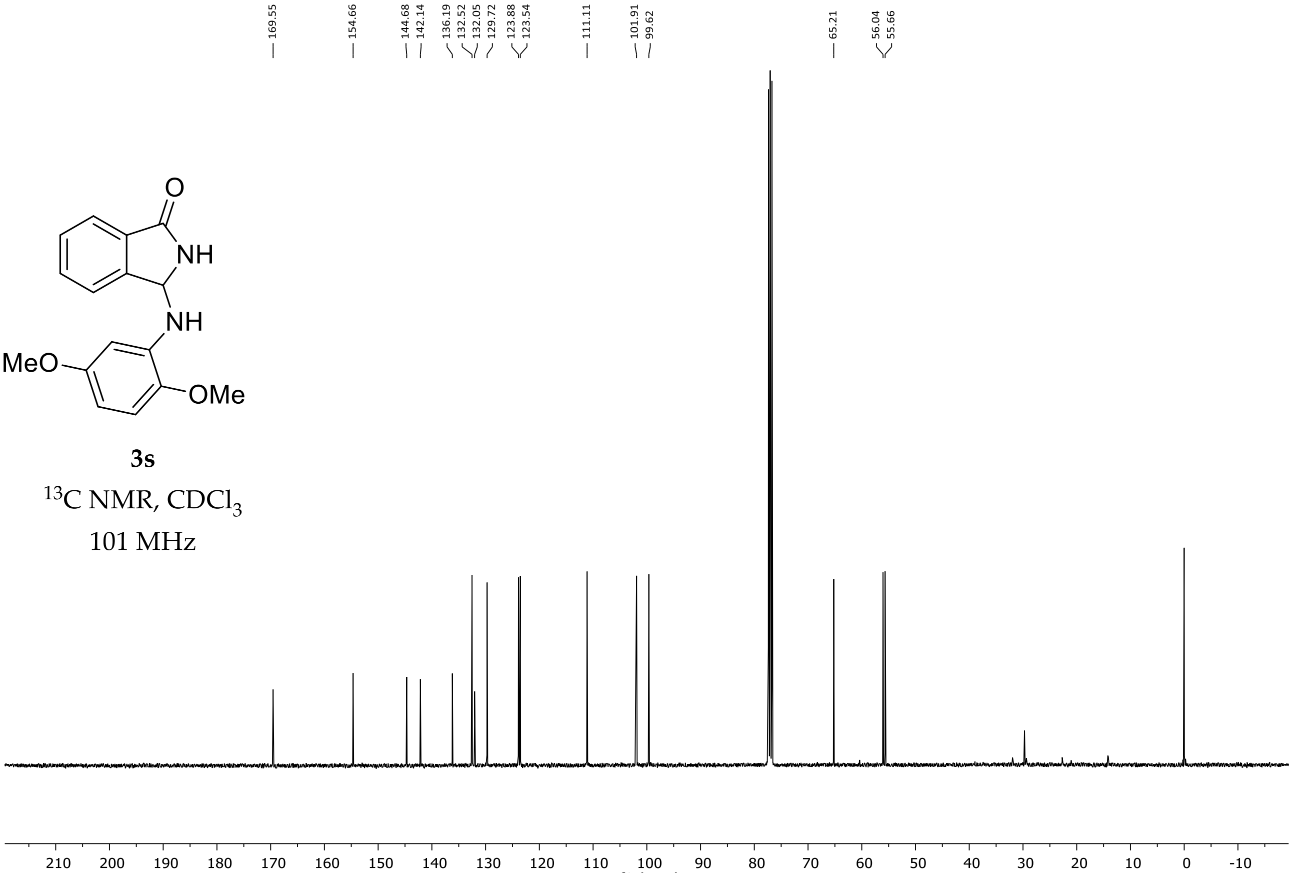

3s
^{13}C NMR, $CDCl_3$
101 MHz

*3-((2-ethynylphenyl) amino) isoindolin-1-one (**3t**)*

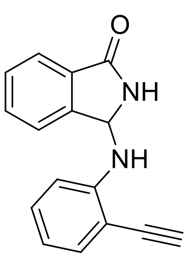

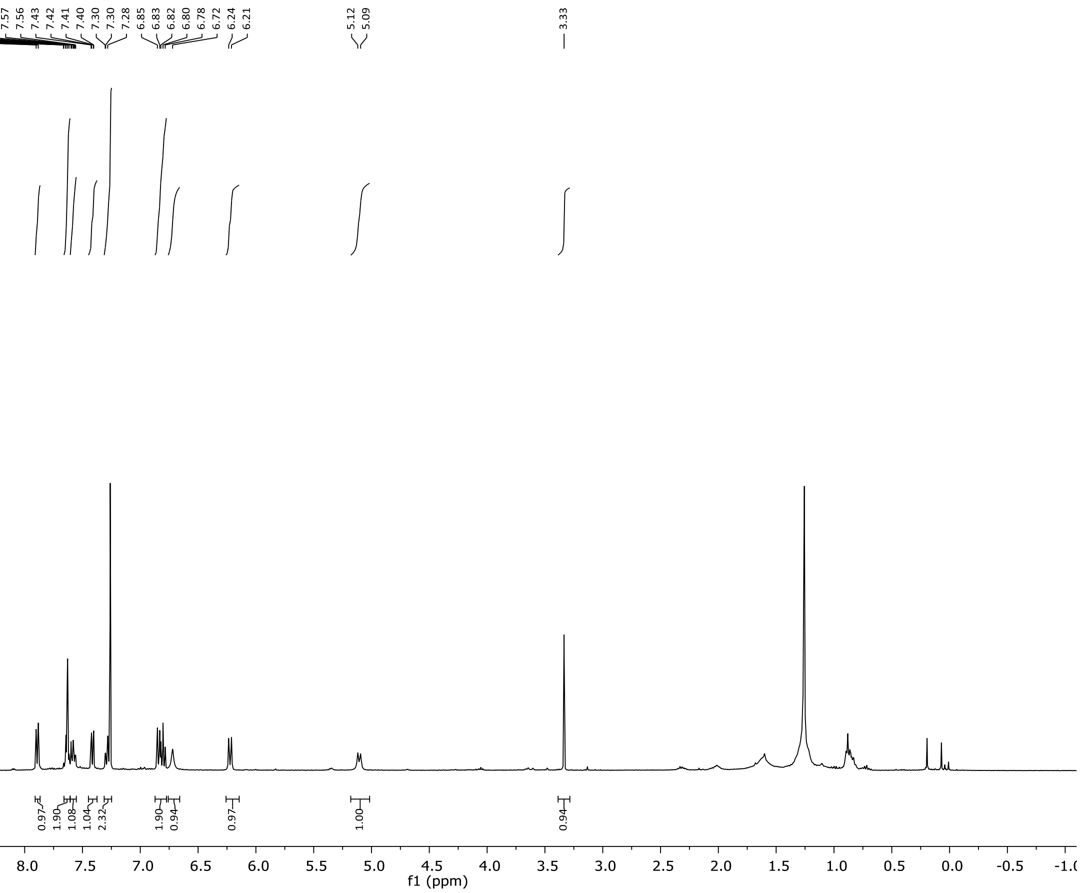

3t

^{1}H NMR, $CDCl_3$

400 MHz

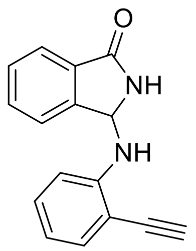

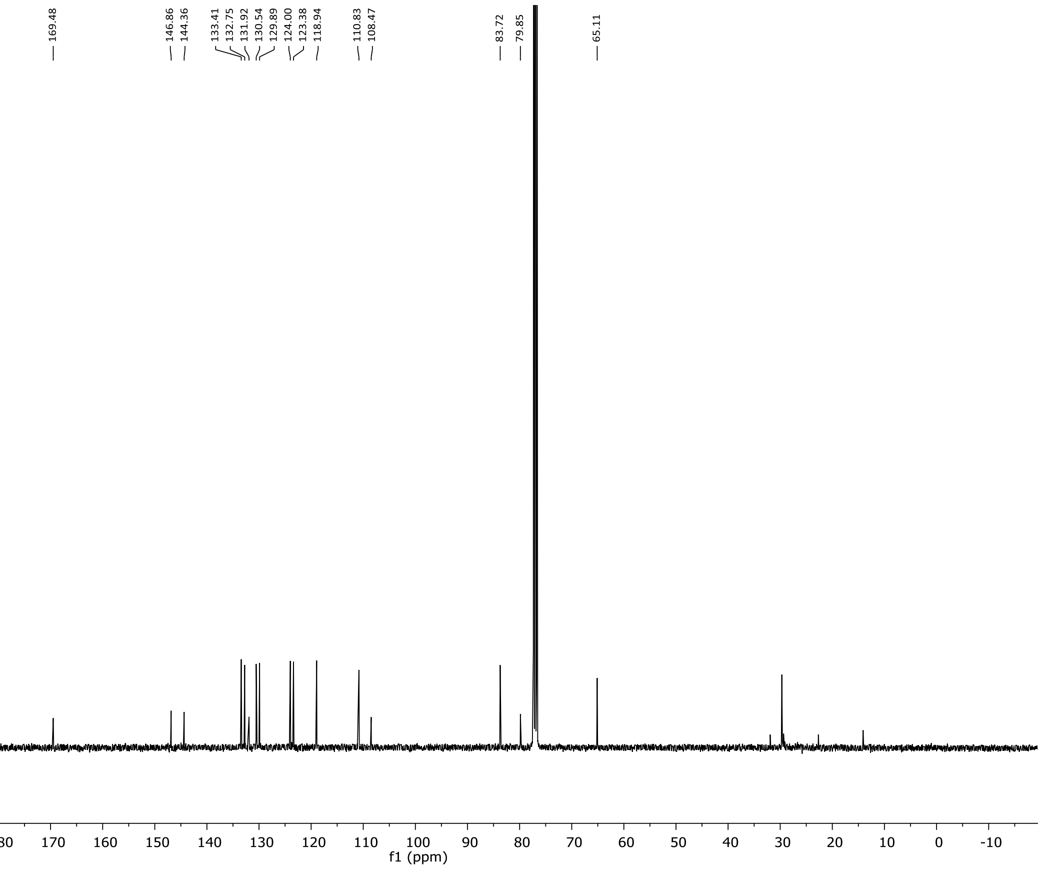

3t

^{13}C NMR, $CDCl_3$

101 MHz

*3-((2-(phenylethynyl) phenyl) amino) isoindolin-1-one (**3u**)*

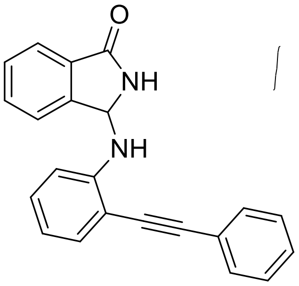

3u

^{1}H NMR, $CDCl_3$

400 MHz

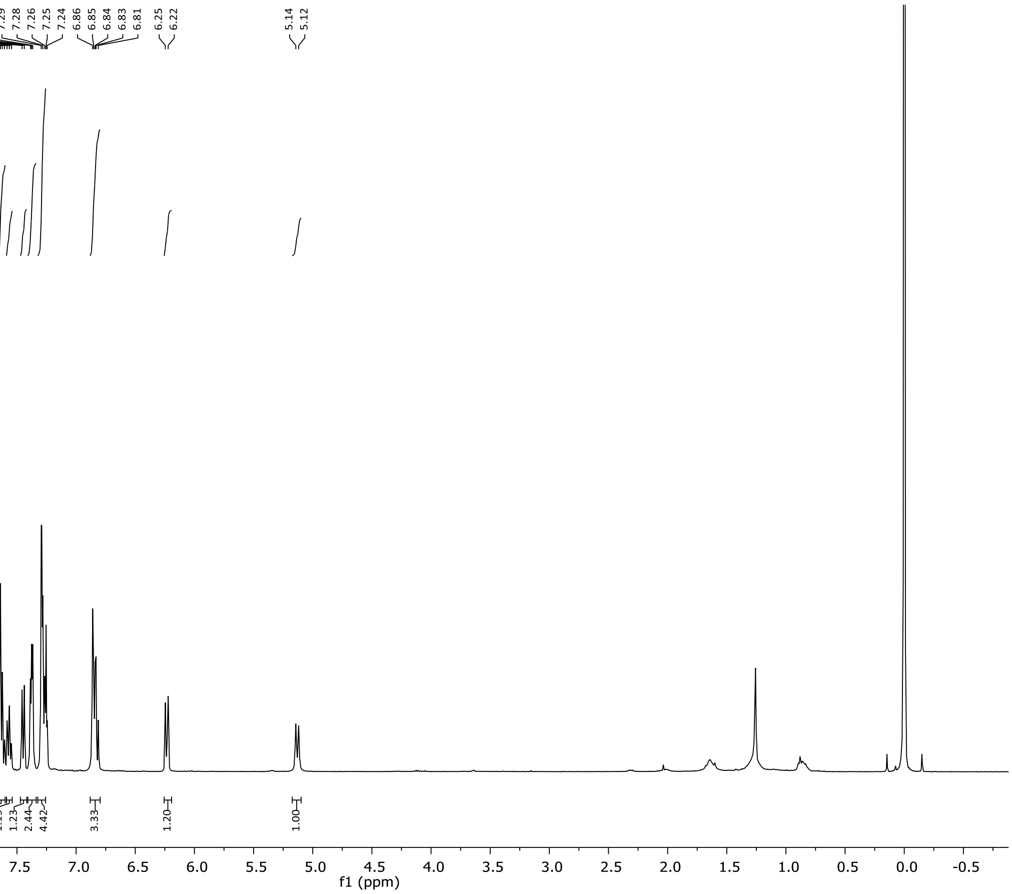

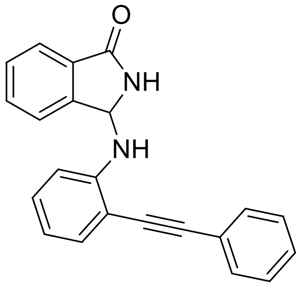

3u

^{13}C NMR, $CDCl_3$

101 MHz

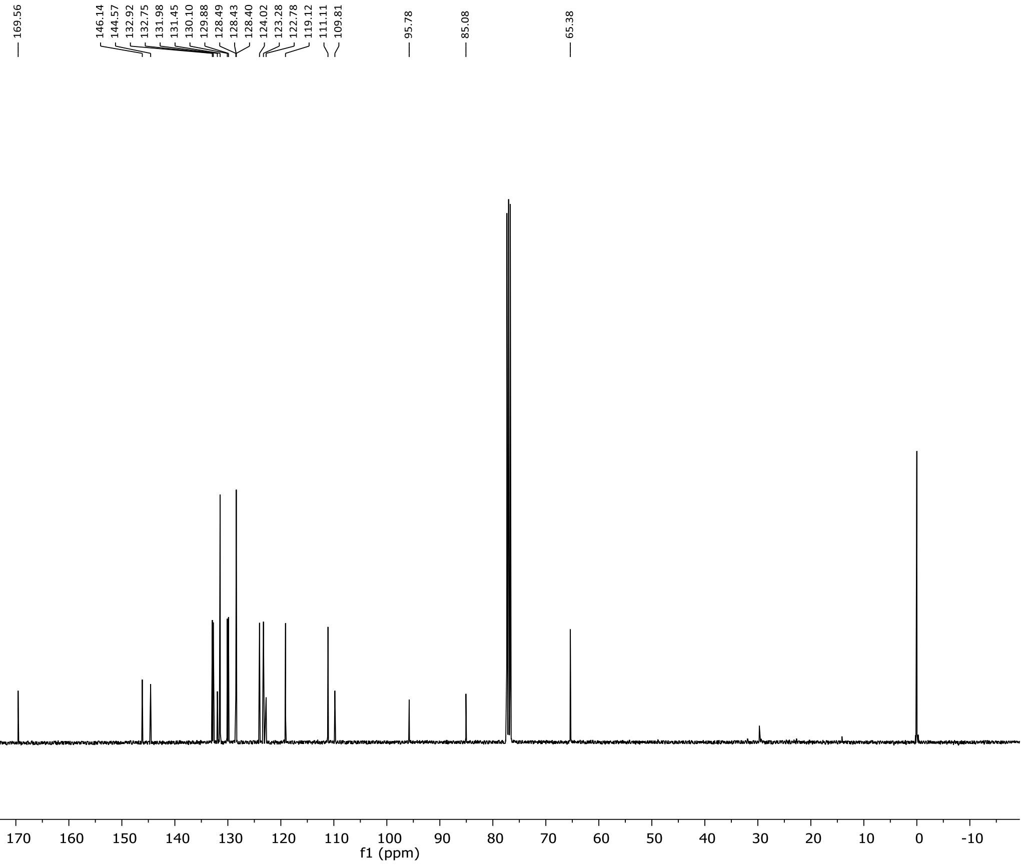

*3-((4-(phenylethynyl) phenyl) amino) isoindolin-1-one (**3v**)*

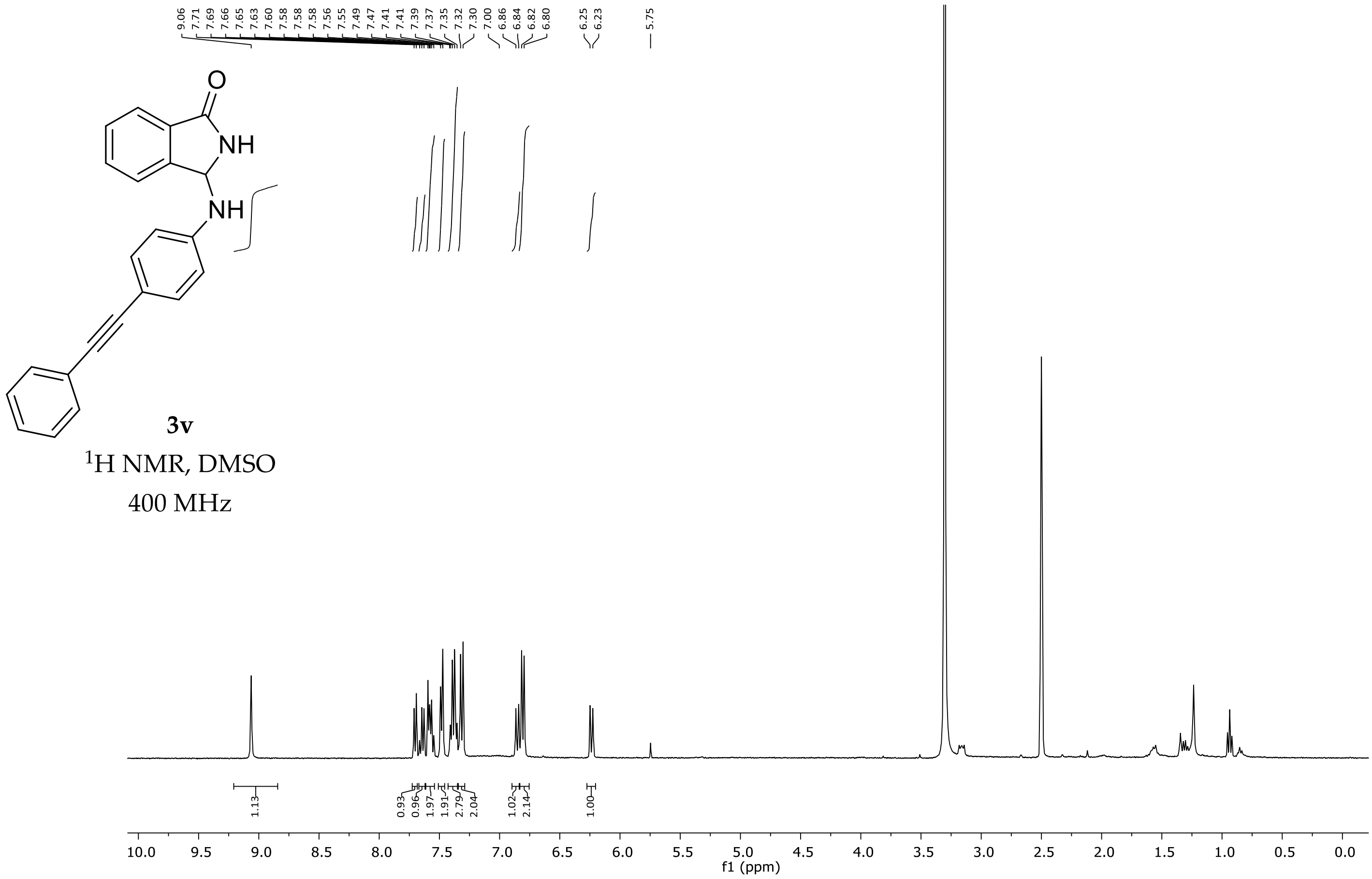

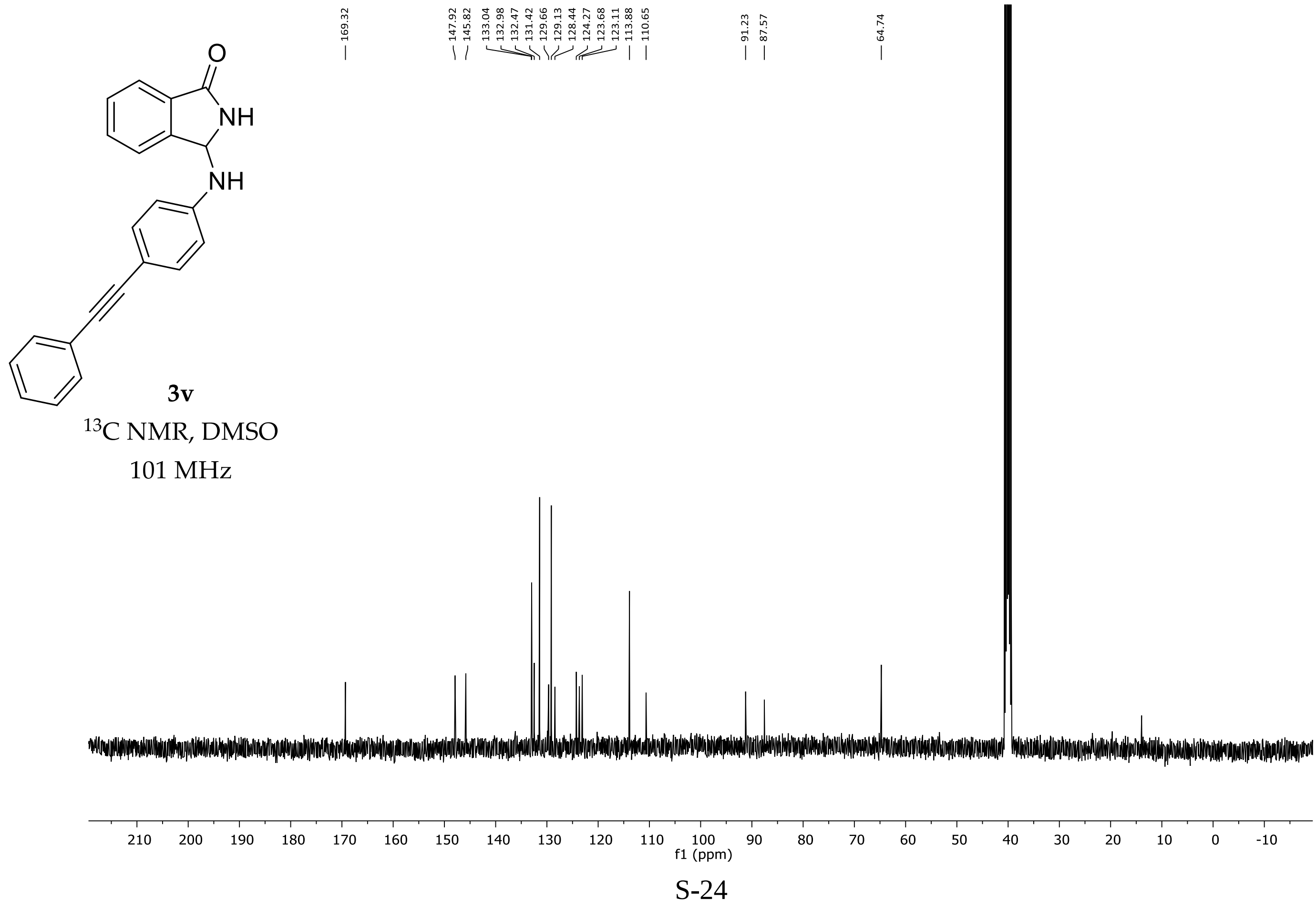

2D NMR Spectra

The complete assignment of the ^{1}H and ^{13}C NMR chemical shifts of **3a** (Figure S-2, S-3 and S-4) were based on a combination of ^{1}H and ^{13}C NMR, ^{1}H-^{1}H-COSY, NOESY, HSQC and HMBC experiments; all NMR spectra were acquired in deuterated DMSO.

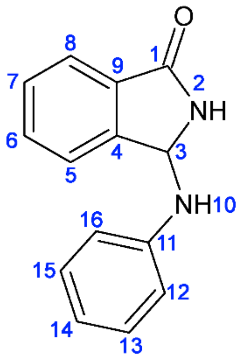

Figure S-2: Structure arbitrary numeration of compound **3a**.

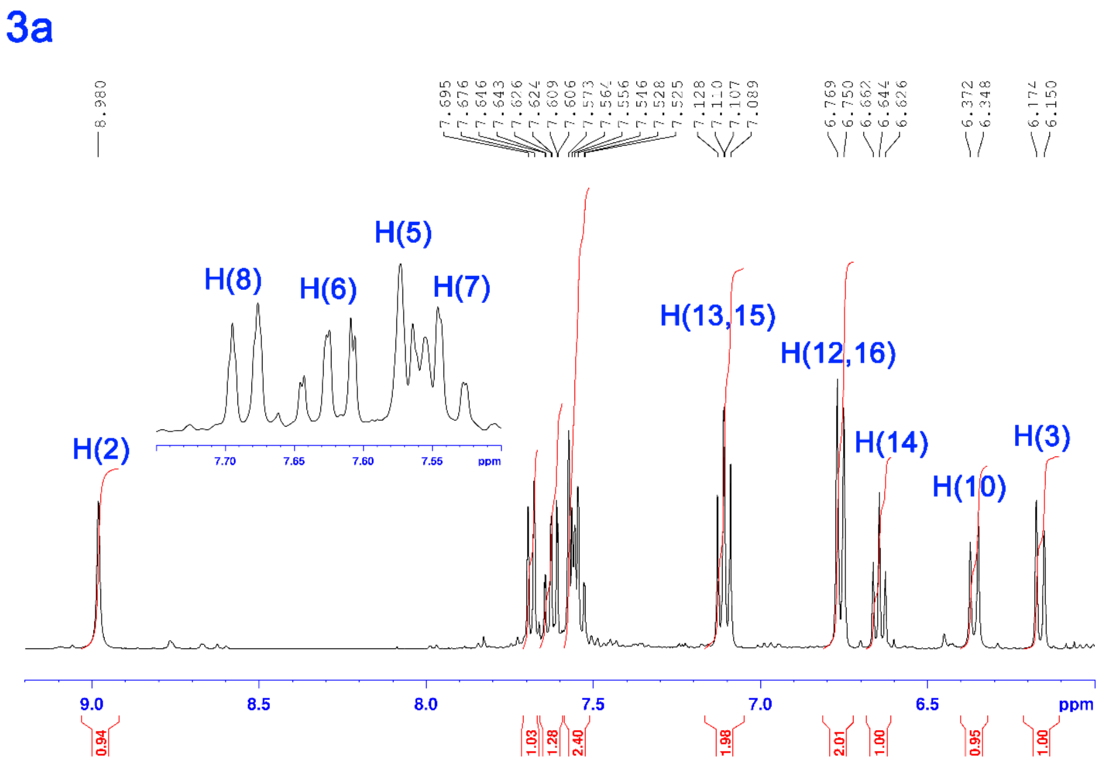

Figure S-3: ^{1}H NMR of compound **3a;** DMSO-d6.

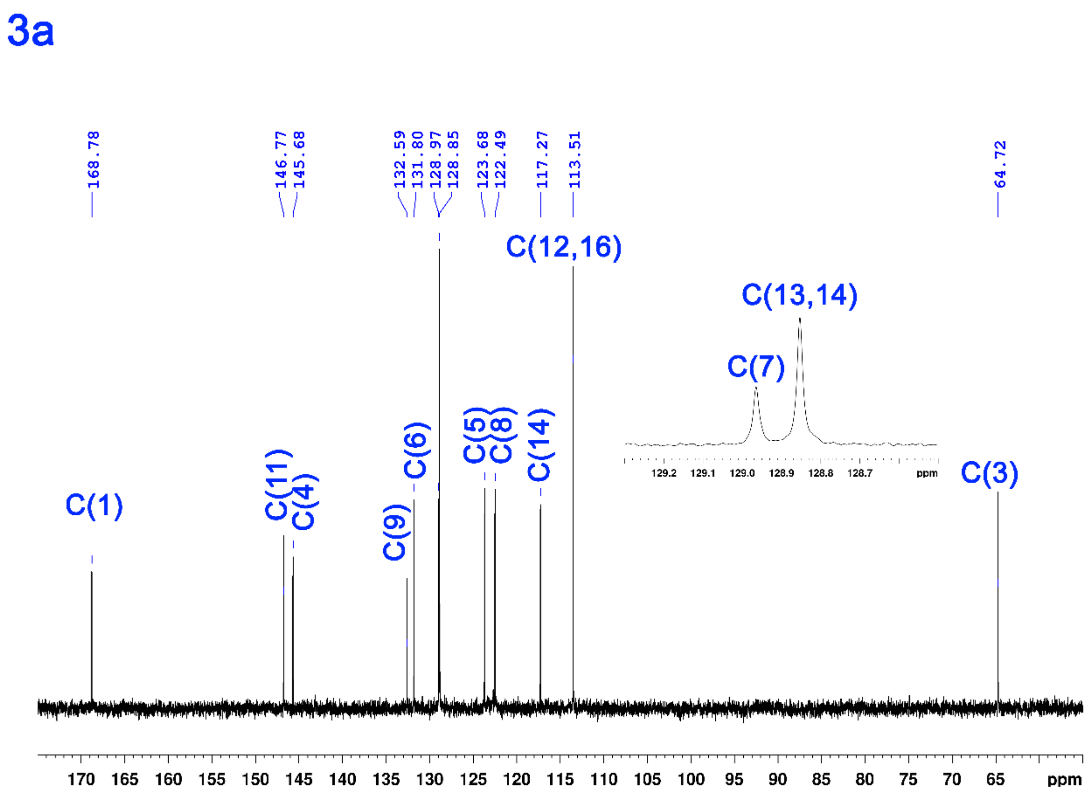

Figure S-4: ^{13}C NMR of compound **3a**; DMSO-d6.

The aromatic methine resonances appearing at 7.11, 6.76 and 6.64 ppm were attributed to aniline protons H(13,15), H(12,16) and H(14), respectively (Figure S-3), based on correlations in the HSQC spectrum (Figure S-5) with the resonances of carbons appearing at 128.9 and 113.5 ppm, attributed to *meta-* and *orto-* symmetric carbons, and on the NOESY interactions of resonance at 6.64 ppm with only the protons at 7.11 ppm (Figure S-6), showing that the proton at 6.64 ppm was the *para-* and the ones at 7.11 ppm were the *meta-* protons.

The lack of correlations in the ^{1}H-^{13}C HSQC spectrum (Figure S-5), showed that proton resonances at 8.98 and 6.36 ppm, can be attributed to endo- and exocyclic NH, whereas the two bonds scalar interaction of the proton at 8.98 ppm with the amide carbonyl and the three bonds scalar interaction of the proton at 6.36, as seen in the HMBC spectrum (Figure S-8), allowed to assign the proton resonances of endocyclic and exocyclic nitrogen at 8.98 and 6.36 ppm, respectively, assignation further corroborated by the NOESY interaction of exocyclic NH with H(5) (Figure S-6).

The benzyl proton at 6.16 ppm, can be easily identified due to the presence of the only characteristic high-field resonance on ^{13}C at 64.7 ppm according to correlations in the HSQC spectrum (Figure S-5).

The skeleton bond connections, as shown in the **3a** compound (Figure S-2), was further confirmed by scalar coupling interactions between benzyl proton at 6.16 ppm and endocyclic and exocyclic nitrogen at 8.98 and 6.36 ppm, respectively, as seen in the COSY spectrum (Figure S-7).

The remaining methine resonances of the isoindolinone, appearing between 7.70 and 7.50 ppm, were assigned identifying the H(8) by the three bonds scalar interaction with amide carbonyl, as seen in the HMBC spectrum (Figure S-8), at 7.69 ppm, followed by the dipolar coupling (Figure S-9), showed between protons at 7.69 and 7.55 ppm and the proton at 7.57 ppm with *orto*- aniline, exocyclic NH and benzyl proton at 6.76, 6.36 and 6.16 ppm, respectively. The assignation was further confirmed by scalar coupling between the proton at 7.69 ppm, H(8), and proton at 7.55 ppm, H(7), and between the proton at 7.57 ppm, H(5), and benzyl proton at 6.16 ppm, as seen in COSY spectrum (Figure S-10A and S10B, respectively).

The remaining three quaternary carbons of the isoindolinone and aniline were assigned as follows: carbon at 146.8 ppm interacted through four bonds with the *para*- proton at 6.64 ppm, the carbon at 145.7 ppm interacted through three bonds with the exocyclic NH whereas the carbon at 132.6 ppm lacked the four bonds interaction with the same exocyclic NH and showed a three bonds interaction with the benzyl proton at 6.16 ppm (Figure S-8).

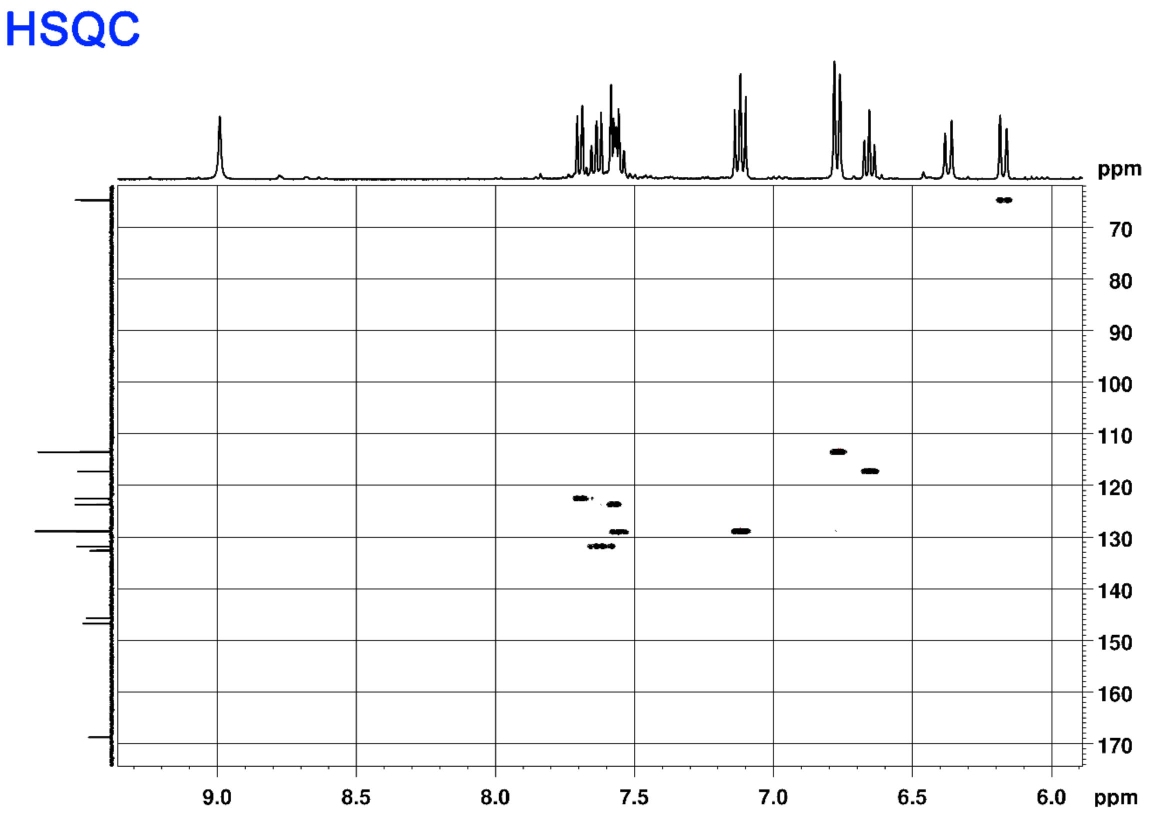

Figure S-5: HSQC spectrum of **3a**; DMSO-d6.

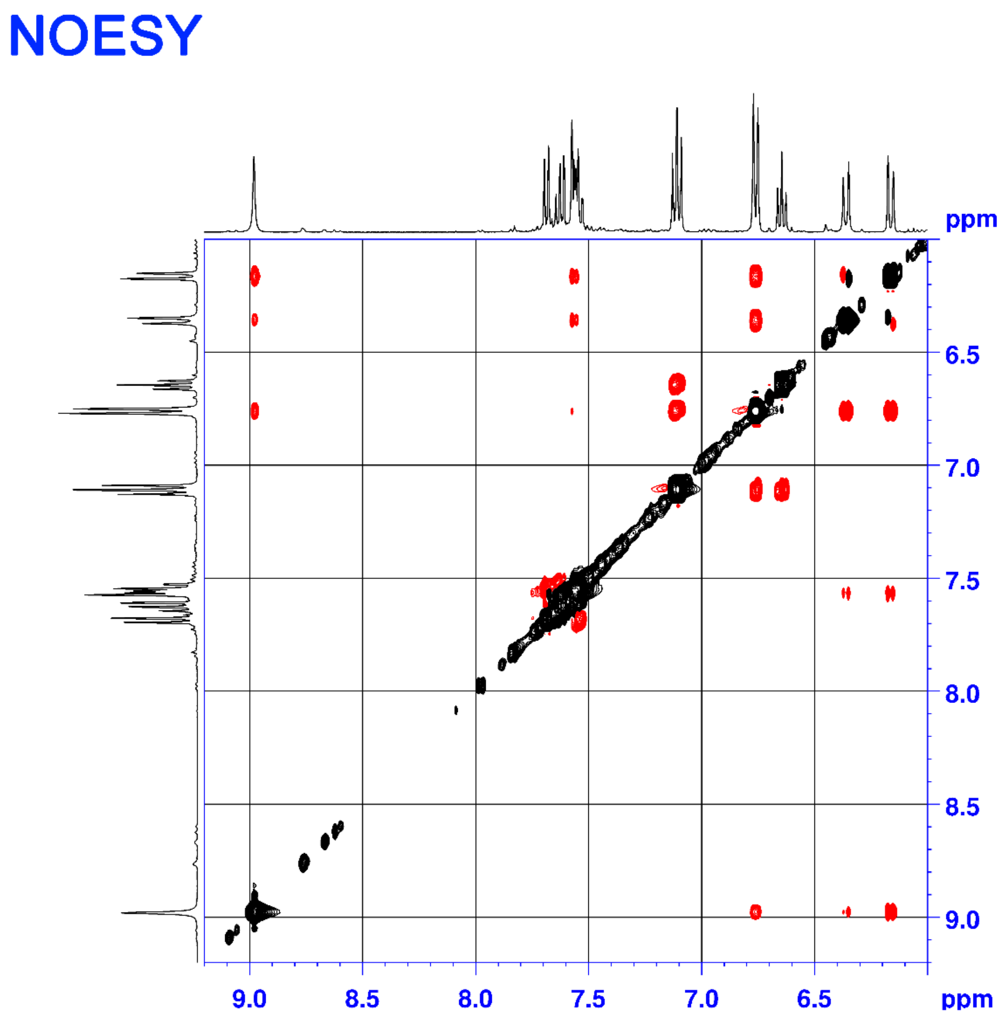

Figure S-6: NOESY spectrum of **3a**; DMSO-d6.

COSY

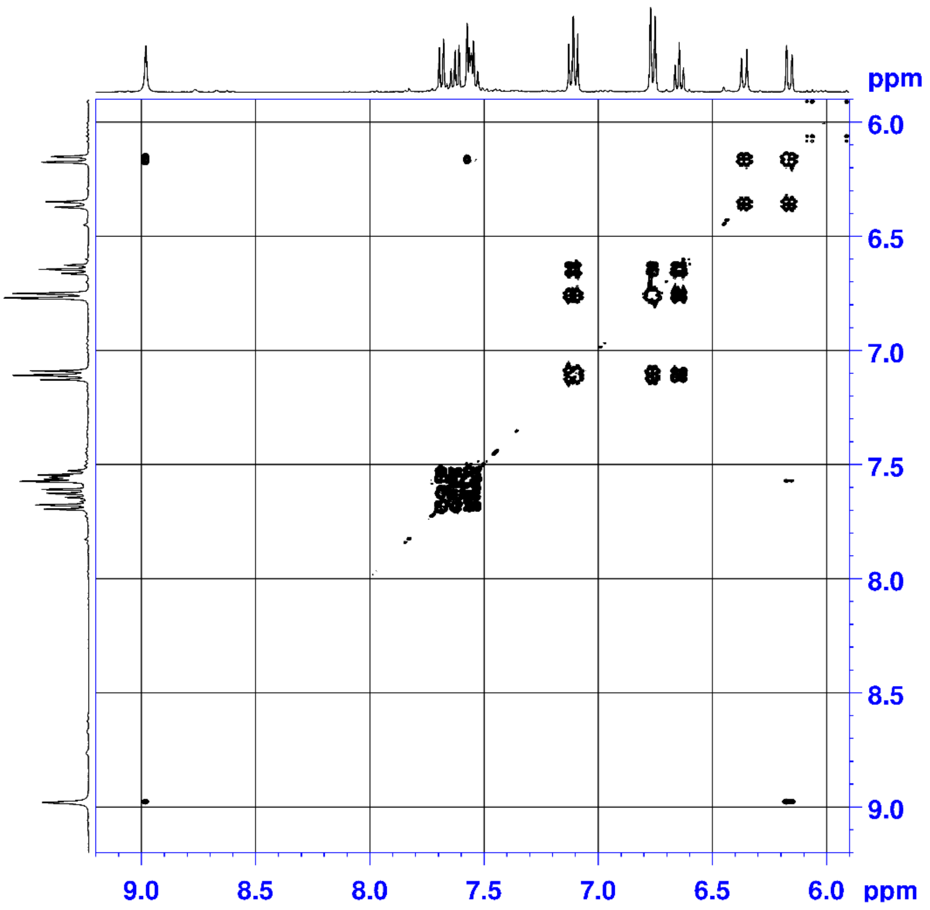

Figure S-7: COSY spectrum of **3a**; DMSO-d6.

HMBC

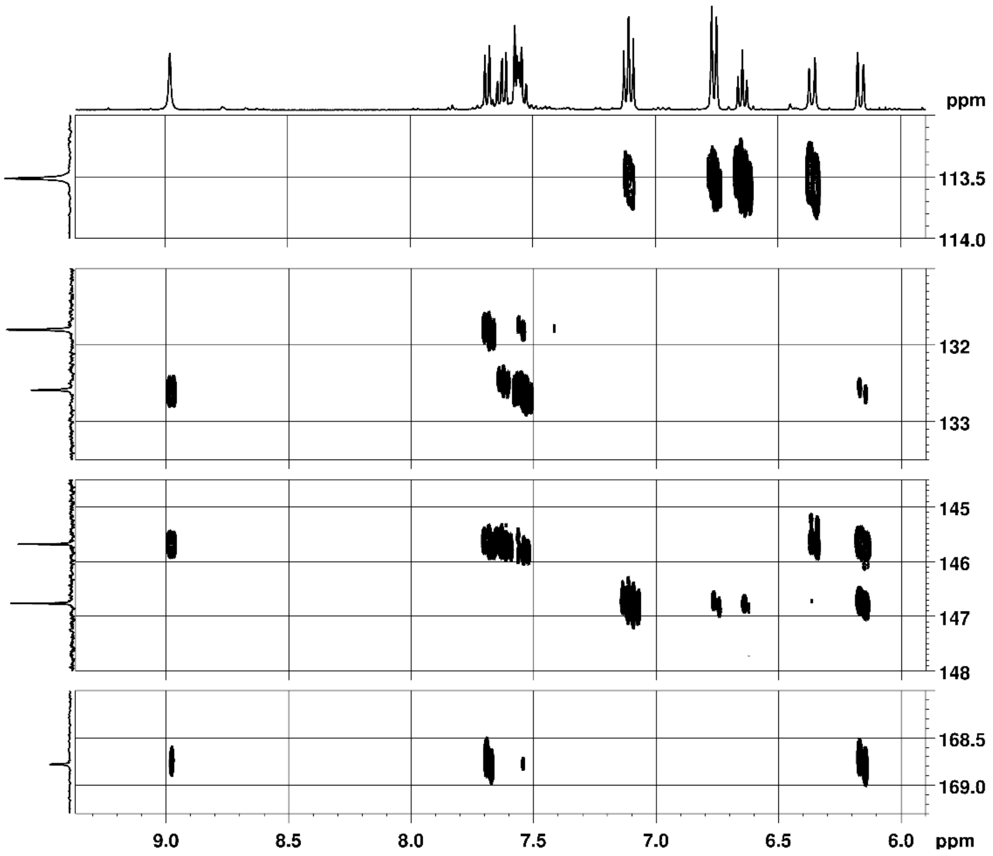

Figure S-8: HMBC spectrum of **3a**; DMSO-d6.

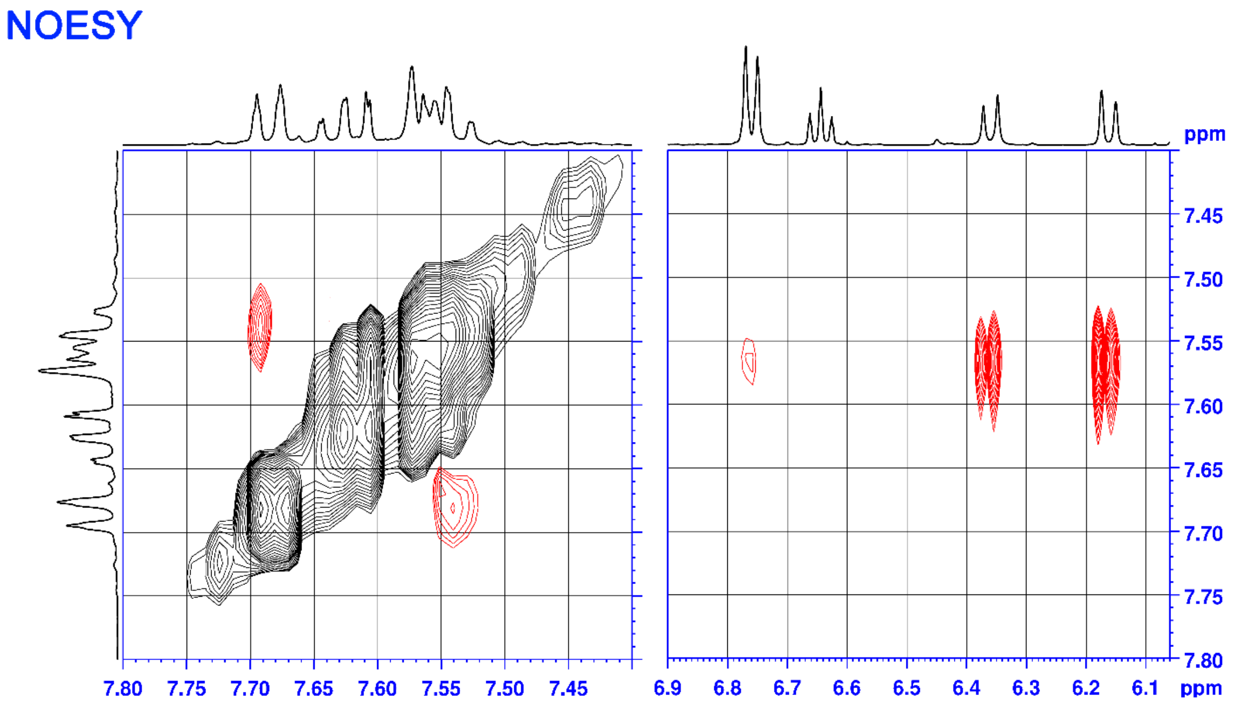

Figure S-9: expansion of NOESY spectrum of **3a**; DMSO-d6.

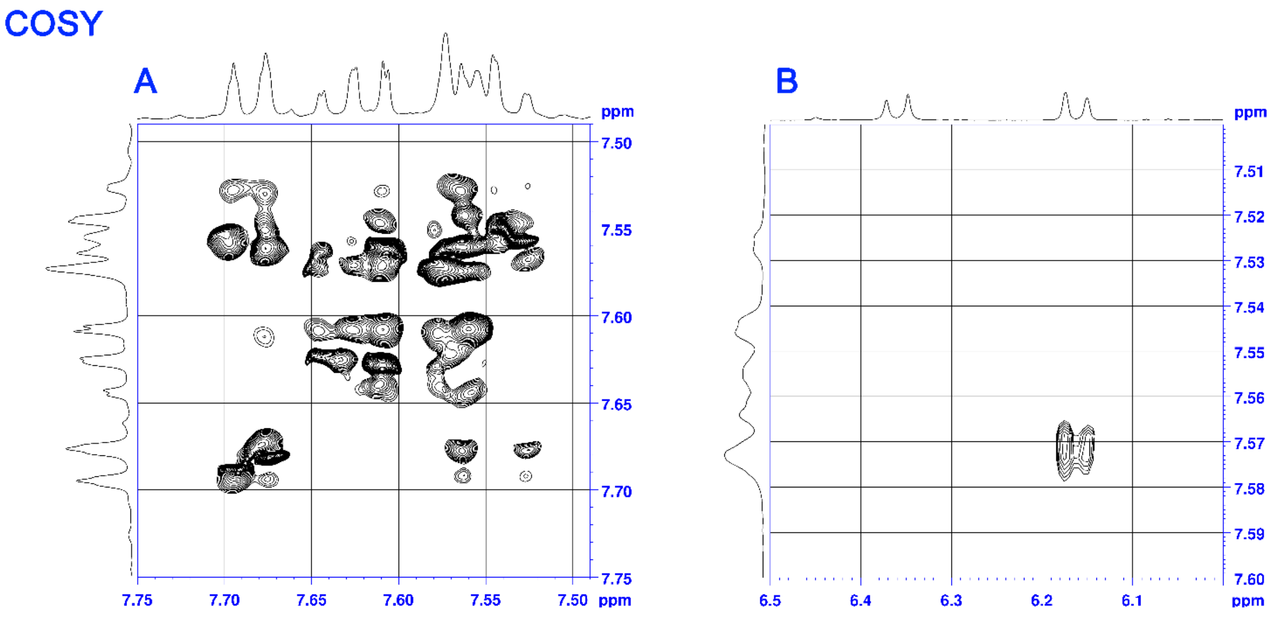

Figure S-10: expansions of COSY spectrum of **3a**; DMSO-d6.

DFT Data

A) Cartesian Coordinates corresponding to the optimized critical points

I) Species CH_3CN

C	0.011069	0.023267	0.010005
H	0.021666	0.034845	1.102963
H	1.041865	0.023777	-0.353566
C	-0.689255	-1.165478	-0.472313
H	-0.491317	0.925298	-0.348650
N	-1.244273	-2.107864	-0.848783

II) Species CH_3CN^- reduced

C	0.003565	0.010468	-0.045623
H	-0.061502	-0.169545	1.047071
H	1.069849	0.106790	-0.309591
C	-0.682607	-1.110337	-0.917280
H	-0.449988	0.994317	-0.255331
N	-1.229561	-2.097849	-0.429590

III) Species CH_3CN^- deprotonated

C	0.098296	0.000002	0.016508
C	0.059668	0.000001	1.406197
N	-0.028916	-0.000001	2.588081
H	0.467012	-0.915704	-0.449595
H	0.466972	0.915725	-0.449595

IV) Species 1

C	0.073805	0.000000	-0.018299
O	0.235570	0.000000	1.177408
H	0.940572	0.000000	-0.713243
C	-1.245893	0.000000	-0.701429
C	-2.465995	-0.000000	-0.000618
C	-3.670151	-0.000000	-0.709094
C	-3.666600	-0.000000	-2.100482

C	-2.462804	-0.000000	-2.797635
C	-1.263128	0.000000	-2.094963
C	-2.547016	-0.000000	1.436183
H	-4.605912	-0.000000	-0.160605
H	-4.609497	-0.000000	-2.637614
H	-2.458092	-0.000000	-3.882658
H	-0.318109	0.000000	-2.632818
N	-2.746310	-0.000000	2.577685

V) Species 1^{-} reduced

C	0.077581	0.000000	-0.040831
O	0.311315	0.000000	1.180720
H	0.933410	0.000000	-0.762869
C	-1.195011	0.000000	-0.678410
C	-2.461765	-0.000000	0.027481
C	-3.664651	-0.000000	-0.710046
C	-3.684533	-0.000000	-2.089392
C	-2.445451	-0.000000	-2.794273
C	-1.263709	0.000000	-2.103106
C	-2.586882	-0.000000	1.443231
H	-4.600773	-0.000000	-0.154915
H	-4.628696	-0.000000	-2.627440
H	-2.440441	-0.000000	-3.882746
H	-0.319644	0.000000	-2.648974
N	-2.840310	-0.000000	2.583386

VI) Specie 1^{-} deprotonated

C	0.012255	0.101292	0.064601
C	0.000234	0.066721	1.543279
C	1.344489	0.027952	2.192782
C	2.488468	-0.066931	1.381642
C	2.454258	-0.088699	0.005186
C	1.171243	0.019805	-0.630827

C	-1.152689	0.017288	2.190688
O	-2.208103	0.085549	2.744407
C	1.470735	0.087255	3.589512
N	1.567137	0.131122	4.757466
H	3.447334	-0.112052	1.897196
H	3.363857	-0.155189	-0.582767
H	1.120637	0.052326	-1.719466
H	-0.939369	0.194923	-0.452563

VII) Species 2a

C	0.024155	0.035679	-0.044981
C	0.013036	-0.010771	1.355048
C	1.205333	-0.036293	2.068108
C	2.431162	-0.015765	1.407844
C	2.445225	0.029235	0.016262
C	1.258674	0.054895	-0.706522
H	-0.937842	-0.032703	1.882326
H	1.172960	-0.071221	3.153527
H	3.360095	-0.034690	1.968568
H	3.391290	0.045927	-0.517673
H	1.283163	0.084038	-1.793342
N	-1.170119	0.008449	-0.767992
H	-1.975339	0.355968	-0.266133
H	-1.112562	0.410279	-1.693319

VIII) Species $2a^{-}$ reduced

C	0.029306	0.004805	-0.045087
C	-0.026800	-0.026789	1.342711
C	1.215754	-0.033604	2.101497
C	2.425435	0.000241	1.402681
C	2.479754	0.043914	0.014846
C	1.240137	0.068190	-0.747361
H	-0.993175	-0.026828	1.849633

H	1.196848	-0.086670	3.188837
H	3.362402	-0.009337	1.967809
H	3.438650	0.078459	-0.501602
H	1.243907	-0.030406	-1.833247
N	-1.194242	-0.049540	-0.799948
H	-1.942512	0.362749	-0.248982
H	-1.086237	0.527844	-1.630067

IX) Species 2a$^-$ deprotonated

C	0.022119	0.075987	-0.045320
C	-0.055681	0.033309	1.399374
C	1.241116	-0.018075	2.038768
C	2.424910	-0.024449	1.330612
C	2.456659	0.018497	-0.073737
C	1.223211	0.068405	-0.733298
N	-1.161120	0.031921	2.130329
H	1.246674	-0.052489	3.127306
H	3.366012	-0.064250	1.883958
H	3.395598	0.012826	-0.622505
H	1.201344	0.102934	-1.825208
H	-0.915915	0.115534	-0.603762
H	-1.955290	0.072159	1.480360

X) Species B

C	0.461428	0.645176	-0.114183
C	0.429642	0.902606	1.259976
C	1.057841	0.029775	2.139879
C	1.722089	-1.094664	1.653984
C	1.753378	-1.348830	0.288316
C	1.123800	-0.489210	-0.608958
H	-0.083509	1.785071	1.627191
H	1.029203	0.229642	3.206193
H	2.213601	-1.774182	2.343182

H	2.262051	-2.218787	-0.114218
C	1.157700	-0.817124	-2.067923
C	-0.168956	1.586683	-0.999893
N	-0.672606	2.381798	-1.676793
H	0.507485	-0.212853	-2.722691
N	2.488771	1.594542	-2.787485
O	1.830287	-1.724105	-2.505248
C	2.133017	1.433984	-4.137740
H	2.245754	2.500813	-2.408497
H	3.454348	1.366844	-2.589116
C	1.073511	2.168156	-4.682215
C	0.681754	1.953901	-5.998760
C	1.330727	1.010111	-6.790339
C	2.375557	0.270836	-6.242520
C	2.774964	0.474462	-4.927073
H	0.547313	2.890579	-4.064167
H	-0.142047	2.532800	-6.406345
H	1.021540	0.847344	-7.817927
H	2.885266	-0.478503	-6.841232
H	3.573435	-0.125915	-4.499273

XI) Species AH

C	0.154647	-0.089373	-0.228586
C	-0.074080	0.055439	1.143179
C	1.006462	0.362719	1.980268
C	2.280556	0.527237	1.456514
C	2.504863	0.401986	0.087096
C	1.434002	0.095100	-0.744546
N	-1.348166	-0.052956	1.716229
C	-2.498675	-0.416942	0.964500
O	-2.410301	-1.772437	0.546215
C	-3.732616	-0.177487	1.825497

C	-4.273582	1.110652	1.966382
C	-5.379280	1.332819	2.791816
C	-5.947317	0.272400	3.487696
C	-5.414326	-1.005744	3.354970
C	-4.315888	-1.226678	2.527268
C	-3.703955	2.219025	1.248615
N	-3.266498	3.114723	0.657135
H	-5.784918	2.335112	2.880519
H	-6.805205	0.445753	4.129142
H	-5.855099	-1.837004	3.896711
H	-3.898939	-2.221559	2.410555
H	-2.542636	0.239003	0.083121
H	-1.356556	-0.378121	2.672968
H	0.834829	0.487132	3.046789
H	3.103268	0.765118	2.124707
H	3.500574	0.537037	-0.322949
H	1.591671	-0.019351	-1.813222
H	-0.652066	-0.378237	-0.892255
H	-3.111445	-1.934195	-0.098838

XII) Species A-H reduced

C	0.222100	-0.352530	-0.182271
C	-0.063247	-0.083923	1.168019
C	0.960973	0.450148	1.970038
C	2.216551	0.709898	1.440130
C	2.492424	0.453435	0.098128
C	1.481570	-0.077425	-0.699856
N	-1.299286	-0.363814	1.712062
C	-2.505671	-0.435695	0.911479
O	-2.525340	-1.720009	0.282170
C	-3.719892	-0.195109	1.767352
C	-4.200179	1.164822	1.971700

C	-5.328846	1.329423	2.853609
C	-5.910961	0.261845	3.480809
C	-5.420199	-1.063532	3.293300
C	-4.321577	-1.243751	2.439674
C	-3.622525	2.270338	1.340376
N	-3.138291	3.209047	0.817837
H	-5.714629	2.334281	3.011442
H	-6.762902	0.432174	4.138186
H	-5.880445	-1.909124	3.795725
H	-3.914062	-2.242587	2.288679
H	-2.446041	0.336256	0.124781
H	-1.465046	-0.000458	2.638523
H	0.754322	0.670376	3.015038
H	2.986772	1.128327	2.084120
H	3.474616	0.663091	-0.315870
H	1.676098	-0.290983	-1.748496
H	-0.544472	-0.806127	-0.800971
H	-3.442493	-1.837219	0.001781

XIII) Path for the formation of B (Ts-1)

Point 1

C	0.000000	0.000000	0.000000
C	0.000000	0.000000	1.399018
C	1.221614	0.000000	2.081377
C	2.416545	-0.008147	1.370671
C	2.417424	-0.022740	-0.021456
C	1.201014	-0.014942	-0.698921
N	-1.211701	0.078831	2.104340
C	-1.505131	2.917065	1.355457
O	-1.798781	2.957217	0.182239
C	-2.508526	3.174701	2.433568
C	-2.212491	2.982801	3.792340

C	-3.174337	3.255801	4.770062
C	-4.429246	3.718586	4.393760
C	-4.730715	3.904565	3.046103
C	-3.774614	3.632618	2.075504
C	-0.934900	2.476852	4.217398
N	0.076070	2.066877	4.609737
H	-2.932289	3.099779	5.816017
H	-5.172637	3.932648	5.154917
H	-5.712263	4.264675	2.754198
H	-3.984268	3.770791	1.019702
H	-0.472075	2.711750	1.685528
H	-1.147506	-0.227546	3.066013
H	-1.996037	-0.350742	1.632433
H	1.230422	0.031405	3.167393
H	3.356121	-0.003997	1.915760
H	3.353228	-0.032449	-0.571193
H	1.182301	-0.013606	-1.784970
H	-0.944385	0.036035	-0.536512

Point 2

C	0.000000	0.000000	0.000000
C	0.000000	0.000000	1.399091
C	1.224547	0.000000	2.078181
C	2.417898	-0.009987	1.364829
C	2.417021	-0.018667	-0.027419
C	1.199258	-0.011047	-0.701838
N	-1.206508	0.092883	2.110113
C	-0.759365	3.243533	1.449831
O	-0.751938	3.558937	0.281902
C	-1.898059	3.577645	2.356757
C	-1.920448	3.181546	3.703640
C	-2.999654	3.527175	4.522920

C	-4.056831	4.261256	3.999347
C	-4.041984	4.653279	2.662210
C	-2.967696	4.312830	1.850060
C	-0.851600	2.410363	4.279880
N	-0.022279	1.775606	4.783134
H	-3.002907	3.216263	5.562225
H	-4.893035	4.527656	4.637834
H	-4.868892	5.226887	2.255472
H	-2.930872	4.608713	0.806621
H	0.084063	2.692662	1.901989
H	-1.155096	-0.277198	3.050279
H	-2.003871	-0.285107	1.615448
H	1.234644	0.026336	3.164436
H	3.358400	-0.009619	1.908480
H	3.351658	-0.025327	-0.579008
H	1.178118	-0.008585	-1.787792
H	-0.945154	0.024071	-0.536977

Point 3

C	0.000000	0.000000	0.000000
C	0.000000	0.000000	1.399459
C	1.224948	0.000000	2.078257
C	2.418247	0.004097	1.364708
C	2.417314	0.001887	-0.027557
C	1.199291	0.000109	-0.701747
N	-1.206917	0.083903	2.107226
C	-0.382317	3.299827	1.373833
O	-0.219705	3.657339	0.229683
C	-1.624673	3.621955	2.138001
C	-1.810583	3.216558	3.469250
C	-2.983876	3.551173	4.151903
C	-3.973017	4.283477	3.506853

C	-3.795499	4.685998	2.184666
C	-2.627254	4.357046	1.508977
C	-0.813309	2.449580	4.166361
N	-0.036701	1.811776	4.744468
H	-3.112642	3.233902	5.181268
H	-4.883202	4.540778	4.039072
H	-4.569244	5.258515	1.682880
H	-2.463318	4.661105	0.480221
H	0.388777	2.718718	1.910077
H	-1.144346	-0.237057	3.064359
H	-1.996037	-0.333713	1.632320
H	1.235456	0.018019	3.164724
H	3.358818	0.007988	1.908256
H	3.351808	0.006073	-0.579276
H	1.178289	0.005534	-1.787673
H	-0.945677	0.018455	-0.536417

XIV) Species Ts-2

C	0.335750	0.417601	0.124757
C	0.177885	0.112066	1.479688
C	1.083715	-0.729699	2.114433
C	2.154601	-1.255244	1.399527
C	2.313969	-0.941573	0.052260
C	1.414849	-0.108883	-0.605479
H	-0.657860	0.537008	2.025799
H	0.951362	-0.971891	3.163877
H	2.865348	-1.914235	1.888631
H	3.145844	-1.362307	-0.506680
C	1.570363	0.147961	-2.095217
C	-0.593190	1.343543	-0.469256
N	-1.320190	2.158067	-0.859546
O	0.471425	0.037224	-2.846487

H	2.486780	-0.363886	-2.449593
N	1.825130	1.691701	-2.390389
H	0.807113	1.379643	-2.948267
H	1.711882	2.306870	-1.584547
C	2.960211	2.039698	-3.200707
C	3.921855	2.933428	-2.745004
C	5.008573	3.242863	-3.559395
C	5.132511	2.654568	-4.813673
C	4.163580	1.756499	-5.259091
C	3.071595	1.447991	-4.458383
H	3.825861	3.386510	-1.761658
H	5.758969	3.944038	-3.207934
H	5.980881	2.896759	-5.445890
H	4.253997	1.299094	-6.239269
H	2.298845	0.759265	-4.789226

XV) Species A

C	0.180273	-0.377673	-0.154656
C	-0.080056	0.074764	1.155119
C	1.008219	0.562646	1.906146
C	2.286718	0.622132	1.368626
C	2.529806	0.203067	0.061712
C	1.462271	-0.298899	-0.682531
N	-1.353153	0.093283	1.687669
C	-2.489805	-0.601698	0.934177
O	-2.346827	-1.865080	0.687704
C	-3.729891	-0.264993	1.823364
C	-4.218629	1.021887	2.087501
C	-5.355427	1.211600	2.889962
C	-6.005316	0.116853	3.442023
C	-5.525133	-1.168526	3.182739
C	-4.406999	-1.347331	2.378475

C	-3.602915	2.202354	1.534838
N	-3.207719	3.213075	1.123508
H	-5.716861	2.220080	3.071890
H	-6.884565	0.264727	4.063850
H	-6.034371	-2.030594	3.609012
H	-3.993214	-2.318486	2.113584
H	-2.592978	0.077758	0.037700
H	-1.359885	-0.117846	2.679760
H	0.826898	0.915734	2.919739
H	3.101590	1.010437	1.977228
H	3.529447	0.254815	-0.362187
H	1.634772	-0.660392	-1.694736
H	-0.622103	-0.857756	-0.702199

XVI) Species C

C	-0.063423	0.100340	-0.156777
C	-0.218030	-0.347692	1.162127
C	0.937802	-0.593705	1.903263
C	2.199362	-0.396462	1.360962
C	2.341769	0.053431	0.046608
C	1.210167	0.299119	-0.713677
C	-1.596473	-0.544613	1.823268
O	-1.972237	0.736717	2.335015
C	-1.201110	0.368559	-0.998134
N	-2.065811	0.601277	-1.736264
N	-1.595833	-1.444587	2.913331
C	-1.564791	-2.761289	2.652330
C	-1.650172	-3.676173	3.750589
C	-1.629779	-5.045461	3.578370
C	-1.520347	-5.620490	2.303960
C	-1.426693	-4.758040	1.214099
C	-1.440198	-3.376374	1.366181

H	1.294658	0.641980	-1.740845
H	3.328581	0.201782	-0.383160
H	3.083185	-0.602425	1.959958
H	0.796353	-0.969682	2.912283
H	-1.740267	-3.242016	4.744119
H	-1.702277	-5.689421	4.455379
H	-1.503412	-6.699472	2.172469
H	-1.331606	-5.170224	0.209158
H	-1.341865	-2.754159	0.480268
H	-2.313993	-0.807495	1.018329
H	-2.100721	0.502389	3.268178

XVII) Species TS formation of A from 1 and $PhNH^-$

C	-0.685594	1.071075	1.056802
C	-1.256291	-0.118400	0.582069
C	-2.483005	-0.032988	-0.101250
C	-3.085949	1.211070	-0.344930
C	-2.482533	2.376213	0.099672
C	-1.276715	2.297009	0.805452
C	-0.539226	-1.405326	0.865096
O	0.281176	-1.461112	1.786266
C	-3.163413	-1.209034	-0.569408
N	-3.771214	-2.120627	-0.954059
N	0.426860	-1.157653	-1.022518
C	1.606996	-0.548117	-0.739128
C	2.578158	-1.228552	0.045075
C	3.791575	-0.640363	0.361603
C	4.111732	0.650123	-0.071156
C	3.166908	1.348904	-0.822548
C	1.941288	0.776958	-1.135632
H	-4.031010	1.245042	-0.879499
H	-2.948484	3.338394	-0.092852

H	-0.801338	3.206675	1.164139
H	0.249802	0.982521	1.598668
H	-1.023995	-2.311651	0.468921
H	-0.137398	-0.493697	-1.557432
H	1.211322	1.342560	-1.714234
H	3.385395	2.360592	-1.163585
H	5.070547	1.099530	0.176213
H	4.508782	-1.198476	0.962195
H	2.333854	-2.226055	0.393059

XVIII) Species E

N	0.685540	0.644738	1.082519
C	0.506581	-0.759797	0.535758
C	1.922084	-1.163577	0.164939
C	2.800840	-0.148484	0.532276
C	1.929438	0.905156	1.069710
C	4.173025	-0.253359	0.338721
C	4.654739	-1.418425	-0.255997
C	3.774430	-2.432181	-0.650558
C	2.399498	-2.311710	-0.446838
N	-0.388106	-0.941069	-0.536745
C	-1.695718	-0.901987	-0.231085
C	-2.636985	-1.228675	-1.261483
C	-3.999209	-1.265249	-1.041672
C	-4.544612	-0.972103	0.217179
C	-3.658572	-0.626093	1.236282
C	-2.284083	-0.579516	1.034851
O	2.464630	2.072453	1.511257
H	-1.645329	-0.248209	1.848060
H	-4.050208	-0.370322	2.222234
H	-5.618444	-0.999686	0.387091
H	-4.661545	-1.529577	-1.867246

H	-2.223748	-1.463909	-2.240169
H	0.229599	-1.339279	1.449726
H	1.701770	-3.076739	-0.773856
H	4.172072	-3.323050	-1.131788
H	5.722041	-1.536599	-0.427585
H	4.844710	0.548295	0.633151
H	1.697867	2.596842	1.787308

XIX) Species Ts-C-E

C	0.544161	0.944573	1.458375
C	1.526474	1.773336	0.904160
H	1.627847	2.773708	1.314896
C	2.346269	1.319093	-0.119682
H	3.099723	1.977761	-0.545977
C	2.212659	0.011462	-0.588244
H	2.862952	-0.358390	-1.377592
C	1.248457	-0.821627	-0.032405
H	1.146040	-1.849413	-0.371475
C	0.399829	-0.358959	0.975888
C	-0.240008	1.445835	2.605209
N	-0.218198	2.364503	3.371548
C	-0.658561	-1.278574	1.501737
H	-1.630545	-1.201545	1.002974
N	-0.293073	-2.370778	2.106278
C	-1.245327	-3.373432	2.271209
C	-1.345961	-4.019695	3.515764
H	-0.699326	-3.673770	4.317070
C	-2.243619	-5.059439	3.718497
H	-2.303329	-5.532408	4.696519
C	-3.066586	-5.500098	2.681906
H	-3.765577	-6.317290	2.839743
C	-2.968927	-4.881782	1.437789

H	-3.593528	-5.220013	0.613405
C	-2.072161	-3.838779	1.231860
H	-1.987479	-3.380774	0.249498
O	-1.418984	0.166125	2.692645
H	-1.298800	-0.140130	3.601966

XX) Species F

C	1.072660	0.676352	-0.480446
C	0.106061	-0.216751	-0.002251
C	0.364685	-0.936100	1.167851
C	1.559063	-0.736740	1.855327
C	2.517246	0.152014	1.381646
C	2.268453	0.850432	0.201715
N	-1.106240	-0.325358	-0.707055
C	-2.163211	-1.153251	-0.260617
O	-1.876425	-2.574078	-0.343335
C	-3.405322	-0.990297	-1.096154
C	-4.118796	0.155814	-1.413214
C	-5.242458	0.017789	-2.225883
C	-5.631079	-1.236148	-2.707414
C	-4.901527	-2.378092	-2.389673
C	-3.783868	-2.232445	-1.576968
C	-2.805147	-3.233542	-1.086509
N	-2.716922	-4.485958	-1.242439
H	-0.338990	-1.681171	1.522482
H	1.745153	-1.304531	2.762308
H	3.450067	0.293270	1.918094
H	3.006915	1.545710	-0.186939
H	0.871775	1.243258	-1.386189
H	-1.012323	-0.278099	-1.712991
H	-2.345425	-0.942955	0.798747
H	-5.199400	-3.351107	-2.770427

H	-6.509922	-1.317885	-3.339359
H	-5.824185	0.895170	-2.491435
H	-3.806686	1.127417	-1.042995
H	-3.489475	-4.817801	-1.818183

XXI) Species Final Product

C	0.000136	0.000795	0.000356
C	-0.000176	-0.001793	1.400866
H	0.931775	-0.001490	1.957531
C	-1.203415	-0.010483	2.102263
H	-1.178728	-0.013765	3.188176
C	-2.421444	-0.006394	1.433968
H	-3.354770	-0.007065	1.987298
C	-2.423712	0.002657	0.040138
H	-3.364227	0.007878	-0.503119
C	-1.232057	0.005123	-0.669232
H	-1.246375	0.003705	-1.756525
N	1.178227	-0.036016	-0.757369
H	1.065046	0.347800	-1.687497
C	2.442521	0.321372	-0.169070
H	2.621301	-0.348870	0.679944
C	3.570523	0.211604	-1.172549
C	3.942427	-0.868459	-1.958038
H	3.402533	-1.809152	-1.908455
C	5.028183	-0.703209	-2.818556
H	5.344161	-1.531847	-3.445261
C	5.716148	0.511748	-2.888974
H	6.557967	0.610232	-3.567515
C	5.328755	1.594259	-2.102574
H	5.842879	2.549116	-2.145805
C	4.248635	1.419803	-1.251324
C	3.608210	2.403598	-0.327113

O	3.916299	3.560910	-0.127928
N	2.573751	1.712132	0.255885
H	1.961165	2.144507	0.930052

XXII) Species Ts-F-final-product

C	2.177564	0.622260	0.086906
C	1.741004	-0.704018	0.191817
C	2.623359	-1.776221	0.084897
C	3.962809	-1.507822	-0.180882
C	4.397967	-0.190354	-0.326387
C	3.511269	0.877494	-0.195364
C	0.291181	-0.846821	0.401702
N	-0.545097	-0.399535	-0.499899
C	-1.957212	-0.348173	-0.310889
C	-2.677331	-1.504282	-0.030832
C	-4.051880	-1.410284	0.161558
C	-4.690084	-0.176898	0.059536
C	-3.953877	0.969792	-0.231406
C	-2.578208	0.894437	-0.416537
C	1.034151	1.589489	0.327381
N	0.561720	1.458941	1.547276
O	0.527325	2.141208	-0.691286
H	-2.172855	-2.465082	0.015182
H	-4.625285	-2.305608	0.379456
H	-5.763551	-0.109736	0.205280
H	-4.450164	1.932062	-0.305746
H	-1.967883	1.773238	-0.612097
H	-0.162740	0.341341	-1.115950
H	-0.124085	-1.434926	1.215918
H	2.274602	-2.800406	0.185461
H	4.668487	-2.326758	-0.277381
H	5.446407	0.005664	-0.531197

H	3.858818	1.901412	-0.287211
H	-0.300972	2.011695	1.597941

XXIII) Species Adduct-A-CH_3CN

C	-3.502067	-0.736403	-0.771701
C	-3.104896	0.164544	0.230880
C	-4.038194	0.497913	1.228908
C	-5.320481	-0.032252	1.215420
C	-5.718941	-0.902061	0.201854
C	-4.795332	-1.246102	-0.782248
N	-1.856668	0.770410	0.238809
C	-0.827246	0.390648	-0.735039
C	0.403453	1.241986	-0.479445
C	1.493653	0.417992	-0.262171
C	2.743873	0.967647	0.012405
C	2.874516	2.352921	0.059098
C	1.768653	3.180874	-0.156289
C	0.518422	2.624898	-0.424437
C	1.166368	-1.029699	-0.333485
N	1.742267	-2.081771	-0.207870
O	-0.423165	-0.918975	-0.686352
H	-2.772551	-1.077400	-1.496958
H	-5.075769	-1.946310	-1.565761
H	-6.724052	-1.314154	0.188285
H	-6.018479	0.244460	2.002515
H	-3.743477	1.193132	2.012274
H	-1.481011	0.915785	1.167714
H	-1.262234	0.637262	-1.721875
H	3.597268	0.318191	0.186799
H	3.846378	2.793535	0.267658
H	1.884778	4.261359	-0.111446
H	-0.353332	3.256186	-0.582088

H	3.643678	-2.384722	0.155908
C	4.682124	-2.757069	0.346004
H	4.658570	-3.415670	1.219480
H	5.006536	-3.336007	-0.524015
C	5.612656	-1.662256	0.581551
N	6.344690	-0.781966	0.767699

XXIV) Species Adduct AH-CH_2CN

O	-0.351370	-0.999134	-0.611479
C	-0.775638	0.360285	-0.680193
C	0.475117	1.164683	-0.444162
C	1.529811	0.287250	-0.252225
C	1.018427	-1.103673	-0.353320
C	2.814947	0.750209	0.004994
C	3.005805	2.128322	0.062699
C	1.940657	3.014357	-0.125317
C	0.653854	2.539070	-0.380045
N	-1.792780	0.698206	0.271897
C	-3.066236	0.131981	0.231872
C	-3.464089	-0.765554	-0.768470
C	-4.771413	-1.240195	-0.800443
C	-5.700054	-0.859494	0.163033
C	-5.299285	0.012179	1.173928
C	-4.003930	0.508042	1.206373
N	1.553621	-2.231971	-0.259507
C	4.626978	-2.365985	0.318231
C	5.430572	-1.234741	0.496652
N	6.009624	-0.214564	0.629987
H	-2.740416	-1.129973	-1.488532
H	-5.056522	-1.938697	-1.582742
H	-6.715163	-1.244146	0.135392
H	-6.004625	0.317405	1.942676

H	-3.704979	1.205567	1.985360
H	-1.434582	0.870095	1.202079
H	-1.194424	0.522629	-1.681754
H	3.645785	0.068880	0.159296
H	4.006695	2.499312	0.262156
H	2.115659	4.085990	-0.070777
H	-0.182329	3.219346	-0.519088
H	2.586329	-2.163597	-0.070133
H	4.515716	-2.983994	1.211406
H	4.867998	-2.953212	-0.570077

XXV) Species Ts-H-trans-from-CH_3CN-to-A

C	0.207627	-0.852378	1.202537
C	-0.065837	-0.527465	-0.134597
C	0.962919	0.035664	-0.907930
C	2.214527	0.278059	-0.360435
C	2.474289	-0.020218	0.976021
C	1.460983	-0.586864	1.743727
N	-1.323687	-0.695976	-0.706102
C	-2.413473	-1.304503	0.025713
C	-3.659395	-1.322493	-0.825232
C	-4.098456	-2.631193	-0.935228
C	-5.228717	-2.932414	-1.690107
C	-5.898184	-1.886744	-2.321250
C	-5.447141	-0.568027	-2.210508
C	-4.311697	-0.273243	-1.457811
C	-3.190801	-3.546812	-0.177662
N	-3.147705	-4.761183	0.023465
O	-2.192520	-2.640652	0.393286
H	-0.542996	-1.362412	1.795211
H	1.650881	-0.852493	2.780653
H	3.452696	0.173292	1.406015

H	2.992430	0.712463	-0.983713
H	0.761538	0.292171	-1.945633
H	-1.304089	-0.961955	-1.682289
H	-2.551998	-0.703214	0.936884
H	-5.586263	-3.951958	-1.792812
H	-6.783633	-2.107906	-2.911023
H	-5.983769	0.230875	-2.716557
H	-3.941597	0.745447	-1.370772
H	-4.060060	-5.554862	-0.472781
C	-4.880595	-6.649178	-0.849290
H	-4.188692	-7.350593	-1.328153
H	-5.226500	-7.070403	0.100991
C	-5.982743	-6.357249	-1.706633
N	-6.859915	-6.026683	-2.406880

XXVI) Species TS A-C

C	0.140448	-0.234129	-0.113781
C	-0.043764	-0.183618	1.294024
C	1.041712	0.324020	2.052906
C	2.209231	0.757055	1.436341
C	2.368366	0.705371	0.052934
C	1.311380	0.199635	-0.710126
N	-1.208212	-0.640687	1.820415
C	-1.746889	-0.231832	3.127752
O	-3.053644	-0.060783	2.724761
C	-1.599270	-1.359320	4.145235
C	-2.334719	-2.530046	3.926186
C	-2.238001	-3.615477	4.777938
C	-1.401629	-3.564081	5.900317
C	-0.677522	-2.414309	6.152020
C	-0.777545	-1.314326	5.278319
C	-0.015785	-0.140864	5.613701

N	0.618598	0.772266	5.948504
H	-0.029936	-2.343733	7.021209
H	-1.325551	-4.414466	6.572317
H	-2.818070	-4.513655	4.579029
H	-2.993580	-2.532773	3.064488
H	-0.675876	-0.625449	-0.716219
H	1.404264	0.145356	-1.794254
H	3.287180	1.044105	-0.418556
H	3.014994	1.142195	2.059426
H	0.964190	0.371565	3.134407
H	-2.429390	-0.356632	1.634202
H	-1.262949	0.684172	3.527224

B) Additional details on the proposed reaction mechanisms.

The scheme reported in the Figure 2 is initiated by the Species 1 and 2a (entries IV and VII) of the previous collection. These two species form the intermediate B, a species (entry X of the above collection) characterized by a relatively long interpartnem distance (2.5 Angstrom) and, hence, thermodynamically, and kinetically unstable toward the backdissociation to the reatants. The TS for the formation of B (Ts-1) was located not analytically but only approximately by following the minimum energy path leading 1 and 2a to B.

Species B, in principle – although not in practice – might potentially form the isoenergetic species AH overpassing a very high barrier characterizing the Ts-2 (entry XIV).

Concerning the scheme (a) of the Figure 3, in the electrochemical route in the left side of the Figure, initiated by the formation of CH_2CN^- by 2a deprotonation to give $PhNH^-$, we have observed the formation of A from the addition of $PhNH^-$ to 1 through a barrier of only 11.0 k/J/mol corresponding to the transition structure XVII of the above collection. Species A then interconverts to C through a 95 kJ/mol barrier corresponding to the transition structure XXVI of the above collection. Species C then transforms into D through the transition structure XIX of the above collection. Finally, species D, with a barrierless process, collaps into E (entry XVIII) which eventually produces the conjugated basis of the final product by an acid/base reaction with CH_3CN, hence restoring the initial base CH_2CN^-.

Concerning the right side part of the scheme (a) of the Figure 3 we have also reported, for the sake of completeness, the potential – although practically irrelevant – step of formation of A through an acid base reaction of the solvent with the species B whose presence has been already indicated as a rather unlikely both by a thermodynamic and kinetic point of view in the present temperature and solvent conditions.

In the scheme (b) of the same Figure we have indicated the alternative channel where species A is protonated by the solvent with process characterized – in the previous collection – by the reactant

XXIII, the product XXIV and the transition structure XXV with a barrier of 41 kJ/mole. Subsequently species F, i.e. the species XXIV without the CH_2CN^- moiety, might unimolecularly interconvert into the final product trhough a transition structure (entry XXII) actually characterized by a rather high barrier of 130 kJ/mol.
